# Identification of cardiovascular and molecular prognostic factors for the morbidity and mortality in COVID-19-sepsis (ICROVID): Protocol for a prospective multi-centre cohort study

**DOI:** 10.1371/journal.pone.0269247

**Published:** 2022-06-03

**Authors:** Charles Neu, Philipp Baumbach, André Scherag, Andreas Kortgen, Juliane Götze, Sina M. Coldewey

**Affiliations:** 1 Department of Anaesthesiology and Intensive Care Medicine, Jena University Hospital, Jena, Germany; 2 Septomics Research Centre, Jena University Hospital, Jena, Germany; 3 Centre for Sepsis Control and Care, Jena University Hospital, Jena, Germany; 4 Institute of Medical Statistics, Computer and Data Sciences, Jena University Hospital, Jena, Germany; UNITED KINGDOM

## Abstract

**Introduction:**

Severe COVID-19 constitutes a form of viral sepsis. Part of the specific pathophysiological pattern of this condition is the occurrence of cardiovascular events. These include pulmonary embolism, arrhythmias and cardiomyopathy as manifestations of extra-pulmonary organ dysfunction. Hitherto, the prognostic impact of these cardiovascular events and their predisposing risk factors remains unclear. This study aims to explore this question in two cohorts of viral sepsis–COVID-19 and influenza–in order to identify new theragnostic strategies to improve the short- and long-term outcome of these two diseases.

**Methods and analysis:**

In this prospective multi-centre cohort study, clinical assessment will take place during the acute and post-acute phase of sepsis and be complemented by molecular laboratory analyses. Specifically, echocardiography and cardiovascular risk factor documentation will be performed during the first two weeks after sepsis onset. Aside from routine haematological and biochemical laboratory tests, molecular phenotyping will comprise analyses of the metabolome, lipidome and immune status. The primary endpoint of this study is the difference in 3-month mortality of patients with and without septic cardiomyopathy in COVID-19 sepsis. Patients will be followed up until 6 months after onset of sepsis via telephone interviews and questionnaires. The results will be compared with a cohort of patients with influenza sepsis as well as previous cohorts of patients with bacterial sepsis and healthy controls.

**Ethics and dissemination:**

Approval was obtained from the Ethics Committee of the Friedrich Schiller University Jena (2020-2052-BO). The results will be published in peer-reviewed journals and presented at appropriate conferences.

**Trial registration:**

DRKS00024162.

## Introduction

### Background

In sepsis, a dysregulated host response resulting from infection leads to functional impairment or failure of one or more organ systems [1]. The underlying infection can be caused by a variety of pathogens: mainly bacteria, but also viruses, fungi, and parasites [2]. The highest incidence and mortality of sepsis is recorded in areas with the lowest socio-demographic index [3], however, sepsis is also a frequently underestimated disease in countries with a high standard of living. Here, major surgery or immunomodulatory chemotherapies, which often lead to impaired immune function in patients are risk factors for sepsis [4]. A very recent example of the great social, economic, and political importance of sepsis is the current coronavirus disease 2019 (COVID-19) pandemic. It was triggered by an outbreak of infections with the new beta-coronavirus SARS-CoV-2 (severe acute respiratory syndrome coronavirus 2) in December 2019 [5]. Within a very short period of time, the COVID-19 pandemic has pushed healthcare systems worldwide to their limits.

Despite extensive research over the past decades, no clinical trial has yet led to the implementation of a causal therapy for sepsis in clinical practice. However, large clinical trials in the context of the COVID-19 pandemic, proved an anti-inflammatory treatment with tocilizumab to be beneficial for the course of severe COVID-19 disease/sepsis in patients with severe hyperinflammation [6]. Furthermore, recent scientific findings suggest that patients with sepsis could benefit from being stratified according to their clinical and molecular phenotype and risk profile, resulting in a personalized therapy [4]–an approach previously used in the field of cancer therapy [7]. Patients with sepsis form a very heterogeneous patient population [4]. Therefore, it seems particularly important that clinical trials take into account the diversity of patients in terms of individual medical history, clinical presentation, individual molecular and immunological host response, and causative pathogen. Innovative translational research approaches are needed to identify novel targets against which diagnostic and therapeutic strategies can be developed in this severe systemic disease [8].

Patients with COVID-19 sepsis appear to develop a specific phenotype that requires further clinical and molecular investigation. Typical early symptoms of COVID-19 include fever and dry cough [9] as well as a distinct loss of taste and smell [10]. Despite a uniform causative agent, the severity of disease progression after SARS-CoV-2 infection varies inter-individually, of which the causative molecular mechanisms remain unknown. In addition to asymptomatic infections and mild courses of COVID-19, severe courses with the development of pneumonia with marked hypoxia, acute respiratory distress syndrome (ARDS), or multi-organ failure with or without death have been observed. The World Health Organization reported on the initial outbreak in China that about 80% of laboratory-confirmed SARS-CoV-2-positive patients show mild to moderate courses of disease, while severe courses occur in 13.8% and critical courses in 6.1% [5]. By definition, these very severe courses of COVID-19 disease can be subsumed under the term sepsis [1]. These patients experience the above-mentioned pronounced lung damage with severe oxygenation disturbance accompanied by a severe inflammatory reaction, a so-called cytokine storm. They often require invasive ventilation, but other organ dysfunctions, such as renal and liver failure, must also be treated. Thus, the group of sepsis patients with COVID-19 can be considered a special phenotype of sepsis.

Several authors have also reported a high incidence of cardiac complications [11], the occurrence of marked endothelial damage [12], and coagulation disorders with an accumulation of life-threatening thromboembolic events [11, 13–15] in COVID-19. Cardiovascular complications include myocardial infarction, arrhythmias, pericardial tamponade, and pulmonary emboli (reviewed in [16]). Myocardial injury, often seen as an increase in the cardiac enzyme troponin, occurs in approximately 20% of critically ill COVID-19 patients [17–19]. In a monocentric study, laboratory-detected cardiac injury was associated with significantly increased mortality [18], however the timing of follow-up was variable ranging from 1 to 37 days, complicating the accurate estimation of mortality. In addition, cardiac function was not monitored via cardiac imaging. Hence, the exact effect of COVID-19-associated myocardial injury on patient long-term outcome remains unknown. Functional abnormalities of the heart have also been described at 12% [9]. The causes of these are not clear. Cardiac decompensation of pre-existing heart disease or myocarditis caused by the virus is conceivable. In autopsies of 39 decedents with COVID-19, significant amounts of SARS-CoV-2 virus were detected in cardiac tissue in 41% [20]. Due to the absence of a cellular inflammatory response in these samples, the presence of myocarditis in these patients remains unclear. Lastly, viral sepsis may also cause septic cardiomyopathy, which is also associated with a reduction in ejection fraction [21]. The molecular mechanisms and the significance of cardiovascular events for the medium- and long-term course of disease in patients with COVID-19 sepsis have not yet been elucidated.

Severe COVID-19 is not the only viral sepsis with cardiovascular complications. Authors describe that patients with influenza also have a higher risk of suffering cardiovascular complications [22, 23]. Extrapulmonary organ dysfunction, such as cardiac involvement due to the development of myocarditis or cardiomyopathy, has been described for influenza [24]. Comparing these two entities of viral sepsis may therefore contribute towards a better understanding of each individual condition and offer new potential treatment strategies in these severe diseases.

### Aim

This clinical trial aims to characterise thoroughly and systematically the clinical and molecular understanding of severe COVID-19, including the medium- and long-term course of the disease, using clinical investigation and state-of-the-art laboratory analyses. Taking into account the current circumstances and development of the COVID-19 pandemic, in particular the numbers of infections and frequency of severe courses, the project is designed as a multi-centre prospective clinical study. To assess the specificity of findings for COVID-19, a second cohort of patients with influenza sepsis will be enrolled, analysed and compared with patients with COVID-19.

## Methods and analysis

### Study design

This study is a prospective multi-centre cohort study of patients with sepsis. Patient recruitment commenced in March 2021 aiming to include up to 160 patients with COVID-19-associated sepsis and up to 160 patients with influenza virus associated sepsis. The last follow-up is planned for December 2022.

### Study setting

Patient recruitment takes place in intensive care units (ICUs) in up to 10 hospitals in Germany. Follow-up assessments will take place in the form of phone interviews and questionnaires.

### Study population

Patients receiving intensive care therapy with either COVID-19-associated sepsis or influenza-associated sepsis.

### Sample size

The study aims to enrol up to 160 patients in each study arm.

### Eligibility criteria

Box 1 lists the inclusion and exclusion criteria for both patient groups.

**Box 1. Eligibility criteria**

**Table:** 

**Inclusion criteria**		
▶	patient age ≥ 18 years	
▶	written informed consent of the patient or his legal representative	
▶	confirmed SARS-CoV-2 infection OR confirmed influenza virus infection	
▶	respiratory symptoms	
▶	indication for ICU-therapy	
▶	sepsis or septic shock according to Sepsis-3 criteria [1]	
▶	first infection-associated organ dysfunction (= sepsis diagnosis) not older than 4 days (first blood sample within 4 days after sepsis diagnosis)	
**Exclusion criteria**		
▶	cardiac surgery ≤ 12 months	
▶	significant pre-existing heart condition	
	○	*endocarditis*
	○	*higher degree valve disorders (severe valvular heart disease*, *symptomatic* *aortic valve stenosis*, *moderate mitral regurgitation with reduced ejection* *fraction)*
	○	*congenital heart defect (e*.*g*. *transposition of the great arteries*, *tetralogy of* *Fallot*, *atrioventricular septal defect)*
	○	*haemodynamically relevant shunting heart defect*
	○	reduced cardiac output (left ventricular ejection fraction < 45% or 10% below norm*) prior to onset of sepsis
	○	*pulmonary arterial hypertension prior to onset of sepsis*
	○	*myocardial infarction 12 months prior to onset of sepsis*
	○	*history of heart transplantation*
▶	cardiopulmonary resuscitation 4 weeks prior to onset of sepsis	
▶	history of pneumonectomy	
▶	Child C liver cirrhosis	
▶	contraindications for transoesophageal echocardiography and insufficient quality of transthoracic echocardiography	
▶	terminal chronic kidney disease with dialysis	
▶	sepsis within 8 months prior to onset of sepsis	
▶	pregnancy/breast-feeding	
▶	therapy limitation/do-not-resuscitate order	
▶	remaining life expectancy < 6 months due to other causes than sepsis	
▶	prior participation in this study	

* left ventricular ejection fraction >52% in men, >54% in women according to the American Society of Echocardiography and the European Association of Cardiovascular Imaging [25].

### Study outline

**Fig 1:**
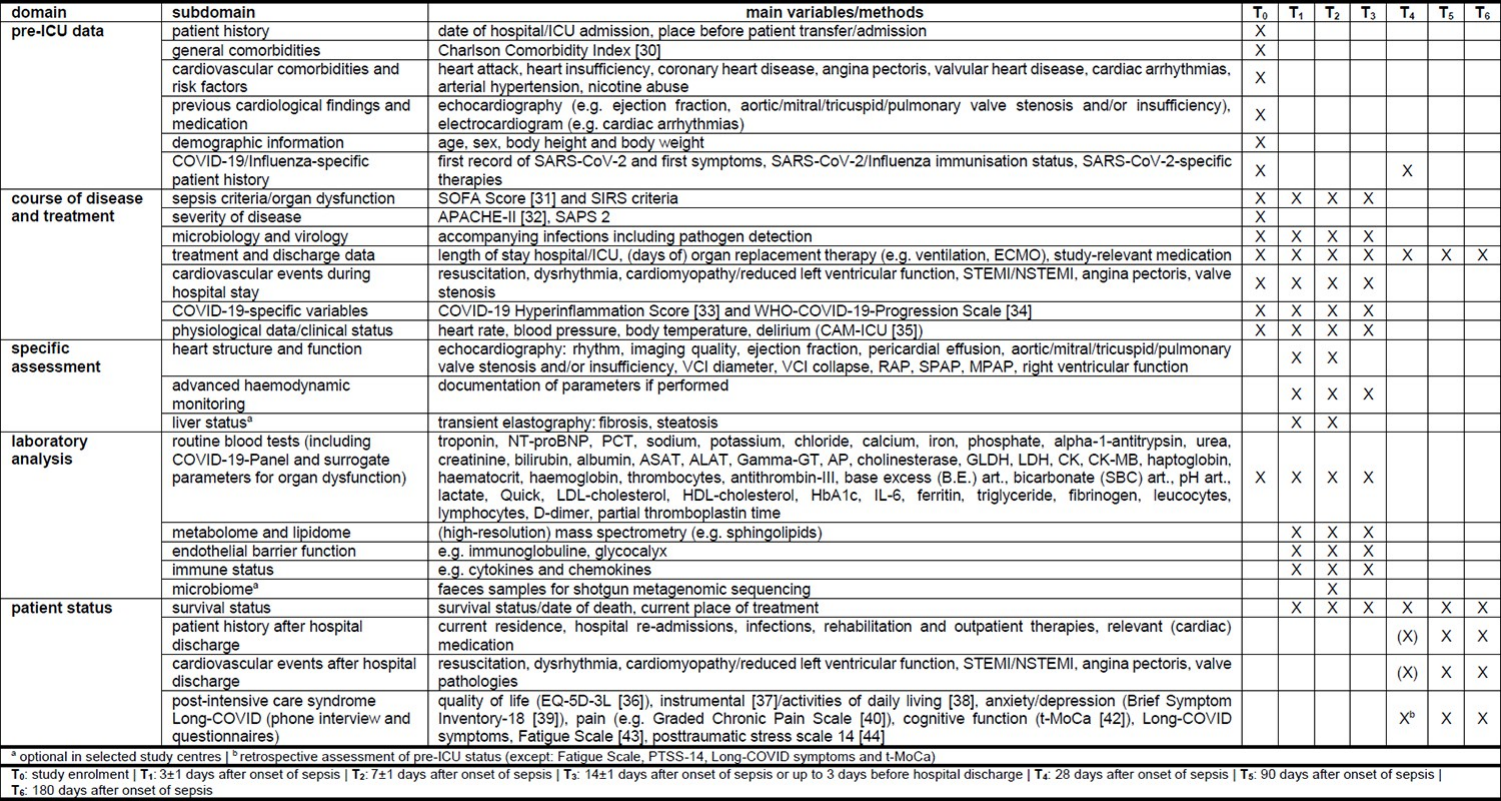
Study outline.

Fig 1 shows an overview of the scheduled clinical and laboratory analyses for both patient groups. The planned study visits during the hospital stay are study enrolment (T_0_), acute phase of sepsis (T_1_: 3±1 days and T_2_: 7±1 days after sepsis onset), 14 days after sepsis onset or 3 days or fewer before hospital discharge (T_3_). The planned follow-up visits are 28 days (T_4_), 90 days (T_5_) and 180 days (T_6_) after sepsis onset and will take place as phone interviews and questionnaire surveys.

After hospital discharge, the study team will contact the patients. At follow-up time points, patient digital records are consulted for information regarding the patient status. In case of current readmission to the hospital, the patient is contacted directly. In all other cases, a member of the study team will contact the patient via telephone. If a direct contact is not possible and prior permission was granted, a member of the study team will contact the patient’s legal proxy, next of kin or the patient’s family doctor. Should this not lead to the assessment of the patient’s status, the study team contacts the patient via letter. Should all these measures fail to establish contact by the end of the follow-up period or the patient revokes consent of participation in the study and data usage, the patient is considered as lost to follow up.

### Study endpoints

The primary endpoint of this study is the difference in 3-month mortality of COVID-19-associated sepsis patients with or without septic cardiomyopathy. In this study, septic cardiomyopathy is defined as a new systolic dysfunction (reduced left ventricular ejection fraction <52% in men, <54% in women [25] or 10% reduction if previously reduced) during the acute phase of sepsis (T_1_ and/or T_2_) [26–29]. Box 2 lists the secondary endpoints.

**Box 2. Secondary and further study outcomes**

**Table:** 

▶	differences in mortality rates between COVID-19-sepsis patients with *vs*. without septic cardiomyopathy^1^ 6 months after diagnosis of COVID-19-sepsis (T_6_)
▶	incidence of cardiovascular events in patients with COVID 19-sepsis during the acute (T_1_, T_2_), post-acute (T_3_ –T_4_) and long-term course of disease (T_5_ –T_6_)
▶	differences in the incidence rates of septic cardiomyopathy^1^ during the acute course of disease (T_1_ and/or T_2_) in patients with COVID-19-sepsis and patients with influenza-sepsis
▶	differences in the incidence rates of cardiovascular events in patients with COVID 19-sepsis and patients with influenza-sepsis during the acute (T_1_, T_2_), post-acute (T_3_ –T_4_) and long-term course of disease (T_5_ –T_6_)
For further research questions, patients with COVID-19 sepsis and patients with influenza sepsis as well as pre-existing cohorts of patients with non-COVID-19-associated sepsis and healthy volunteers will be compared. For this purpose, further outcome measures including cardiovascular risk factors and function, liver/kidney stiffness, organ dysfunction, immune status, metabolome, lipidome and general functional level, will be analysed:	
▶	differences in the occurrence of septic cardiomyopathy^1^ between patients with COVID-19-sepsis and non-COVID-19-associated sepsis during the acute course of disease (cumulatively T_1_ and T_2_)
▶	identification of COVID 19-specific clinical and molecular changes in the acute and post-acute course of disease
▶	analysis of potential group differences in the acute and post-acute course of disease (complete sample and stratified by the occurrence of septic cardiomyopathy^1^) to identify variables with potential diagnostic relevance in COVID-19-sepsis
▶	characterisation of the acute and post-acute course of disease in patients with COVID-19-sepsis (complete sample and stratified by the occurrence of septic cardiomyopathy^1^) to explore potential surrogate parameters for the occurrence of cardiac dysfunction
▶	comparison of the incidence of right heart failure during the acute course of disease (T_1_ and/or T_2_) in patients with COVID-19 sepsis and influenza sepsis
▶	association of right heart failure and mortality and morbidity in COVID-19-sepsis and Influenza-sepsis.
▶	analysis of potential group differences in the mid- (T_5_) and long-term (T_6_) course of disease in patients with COVID-19-sepsis (complete sample and stratified by the occurrence of septic cardiomyopathy^1^) und the other cohorts for the identification of potential biomarkers of the acute and post-acute course of disease and potential therapeutic target structures, which might further be analysed pre-clinically
▶	analysis of the mid- and long-term morbidity in patients with COVID-19-sepsis regarding physical and cognitive performance and interference, health-related quality of life and type and frequency of cardiovascular events after ICU admission (complete sample and stratified by the occurrence of septic cardiomyopathy^1^)
▶	identification of potential predictors of mid- (T_5_) and long-term (T_6_) mortality and morbidity after COVID-19-sepsis. The focus lies on data from the acute (T_1_, T_2_) and post-acute (T_3_) course of disease

^1^ defined as new systolic dysfunction: reduced left ventricular ejection fraction (<52% in men, <54% in women [25] or 10% reduction if previously reduced) during the acute phase of sepsis (T_1_ and/or T_2_) [26–29].

### Clinical and laboratory assessments

Fig 1 summarizes the main clinical and laboratory assessments. Patient history (before and after ICU-stay) will be gathered from medical documents, hospital database checks and patient interviews [30–35]. Clinical and physiological data will be obtained from the patients’ digital charts or by direct measurement and documentation. Next to routine blood tests, surrogate parameters of cardiac and other organ dysfunctions, endothelial barrier/glycocalyx dysfunction as well as analysis of metabolome, lipidome, immune status, and epigenetic modifications of immune cells will be performed in specific blood and urine tests (e.g. high resolution mass spectrometry). The microbiome is analysed in stool samples. Cardiac function will be assessed with transthoracic echocardiography. Aside from standard 2D techniques, heart volumes and output will be quantified with 3D-echocardiography and speckle-tracking. If the transthoracic route does not permit adequate assessment (e.g. due to oedema), transoesophageal echocardiography (TOE) will be performed. If performed, data from advanced haemodynamic monitoring will be documented. Transient elastography of the liver will be performed using a FibroScan® 430 Mini+ (Echosens, Paris, France) in selected study centres.

The following instruments are used to assess symptoms of post-intensive care syndrome and long-COVID: EQ-5D-3L [36] (health related quality of life), instrumental [37]/activities of daily living [38] (physical function), Brief Symptom Inventory-18 [39] (symptoms of anxiety/depression), Graded Chronic Pain Scale [40] and DN4 [41] (pain), t-MoCa [42] (cognitive function), Long-COVID symptom check list, Fatigue Scale [43] (fatigue), posttraumatic stress scale 14 (PTSS-14 [44], posttraumatic stress disorder).

### Sample size calculation and statistical analysis

The study is primarily exploratory and study size planning is based on similar considerations as in a previous observational study of patients with sepsis [45]. The focus is on mortality differences between patients with COVID-19 sepsis with or without septic cardiomyopathy at 3 months (primary endpoint). During study planning, an overall 3-month mortality of 50% [46–49] and an incidence of 33% for reduced left ventricular ejection fraction (<50%) as a criterion for septic cardiomyopathy was assumed [27, 50–56]. If a simple χ2 test is applied at a 2-sided significance level of α = 5%, a cohort size of n = 160 patients is sufficient to detect differences (absolute risk reduction) in 3-month mortality of ≥ 22% with a statistical power of ≥ 80%. In case of a lower 3-month mortality in the group of COVID-19 patients treated in ICU without cardiomyopathy of, for example, 40%, similarly large differences (absolute risk reduction) can also be demonstrated with a power ≥ 80%. Case number considerations were performed using the power.prop.test function in R (version 4.0.2, Vienna, Austria [57]).

In descriptive analyses, means or medians will be reported as measures for central tendency in continuous variables, respectively. As a measure of dispersion standard deviation, the 95% confidence interval or interquartile range and first and third quartiles will be shown. For dichotomous and categorical variables, absolute and relative frequencies will be presented. In case of group comparison, appropriate statistical tests depending on the distributional properties of the variables (e.g. *t*-test, Mann-Whitney *U* test) will be used. Finally, the identification of prognostic or predictive factors is primarily exploratory in nature. Univariate analyses and subsequent multivariable analyses are planned.

No interim evaluation of the complete data set is planned for the primary and secondary endpoints. Interim analyses of selected research questions can become necessary for the evaluation of hypothesis-generating further research questions and academic theses. To this end, specific limited datasets will be evaluated and may be published before completion of the study.

### Ethics

#### Informed consent

Written informed consent will be obtained from the patients by designated study physicians (S 1). However, due to the disease studied, most patients will be incapable of giving informed consent. In this case, a legal representative or proxy will be sought. In absence of both, a legal proxy will be appointed by court ruling. Until written informed consent is obtained, patients can be enrolled by consultation of an independent medical doctor assessing the patient’s incapacity and eligibility for the study.

#### Ethics approval, study registration and data management

The study in its current version (1.2, 03/06/2021) is in accordance with the Declaration of Helsinki and was approved by the Ethics Committee of the Friedrich Schiller University Jena (2020-2052-BO, 21/01/2021). It is registered at the German Clinical Trials Register (DRKS00024162). The study management software (OpenClinica, Waltham, MA, USA) conforms to the Good Clinical Practice guidelines (21 CFR Part 11) and the web-based data entry takes place via an encrypted data link (HTTPS) with pseudonymised patient identification numbers. The data is stored on servers of the Centre for Clinical Studies Jena at the Jena University Hospital. Data sets for collaborating study groups will be transferred in anonymized form. Important study protocol changes must be submitted to and approved by the appropriate committee in an amendment.

## Discussion and outlook

The ICROVID study aims to address the lack of sufficient prognostic parameters for the outcome of patients with COVID-19 sepsis. Due to its prospective multicentre design, the ICROVID study will be able to assess the influence of cardiovascular events on both the short- and long-term morbidity and mortality of COVID-19 and influenza. Comparisons of the analytical cohort with datasets of patients with bacterial sepsis may yield findings that are specific to viral sepsis. This study is limited to patients with severe COVID-19 and influenza, identified prognostic factors may not be relevant to non-severe cases. The results of this study may present a first step towards a more personalized approach to sepsis therapy.

### Dissemination

The results will be published in peer-reviewed journals and presented to the scientific community at appropriate conferences. Results will be reported in accordance with the STROBE criteria [58] for the presentation of observational studies and TRIPOD [59] criteria for the reporting of prediction modelling studies in biomedical sciences.

## Supporting information

S1 Checklist SPIRIT checklist. (PDF)

S1 File Original study protocol in German. (PDF)

S2 File English translation of original protocol. (PDF)
