# Supplementary material for: Identification of cardiovascular and molecular prognostic factors for the morbidity and mortality in COVID-19-sepsis (ICROVID): Protocol for a prospective multi-centre cohort study
Source: PLoS One. 2022 Jun 3;17(6):e0269247. doi: 10.1371/journal.pone.0269247 (PMC9165863; doi:10.1371/journal.pone.0269247)
Supplement: S1 File — (PDF) [file pone.0269247.s002.pdf]

Studienprotokoll

Identifikation kardiovaskulärer und molekularer Prognosefaktoren für die Morbidität und Mortalität bei COVID-19-Sepsis

Identification of cardiovascular and molecular prognostic factors for the morbidity and mortality in COVID-19-sepsis

Akronym: ICROVID

Protokollversion: 1.2 vom 03.06.2021

Studienleitung:

Univ.-Prof. Dr. Dr. med. Sina M. Coldewey

Translational Septomics, Zentrum für Innovationskompetenz Septomics

Klinik für Anästhesiologie und Intensivmedizin, Universitätsklinikum Jena

E-Mail: sina.coldewey@med.uni-jena.de, Telefon: 03641 9323190

Die folgenden Personen stimmen dem Inhalt des Studienprotokolls zu und bekunden das mit ihrer Unterschrift.

**Univ-Prof. Dr. Dr. med. Sina M. Coldewey**<br>**Studienleiterin (PI)**	**Datum**	**Unterschrift**
**Dr. med. Charles Neu**<br>**Stellvertreter der Studienleitung**	**Datum**	**Unterschrift**
**Philipp Baumbauch**<br>**Studienkoordinator**	**Datum**	**Unterschrift**
**Prof. Dr. med. Michael Bauer**<br>**Klinikdirektor**	**Datum**	**Unterschrift**

Inhaltsverzeichnis

1 Allgemeine Informationen 7
1.1 Beteiligte Personen, Institutionen, Gremien 7
1.2 Synopse 11
1.3 Ablauf- und Visitenplan 14
2 Abkürzungsverzeichnis 16
3 Ausgangssituation und Fragestellung 18
3.1 Sepsis 18
3.2 COVID-19-Sepsis 18
3.3 Kardiovaskuläre Komplikationen bei COVID-19 19
3.4 Mikroangiopathische Komplikationen von COVID-19 19
3.5 Fragestellungen und Ziele der Studie 21
4 Studienziele und Endpunkte 21
5 Studienpopulation 25
5.1 Einschlusskriterien 25
5.2 Ausschlusskriterien 25
5.3 Definition der Kriterien Sepsis/septischer Schock (Sepsis-3-Kriterien) 25
5.4 Definition der Kriterien septische Kardiomyopathie 26
6 Studienablauf 27
6.1 Sepsis-Patienten (COVID-19 und Influenza) 27
6.1.1 Screening und Patientenidentifikationsliste 27
6.1.2 Aufklärung und Einwilligung 27
6.1.2.1 Einwilligungsfähige Patienten 27
6.1.2.2 Nicht-einwilligungsfähige Patienten 27
6.1.2.3 Keine oder Rücknahme der Einwilligung 28
6.1.3 Dokumentation im Studienverlauf 29
6.1.4 Studienbedingte Maßnahmen 29
6.1.5 Studienende / Ende der Nachbeobachtung 29
6.2 Auswertungsphase 29
7 Beschreibung der Methoden 29
7.1 Klinische Untersuchungen 29
7.1.1 TEE/TTE 29
7.1.2 Transiente Elastographie 30
7.2 Laboruntersuchungen 30
7.2.1 Erhebung von Routineparametern 30
7.2.2 Studienbedingte Untersuchungen 30
7.3 Gesundheitsbezogene Lebensqualität und Langzeitfolgen 31
8 Unerwünschte Ereignisse 31

9 Datenmanagement und Qualitätssicherung ... 31
9.1 Patientenidentifikationsliste ... 31
9.2 Liste der Verantwortlichkeiten ... 31
9.3 Datenerhebung/Dokumentationsbögen ... 32
9.4 Datenverarbeitung ... 32
9.5 Aufbewahrung der Studienunterlagen ... 32
9.6 Datenschutz ... 32
10 Biometrie ... 33
10.1 Endpunkte ... 33
10.2 Definition von Auswertungskollektiven ... 33
10.3 Planung des Studienumfanges (Fallzahlplanung) ... 34
10.4 Zwischen-/Auswertung ... 34
10.5 Weitere statistische Analysen ... 34
10.6 Präsentation der Ergebnisse ... 34
11 Publikation / Nutzung der Ergebnisse / Registrierung der Datenerhebung ... 35
11.1 Abschlussbericht und Publikationen ... 35
11.1.1 Publikation des Studienprotokolls ... 35
11.1.2 Abschlussbericht ... 35
11.1.3 Analysen und Publikationen ... 35
11.2 Zitierweise ... 35
11.3 Autoren ... 35
11.4 Registrierung ... 35
12 Ethische Belange und administrative Regelungen ... 36
12.1 Deklaration von Helsinki und Gute Klinische Praxis ... 36
12.2 Ethik-Kommissionen ... 36
12.3 Nachträgliche Änderungen ... 36
12.4 Finanzierung ... 36
13 Literatur ... 37
14 Anhang ... 40
14.1 SOFA-Score ... 40
14.2 APACHE-Score ... 41
14.3 SAPS II Score ... 42
14.4 Charlson Comorbidity Index ... 43
14.5 CAM-ICU ... 44
14.6 COVID Hyperinflammations-Score ... 45

1 Allgemeine Informationen

1.1 Beteiligte Personen, Institutionen, Gremien

**Studienleiterin (PI)**	**Univ.-Prof. Dr. Dr. med. Sina Coldewey**<br>Klinik für Anästhesiologie und Intensivmedizin<br>Zentrum für Innovationskompetenz (ZIK) Septomics<br>Universitätsklinikum Jena<br>Am Klinikum 1, 07747 Jena<br>Tel: 03641-9323190<br>E-Mail: sina.coldewey@med.uni-jena.de
**Stellvertreter der Studienleitung**	**Dr. med. Charles Neu**<br>Klinik für Anästhesiologie und Intensivmedizin<br>ZIK Septomics<br>Universitätsklinikum Jena<br>Am Klinikum 1, 07747 Jena<br>Tel: 03641-9323166<br>E-Mail: charles.neu@med.uni-jena
**Studienkoordinator**	**Dipl.-Psych. Philipp Baumbach**<br>Klinik für Anästhesiologie und Intensivmedizin<br>ZIK Septomics<br>Universitätsklinikum Jena<br>Am Klinikum 1, 07747 Jena<br>Tel: 03641-9325798<br>E-Mail: philipp.baumbach@med.uni-jena.de
**Biometriker (beratend)**	**Prof. Dr. André Scherag**<br>Center for Sepsis Control and Care (CSCC)<br>Klinische Epidemiologie<br>Universitätsklinikum Jena<br>Salvador-Allende-Platz 27, 07747 Jena<br>Tel: 03641-[redacted]<br>E-Mail: andre.scherag@med.uni-jena

Studienärzte

Univ.-Prof. Dr. Dr. med. Sina Coldewey
Klinik für Anästhesiologie und Intensivmedizin
ZIK Septomics
Universitätsklinikum Jena
Am Klinikum 1, 07747 Jena
Tel: 03641-9323190
E-Mail: sina.coldewey@med.uni-jena.de

Dr. med. Heike Dorow
Nachwuchsgruppe Translational Septomics

Universitätsklinikum Jena
Am Klinikum 1, 07747 Jena
Tel: 03641-9323168
E-Mail: heike.dorow@med.uni-jena

Ricardo Esper Treml
Klinik für Anästhesiologie und Intensivmedizin
ZIK Septomics
Universitätsklinikum Jena
Am Klinikum 1, 07747 Jena
Tel: 03641-9323261
E-Mail: ricardo.espertreml@med.uni-jena.de

Juliane Götze
Klinik für Anästhesiologie und Intensivmedizin
ZIK Septomics
Universitätsklinikum Jena
Am Klinikum 1, 07747 Jena
Tel: 03641-9323250
E-Mail: juliane.goetze@med.uni-jena

Studienärzte (fortgesetzt)

Jan Höfer
Klinik für Anästhesiologie und Intensivmedizin
Universitätsklinikum Jena
Am Klinikum 1, 07747 Jena
Tel: 03641-9[redacted]
E-Mail: jan.hoefer@med.uni-jena.de

Prof. Dr. med. Andreas Kortgen
Klinik für Anästhesiologie und Intensivmedizin
Universitätsklinikum Jena
Am Klinikum 1, 07747 Jena
Tel: 03641-9 [redacted]
E-Mail: andreas.kortgen@med.uni-jena.de

Dr. med. Charles Neu
Klinik für Anästhesiologie und Intensivmedizin
ZIK Septomics
Universitätsklinikum Jena
Am Klinikum 1, 07747 Jena
Tel: 03641-9323166
E-Mail: charles.neu@med.uni-jena

PD Dr. med. Philipp Reuken
Klinik für Innere Medizin IV
Universitätsklinikum Jena
Am Klinikum 1
07747 Jena
Tel: 03641 9 [redacted]
E-Mail: philipp.reuken@med.uni-jena.de

Dr. med. Christiane Schmidt-Winter
Klinik für Anästhesiologie und Intensivmedizin
ZIK Septomics
Universitätsklinikum Jena
Am Klinikum 1, 07747 Jena
Tel: 03641-[redacted]
E-Mail: christiane.schmidt-winter@med.uni-jena.de

Kornel Skitek
Klinik für Anästhesiologie und Intensivmedizin
ZIK Septomics
Universitätsklinikum Jena
Am Klinikum 1, 07747 Jena
Tel: 03641-[redacted]
E-Mail: kornel.skitek@med.uni-jena.de

weitere Studienärzte (Oberärzte der Intensivstation)

PD Dr. Dr. med. Frank Bloos
Klinik für Anästhesiologie und Intensivmedizin
Universitätsklinikum Jena
Am Klinikum 1, 07747 Jena
Tel: [redacted]
E-Mail: [redacted]

Dr. med. Martin Brauer
Klinik für Anästhesiologie und Intensivmedizin
Universitätsklinikum Jena
Am Klinikum 1, 07747 Jena
Tel: [redacted]
E-Mail: [redacted]

Dr. med. Carsten Herzog
Klinik für Anästhesiologie und Intensivmedizin
Universitätsklinikum Jena
Am Klinikum 1, 07747 Jena
Tel: [redacted]
E-Mail: [redacted]

Dr. med. Michael Hofmann
Klinik für Anästhesiologie und Intensivmedizin
Universitätsklinikum Jena
Am Klinikum 1, 07747 Jena
Tel: [redacted]
E-Mail: [redacted]

Dr. med. Hendrik Rüddel
Klinik für Anästhesiologie und Intensivmedizin
Universitätsklinikum Jena
Am Klinikum 1, 07747 Jena
Tel: [redacted]
E-Mail: [redacted]

Dr. med. Ingo Salzmann
Klinik für Anästhesiologie und Intensivmedizin
Universitätsklinikum Jena
Am Klinikum 1, 07747 Jena
Tel: [redacted]
E-Mail: [redacted]

Dr. med. Mark Simon
Klinik für Anästhesiologie und Intensivmedizin
Universitätsklinikum Jena
Am Klinikum 1, 07747 Jena
Tel: [redacted]
E-Mail: [redacted]

Dr. med. Helga Skupin
Klinik für Anästhesiologie und Intensivmedizin
Universitätsklinikum Jena
Am Klinikum 1, 07747 Jena
Tel: [redacted]
E-Mail: [redacted]

weitere Studienärzte (Oberärzte der Intensivstation, fortgesetzt)

Dr. med. Daniel Thomas-Rüddel
Klinik für Anästhesiologie und Intensivmedizin
Universitätsklinikum Jena
Am Klinikum 1, 07747 Jena
Tel: [redacted]
E-Mail: [redacted]

PD Dr. med. Christian von Löffelholz
Klinik für Anästhesiologie und Intensivmedizin
Universitätsklinikum Jena
Am Klinikum 1, 07747 Jena
Tel: 03641-9[redacted]
E-Mail: [redacted]

Dr. med. Isabella Westermann
Klinik für Anästhesiologie und Intensivmedizin
Universitätsklinikum Jena
Am Klinikum 1, 07747 Jena
Tel: [redacted]
E-Mail: [redacted]

Prof. Dr. med. Johannes Winning
Klinik für Anästhesiologie und Intensivmedizin
Universitätsklinikum Jena
Am Klinikum 1, 07747 Jena
Tel: [redacted]
E-Mail: [redacted]

Datenmanagerin

Cornelia Eichhorn
Zentrum für Klinische Studien
Universitätsklinikum Jena
Salvador-Allende-Platz 27, 07747 Jena
Tel.: 03641-9[redacted]
E-Mail: [redacted]@med.uni-jena.de

Erstvotierende Ethik-Kommission

Ethik-Kommission der Friedrich-Schiller-Universität Jena
Postfach
Bachstraße 18, 07740 Jena
Tel.: 03641-933[redacted]
E-Mail: ethikkommission@med.uni-jena.de

Kooperationspartner

Prof. Dr. Michael Bauer
Center for Sepsis Control and Care (CSCC)
Klinik für Anästhesiologie und Intensivmedizin
Universitätsklinikum Jena
Am Klinikum 1, 07747 Jena
Tel: 03641-9323100
E-Mail: michael.bauer@med.uni-jena.de

PD Dr. Christian Kosan
Zentrum für Molekulare Biomedizin
Friedrich-Schiller-Universität Jena
Hans-Knöll-Str. 2
07745 Jena
Tel: 03641-9[redacted]
E-Mail: christian.kosan@uni-jena.de

Prof. Dr. Andreas Stallmach
Klinik für Innere Medizin IV
- Gastroenterologie, Hepatologie,
Infektiologie, Interdisziplinäre Endoskopie -
Universitätsklinikum Jena
Am Klinikum 1
07747 Jena
Tel: 03641-9324400
E-Mail: Andreas.Stallmach@med.uni-jena.de

PD Dr. Dr. Michael Kiehntopf
Institut für Klinische Chemie und
Laboratoriumsdiagnostik
Universitätsklinikum Jena
Am Klinikum 1, 07747 Jena
Tel: 03641-9325 000
E-Mail: michael.kiehntopf@med.uni-jena

Prof. Dr. André Scherag
Center for Sepsis Control and Care (CSCC)
Klinische Epidemiologie
Universitätsklinikum Jena
Salvador-Allende-Platz 27, 07747 Jena
Tel: 03641-9[redacted]
E-Mail: andre.scherag@med.uni-jena

1.2 Synopse

**Titel (Deutsch)**	Identifikation kardiovaskulärer und molekularer Prognosefaktoren für die Morbidität und Mortalität bei COVID-19-Sepsis
**Titel (Engl.)**	Identification of cardiovascular and molecular prognostic factors for the morbidity and mortality in COVID-19-sepsis
**Kurzbezeichnung (Akronym)**	ICROVID
**Population / Indikation**	▪ volljährige Patienten mit COVID-19-assoziierter Sepsis und Indikation zur Intensivtherapie<br>o *mit Vorliegen einer septischen Kardiomyopathie*<br>o *ohne Vorliegen einer septischen Kardiomyopathie*<br><br>▪ volljährige Patienten mit Influenza-assoziierter Sepsis und Indikation zur Intensivtherapie<br><br>Als Kontrollkollektive dienen Patienten aus der ICROS Studie:<br>▪ volljährige Patienten mit intensivstationär behandelter Sepsis<br>▪ volljährige, gesunde Probanden (G)
**Design**	Prospektive, multizentrische Kohortenstudie
**Ziele**	▪ Umfassende Charakterisierung des akuten, postakuten, mittel- und langfristigen Patientenstatus mit Fokus auf kardiovaskuläre und metabolische Veränderungen bei COVID-19-Sepsis<br>▪ Identifikation potentieller Biomarker und theragnostischer Zielstrukturen/-moleküle sowie kardiovaskulärer, klinisch-epidemiologischer und laborchemischer Prognosefaktoren für die kurz-/mittel/langfristige Morbidität und Mortalität bei COVID-19-Sepsis
**Visiten**	T_0: Screening/Einschluss<br>T_1: 3 ± 1 d<br>T_2: 7 ± 1 d<br>T_3: 14 ± 1 d oder (bis zu 3 d vor) Krankenhaus-Entlassung<br><br>Nachbeobachtungsphase:<br>(Telefoninterview und Fragebogen)<br>T_4: nach 28 d<br>T_5: nach 90 d<br>T_6: nach 180 d<br>nach Erstdiagnose Sepsis
**Zielgrößen**	**Primärer Endpunkte**<br>▪ Mortalitätsunterschiede zwischen COVID-19-Sepsis-Patienten mit oder ohne Vorliegen einer septischen Kardiomyopathie zum Zeitpunkt 3 Monate nach Erstdiagnose COVID-19-Sepsis (T_5)<br><br>**Sekundäre Endpunkte**<br>▪ Mortalitätsunterschiede zwischen COVID-19-Sepsis-Patienten mit oder ohne Vorliegen einer septischen Kardiomyopathie zum Zeitpunkt 6 Monate nach Erstdiagnose COVID-19-Sepsis (T_5)<br>▪ Inzidenz kardiovaskulärer Ereignisse bei Patienten mit COVID-19-Sepsis im akuten (T_1, T_2), post-akuten (T_3-T_4) und Langzeitverlauf (T_5-T_6)

	▪ Unterschiede in der Inzidenz der septischen Kardiomyopathie im akuten Krankheitsverlauf (T_1, T_2) bei Patienten mit COVID-19-assoziierter Sepsis und Patienten mit Influenza-assoziierter Sepsis<br>▪ Unterschiede in der Inzidenz kardiovaskulärer Ereignisse bei Patienten mit COVID-19-assoziierter Sepsis und Patienten mit Influenza-assoziierter Sepsis im akuten (T_1, T_2), post-akuten (T_3-T_4) und Langzeitverlauf (T_5-T_6)<br>**Weitere klinische Fragestellungen** s. Abschnitt 4
**Anzahl Studienteilnehmer**	Patienten mit COVID-19-assoziierter Sepsis: bis 160<br>Patienten mit Influenza-assoziierter Sepsis: bis 160
**Einschlusskriterien**	**Patienten mit COVID-19-assoziierter und Influenza-assoziierter Sepsis**<br>▪ Alter ≥ 18 Jahre<br>▪ schriftliche Einwilligungserklärung des Patienten oder dessen gesetzlichen Vertreters vorliegend<br>▪ nachgewiesene SARS-CoV-2 oder nachgewiesene Influenzavirus-Infektion<br>▪ respiratorische Symptome<br>▪ Indikation zur Intensivtherapie<br>▪ Sepsis oder septischer Schock gemäß Sepsis-3 Kriterien<br>▪ Sepsisbeginn (infektionsbedingte SOFA-Score Änderung ≥ 2) nicht älter als 4 d (erste Blutentnahme innerhalb von 4 d nach Sepsisbeginn)
**Ausschlusskriterien**	**Patienten mit COVID-19-assoziierter und Influenza-assoziierter Sepsis**<br>▪ kardiochirurgischer Eingriff ≤ 12 Monate<br>▪ signifikante kardiale Erkrankung<br>o Endokarditis<br>o höhergradige Herzklappenvitien (schweres/Grad-3-Klappenvitium, symptomatische Aortenklappenstenose, mittelgradige Mitralklappeninsuffizienz mit eingeschränkter Ejektionsfraktion oder klinischer Symptomatik)<br>o komplexe strukturelle angeborene Herzerkrankung (z.B. TGA, Fallot-Tetralogie, Endokardkissendefekte etc.)<br>o hämodynamisch relevantes Shuntvitium<br>o vorbestehende, signifikante Einschränkungen der Herzleistung (Ejektionsfraktion < 45 % bzw. 10 % unter Normwert)<br>o vorbestehender pulmonaler Hypertonus<br>o Z. n. Myokardinfarkt (≤ 1 Jahr)<br>o Z. n. Herztransplantation<br>▪ kardiopulmonale Reanimation innerhalb der letzten 4 Wochen vor Sepsisbeginn<br>▪ Z. n. Pneumektomie<br>▪ Leberzirrhose Child C<br>▪ Kontraindikation für TEE (z. B. Ösophagusresektion, höhergradige Ösophagusvarizen) und unzureichende Schallbedingungen für TTE<br>▪ vorbestehende chronische terminale Niereninsuffizienz mit Dialyse<br>▪ Sepsis innerhalb der letzten 8 Monate<br>▪ Schwangerschaft/Stillzeit<br>▪ Therapiebeschränkung oder –einstellung<br>▪ Lebenserwartung ≤ 6 Monate aufgrund von Nebenerkrankungen<br>▪ vorherige Teilnahme an dieser Studie
**Zeitplan**	**Prüfungsbezogene Studiendauer**<br>Laufzeit Rekrutierung: 18 Monate<br>Einschluss erster Patient: erstes Quartal 2021<br>Einschluss letzter Patient: zweites Quartal 2022<br>Letztes Follow-Up: viertes Quartal 2022

	Zwischenauswertung	keine geplant
	Auswertung	nach Vorliegen der kompletten Datensätze 6 Monate nach Erstdiagnose COVID-19-Sepsis bzw. Influenza-Sepsis
	**Gesamtstudiendauer**	36 Monate
**Statistische Methoden**	Der primäre Endpunkt soll mithilfe einer Cox-Regression analysiert werden, wobei primär der Gruppenvergleich des Vorhandenseins einer septischen Kardiomyopathie auf die Überlebenszeit interessiert. Zur Beantwortung der sekundären Endpunkte und weiteren Fragestellungen wird auf adäquate statistische Standardverfahren zurückgegriffen. In den deskriptiven Analysen werden alle Parameter entsprechend ihres Skalenniveaus berichtet (relative und absolute Häufigkeiten, Lage- und Streuungsmaße). Gruppenvergleiche werden in Abhängigkeit der Verteilungseigenschaften der Zielparameter mit adäquaten Verfahren analysiert. Die Identifikation der Prognosefaktoren bzw. Prädiktoren trägt primär explorativen Charakter. Hierbei wird auf entsprechende Methoden der multivariaten Statistik, insbesondere Korrelations- und Regressionsanalysen, zurückgegriffen.	
**Finanzierung**	Bundesministerium für Bildung und Forschung, Projektförderung: Coldewey – ICROVID: Identifikation kardiovaskulärer und molekularer Prognosefaktoren für die Morbidität und Mortalität bei COVID-19-Sepsis (FKZ 03COV07) und Klinik für Anästhesiologie und Intensivmedizin des UKJ	

1.3 Ablauf- und Visitenplan

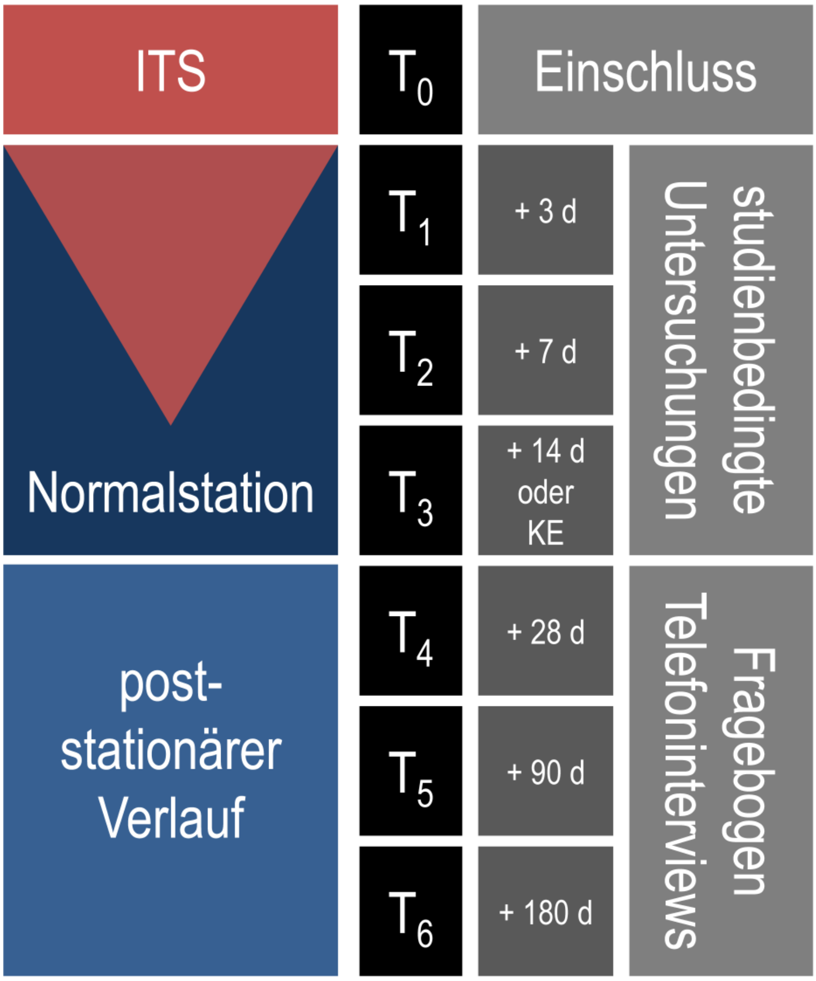

Abbildung 1. Zusammenfassende Darstellung der Visiten über den Behandlungsverlauf. Die studienbedingten Untersuchungen (z.B. Echokardiographie und Blutentnahmen) finden in der Akut- bzw. postakuten Erkrankungsphase statt und enden mit T_3 (14 Tage nach Erstdiagnose der COVID-19/Influenza-assoziierten Sepsis bzw. Krankenhausentlassung, KE). Die weiteren Erhebungen erfolgen im Rahmen von Telefoninterviews bzw. Fragebogenuntersuchungen.

Tabelle 1. Visitenplan.

Erhebungszeitpunkt	Screening	T_0	T_1	T_2	T_3	T_4	T_5	T_6
**Einschluss**								
Einwilligungserklärung	X							
Ein-/Ausschlusskriterien	X							
**COVID-19-Sepsis assoziierte Variablen**								
SIRS-Kriterien		X	X	X	X			
SOFA-Score		X	X	X	X			
APACHE II und SAPS II		X						
COVID-19 spezifische Variablen		X	X	X	X			
CAM-ICU		X	X	X	X			
**Vorgeschichte**								
Demographie/ Demographische Zusatzinformationen		X						
Komorbiditäten: Charlson Comorbidity Index		X						
Komorbiditäten: Kardiovaskuläre Risikofaktoren		X						
Komorbiditäten: Kardiovaskuläre Vorerkrankungen		X						
kardiologische Vorbefunde und Hausmedikation		X						
Anamnese und Patientenhistorie		X				X		
**apparative Untersuchungen**								
Echokardiographie (*TEE/TTE*)			X	X				
transiente Elastographie[1]			X	X				
erweitertes hämodynamisches Monitoring[2]			X	X	X			
Mikrobiom[1]				X				
**Blutuntersuchungen**								
Routinelabor		X	X	X	X			
studienbedingtes Labor[3]			X	X	X			
**Behandlungsverlauf/-daten**								
Infektion		X	X	X	X		X	X
Mikrobiologie		X	X	X	X			
kardiovaskuläre Ereignisse während Krankenhausbehandlung		X	X	X	X	X		
Krankenhausbehandlungs-/Entlassungsdaten						X		
physiologische Parameter		X	X	X	X			
Begleitmedikation		X	X	X	X		X	X
**Krankheitsverlauf**								
Überlebensstatus			X	X	X	X	X	X
Verlaufskrankengeschichte							X	X
kardiovaskuläre Ereignisse nach KH-Entlassung							X	X
Lebensqualität (EQ-5D-3L)							X	X
Assessment Langzeitfolgen (Fragebogen/Telefoninterview)							X	X

T_0: Screening/Einschluss
T_1: 3 ± 1 d nach Sepsisbeginn
T_2: 7 ± 1 d nach Sepsisbeginn
T_3: 14 ± 1 d nach Sepsisbeginn oder (bis zu 3 d vor) Krankenhaus-Entlassung
T_4: nach 28 d nach Sepsisbeginn
T_5: nach 90 d nach Sepsisbeginn
T_6: nach 180 d nach Sepsisbeginn

[1] optionale Durchführung in einzelnen Studienzentren (u.a. Universitätsklinikum Jena)
[2] ein erweitertes hämodynamisches Monitoring (z. B. PiCCO/PAK) erfolgt nur bei einem Teil der Patienten als Routinediagnostik und nicht als studienbedingte Maßnahme.
[3] in Blut und Urin zur Analyse von u.a. Surrogatparametern für kardiale Dysfunktion und weitere Organdysfunktionen, Surrogatparametern für Störung der endothelialen Barriere/Glykokalix sowie Analyse des Immunstatus, Metaboloms, Lipidoms

2 Abkürzungsverzeichnis

**28S rRNA**	ribosomale Ribonukleinsäure
**Abb.**	Abbildung
**ADL**	acitivities of daily living
**ANV**	akutes Nierenversagen
**APACHE II**	Acute Physiology and Chronic Health Evaluation II
**ARDS**	acute respiratory distress syndrome
**BB**	Blutbild
**BGA**	Blutgasanalyse
**BMBF**	Bundesministerium für Bildung und Forschung
**BNP**	B-type natriuretic peptide
**COVID-19**	coronavirus disease 2019
**CRF**	Case Report Form
**CSCC**	Center for Sepsis Control and Care
**d**	Tag/e
**DIC**	disseminierte intravaskuläre Koagulation
**dL**	Deziliter
**e-CRF**	electronic case report form
**EHEC**	enterohämorrhagische Escherichia coli
**EKG**	Elektrokardiographie
**et al.**	et alia
**GCP**	Good Clinical Practice
**GCS**	Glasgow Coma Scale
**h**	Stunde/n
**https**	HyperText Transfer Protocol Secure
**IADL**	instrumental acitivities of daily living
**ICMJE**	International Committee of Medical Journal Editors
**ICROS**	Akronym der Studie „Identifikation kardiovaskulärer und molekularer Prognosefaktoren für die mittel- und langfristige Morbidität und Mortalität bei Sepsis“
**IL**	Interleukin
**INTERMACS**	Interagency Registry for Mechanically Assisted Circulatory Support
**ISF**	Investigator Site File (Studienzentrumsordner)
**ITS**	Intensivstation
**M**	Monat/e
**max.**	maximal
**mg**	Milligramm
**min**	Minute
**mmHg**	Millimeter Quecksilbersäule
**NSP1**	Nichtstrukturprotein 1
**NWG**	Nachwuchsforschungsgruppe
**PAK**	Pulmonalarterienkatheter
**PI**	Principal investigator
**PiCCO**	Pulse Contour Cardiac Output
**PTBS**	Posttraumatische Belastungsstörung
**s**	Sekunden

**SAPS II**	Simplified Acute Physiology Score
**SARS-CoV-2**	severe acute respiratory syndrome coronavirus 2
**SIRS**	Systemisches inflammatorisches Response-Syndrom
**SOFA**	sequential organ failure assessment
**STROBE**	Strengthening the reporting of observational studies in epidemiology
**Stx**	Shiga-Toxin
**T**	Time - Zeitpunkt
**TEE**	Transösophageale Echokardiographie
**Tel.**	Telefon
**t-MoCa**	Montreal Cognitive Assessment (telephone)
**TRIPOD**	Transparent reporting of a multivariable prediction model for individual prognosis or diagnosis
**TTE**	Transthorakale Echokardiographie
**UKJ**	Universitätsklinikum Jena
**W**	Woche/n
**WHO**	Weltgesundheitsorganisation
**ZIK**	Zentrum für Innovationskompetenz
**ZKS**	Zentrum für klinische Studien

3 Ausgangssituation und Fragestellung

3.1 Sepsis

Bei einer Sepsis kommt es durch eine dysregulierte Wirtsantwort in Folge einer Infektion zur Funktionseinschränkung oder zum Versagen von einem oder mehreren Organsystemen [1]. Verursacht werden kann die zugrunde liegende Infektion durch eine Vielzahl von Krankheitserregern: hauptsächlich Bakterien und in geringerem Maße Viren, Pilze sowie Parasiten [2]. Die höchste Inzidenz und Mortalität der Sepsis wird in den Gebieten mit geringstem sozio-demographischen Index verzeichnet [3]. Aber auch in Ländern mit einem hohen Lebensstandard ist die Sepsis eine häufig unterschätzte Erkrankung. Von ursächlicher Bedeutung ist hier auch die zunehmende Möglichkeit der Anwendung invasiver Verfahren, wie großer Operationen oder immunmodulierender Chemotherapien, die bei Patienten häufig zu einer beeinträchtigten Immunfunktion führen [4]. Ein ganz aktuelles Beispiel für die große gesellschaftliche, ökonomische und politische Bedeutung der Sepsis ist die derzeitige Pandemie. Ausgelöst wurde sie durch einen Ausbruch von Infektionen mit dem neuen Beta-Coronavirus SARS-CoV-2 (*severe acute respiratory syndrome coronavirus 2*) in der chinesischen Stadt Wuhan im Dezember 2019 [5]. Die durch dieses Virus ausgelöste Erkrankung wurde durch die Weltgesundheitsorganisation WHO als COVID-19 (*coronavirus disease 2019*) bezeichnet. Binnen kürzester Zeit hat die COVID-19-Pandemie Gesundheitssysteme weltweit an ihre Versorgungsgrenzen gebracht.

Trotz ausgiebiger Forschungsarbeiten der letzten Jahrzehnte führte bisher keine klinische Studie zur Implementation einer kausalen Therapie der Sepsis in die klinische Praxis. Wissenschaftliche Ergebnisse sprechen dafür, dass Patienten mit Sepsis gemäß ihres klinischen und molekularen Phänotyps und Risikoprofils stratifiziert und einer personalisierten Therapie zugeführt werden sollten [6]. Ein solches personalisiertes Vorgehen kommt im Bereich der Krebstherapie schon lange zur Anwendung [7]. Patienten mit Sepsis bilden ein sehr heterogenes Patientenkollektiv [6]. Daher erscheint es besonders wichtig, dass in klinischen Studien die Unterschiedlichkeit der Patienten hinsichtlich der individuellen Krankengeschichte, der klinischen Präsentation, der individuellen molekularen und immunologischen Wirtsantwort und des krankheitsursächlichen Pathogens Berücksichtigung findet. Innovative translationale Forschungsansätze sind erforderlich, um neue Zielstrukturen zu identifizieren, anhand derer bei dieser schweren systemischen Erkrankung diagnostische und therapeutische Strategien entwickelt werden können [8]. Gute erste Ansätze liefert eine Studie von Seymour *et al.*, in der anhand klinischer Kriterien Sepsispatienten in 4 klinische Phänotypen mit unterschiedlichen Risikoprofilen eingeteilt werden konnten [9]. Auch Patienten mit einer COVID-19-Sepsis scheinen einen spezifischen Phänotyp zu entwickeln, den es durch klinische und molekulare Untersuchungen weiter zu charakterisieren gilt.

3.2 COVID-19-Sepsis

Mittlerweile ist bekannt, dass die Schwere der Krankheitsverläufe nach SARS-CoV-2-Infektion interindividuell stark variiert, ohne die ursächlichen molekularen Mechanismen zu kennen. Zu den typischen COVID-19-Symptomen zählen neben Fieber und trockenem Husten [10] auch ein Geschmacks- und Geruchsverlust [11]. Weltweit wurden neben symptomlosen Infektionen und milden Verläufen von COVID-19 auch schwere Verläufe mit der Entwicklung einer Pneumonie mit ausgeprägter Hypoxie, einem akuten Lungenversagen (ARDS) oder einem Multiorganversagen mit oder ohne Todesfolge beobachtet. Die WHO berichtete abschließend über den initialen Ausbruch in China, dass etwa 80 % der labordiagnostisch bestätigten SARS-CoV-2-positiven Patienten milde bis moderate Erkrankungsverläufe zeigen, während es bei 13,8 % zu schweren und bei 6,1 % zu kritischen Verläufen kommt [5]. Auch in Deutschland entwickeln etwa 5 % der Patienten infolge einer SARS-CoV-2-Infektion lebensbedrohliche Organdysfunktionen, die eine Intensivtherapie erforderlich machen (www.divi.de/register/tagesreport). Diese sehr schweren Verläufe der COVID-19-Erkrankung kann man definitionsgemäß unter dem Oberbegriff Sepsis subsummieren [1]. Einhergehend mit einer ausgeprägten Reaktion des Immunsystems (sog. Zytokinsturm) kommt es bei diesen Patienten zu der oben erwähnten ausgeprägten Lungenschädigung mit schwerer Oxygenierungsstörung. Oftmals benötigen sie eine invasive Beatmung, aber auch

andere Organdysfunktionen, wie etwa Nieren- und Leberversagen müssen behandelt werden. Damit kann die Gruppe der Sepsispatienten mit COVID-19 als ein spezieller Phänotyp der Sepsis angesehen werden. Mehrere Autoren berichten zudem über eine hohe Inzidenz kardialer Komplikationen [12], das Auftreten eines ausgeprägten Endothelschadens [13] und Gerinnungsstörungen mit einer Häufung von lebensbedrohlichen thromboembolischen Ereignissen [12, 14, 15]. Nach aktuellen Zahlen des DIVI IntensivRegisters und des Robert-Koch-Instituts versterben etwa 25 % der intensivmedizinisch betreuten COVID-19-Patienten (www.divi.de/register/tagesreport). Inwiefern die Mortalität und die langfristige Morbidität durch das Auftreten einer sog. septischen Kardiomyopathie beeinflusst werden, ist unbekannt. Aufgrund der Neuheit der Erkrankung liegt noch keine abschließende Beschreibung aller Symptome und deren Häufigkeiten vor. Der Wissensstand hinsichtlich dieses Krankheitsbildes ändert sich derzeit täglich.

3.3 Kardiovaskuläre Komplikationen bei COVID-19

Viele Autoren beschreiben, dass Patienten mit einer Virusgrippe ein höheres Risiko haben, kardiovaskuläre Komplikationen zu erleiden [16, 17]. Eine Influenza mit primär pulmonaler Organbeteiligung ist häufig mit einer bakteriellen Superinfektion vergesellschaftet und führt nicht selten zu einer Sepsis [18]. Extrapulmonale Organdysfunktionen, wie eine Beteiligung des Herzens durch die Entwicklung einer Myokarditis oder Kardiomyopathie, sind beschrieben [19]. Auch bei einer COVID-19-Sepsis kommt es gehäuft zu kardiovaskulären Komplikationen. Gegen SARS-CoV-2 können sich Risikogruppen jedoch, im Gegensatz zu Influenzaviren, in Ermangelung eines geeigneten und verfügbaren Impfstoffs nicht schützen. Die molekularen Mechanismen und die Bedeutung der kardiovaskulären Ereignisse für den mittel- und langfristigen Krankheitsverlauf der Patienten mit COVID-19-Sepsis sind bisher nicht aufgeklärt.

Zu den bei Patienten mit COVID-19 beschriebenen kardiovaskulären Komplikationen zählen Herzinfarkte, Herzrhythmusstörungen, Arrhythmien, Herzbeuteltamponade und thromboembolische Ereignisse wie beispielsweise Lungenembolien (Übersicht in [20]). Eine myokardiale Schädigung, nachgewiesen durch einen Anstieg des Herzenzyms Troponin, tritt bei ca. 20 % der kritisch kranken COVID-19-Patienten auf [21-23]. In einer monozentrischen Studie war eine laborchemisch festgestellte kardiale Schädigung mit einer signifikant erhöhten Mortalität assoziiert [22]. In dieser prospektiven Studie war der Endpunkt jedoch variabel (Nachbeobachtung Tag 1 bis 37), was eine genaue Abschätzung der Mortalität erschwert. Außerdem wurde keine Bildgebung des Herzens durchgeführt, so dass ein Zusammenhang zwischen den laborchemischen Veränderungen und der Herzfunktion nicht untersucht wurde. Thromboembolische Ereignisse bei intensivpflichtigen COVID-19 Patienten wurden in Studien bisher mit einer Häufigkeit von etwa 17–30 % beobachtet [24-26] und haben die Notwendigkeit einer ausreichenden Hemmung der Blutgerinnung herausgestellt [27]. Auch funktionelle Einschränkungen der Pumpfunktion des Herzens sind mit 12 % beschrieben [10]. Die Ursachen hierfür sind nicht geklärt. Eine kardiale Dekompensation einer bereits bestehenden Herzerkrankung oder eine durch das Virus hervorgerufene Herzmuskelentzündung ist denkbar. In Autopsien an 39 Verstorbenen mit COVID-19 konnten in 41 % signifikante Mengen an SARS-CoV-2 Virus im Herzgewebe nachgewiesen werden [28]. Da keine zelluläre Entzündungsreaktion festgestellt werden konnte, bleibt das Vorhandensein einer Myokarditis in diesen Patienten unklar. Zuletzt kann auch die Sepsis, welche die Patienten entwickeln, eine septische Kardiomyopathie bedingen, welche ebenfalls mit einer Einschränkung der Pumpfunktion einhergeht [29].

3.4 Mikroangiopathische Komplikationen von COVID-19

Thrombotische Mikroangiopathien können eine intensivmedizinische Therapie erforderlich machen. In Lungenautopsien von 7 COVID-19-Patienten konnte eine thrombotische Mikroangiopathie der Lungengefäße festgestellt werden [13]. Ferner zeigten diese Lungen spezifische Merkmale im Vergleich zu Lungen von Patienten mit Influenza, etwa eine 9mal häufiger auftretende Thrombose der alveolären Kapillaren. Eine weitere, meist intensivmedizinisch zu behandelnde Erkrankung, welche interessanterweise sowohl klinische als auch pathophysiologische Ähnlichkeiten zu COVID-19 aufweist, ist das hämolytisch-urämische Syndrom. Dieses entwickelt sich in 5–10 % der Fälle infolge einer Infektion mit enterohämorrhagischen *Escherichia coli* (EHEC) [30]. Pathognomonisch ist die klinische Trias aus mikroangiopathischer hämolytischer Anämie, Thrombozytopenie und akutem Nierenversagen. Ebenso wie bei der SARS-CoV-2-Infektion ist bisher nicht bekannt, warum nach einer

Infektion mit EHEC einige Patienten nur leichte Symptome entwickeln, andere aber lebensbedrohlich erkranken. Sowohl bei COVID-19 als auch beim hämolytisch-urämischen Syndrom können schwere Verläufe als eine definierte Sepsisentität angesehen werden. Als weitere Gemeinsamkeit kommt es auch beim hämolytisch-urämischen Syndrom zu thromboembolischen Komplikationen und in Fallberichten zu kardialen Komplikationen [31-34]. Diese Komplikationen reichen von linksventrikulärer Hypertrophie und eingeschränkter kardialer Funktion bis zu Perikardergüssen und chronischer Herzinsuffizienz [35]. Autopsien von 64 Patienten mit hämolytisch-urämischem Syndrom fanden in 19 Fällen kardiale Komplikationen, wobei in der Mehrzahl diese Komplikationen durch thrombotische Mikroangiopathie im Myokard ausgelöst wurde [36]. Das genaue Ausmaß kardiovaskulärer Komplikationen im hämolytisch-urämischen Syndrom und ihr Einfluss auf den Langzeitverlauf ist bisher nicht prospektiv untersucht, was auch dem sporadischen Auftreten der Erkrankung geschuldet ist.

Interessanterweise weisen die beiden Erkrankungen – COVID-19-Sepsis und hämolytisch-urämisches Syndrom – molekularbiologisch ähnliche Pathomechanismen auf. Die krankheitsursächlichen Erreger – SARS-CoV-2 und EHEC – besitzen Pathogenitätsfaktoren, welche die Proteinbiosynthese auf Ebene der Translation hemmen. Ein Hauptvirulenzfaktor von EHEC, das Shiga-Toxin (Stx) [37], bindet den Globotriaosylceramid-3-Rezeptor, welcher besonders stark von renalen mikrovaskulären Endothelzellen exprimiert wird, weshalb die Niere hauptsächlich betroffen ist [38]. Stx wird über Endozytose in die Zelle aufgenommen und führt dort zu einer Inhibition der Proteinbiosynthese [39] durch Deaktivierung der katalytisch aktiven 28S rRNA [40]. Das von SARS-CoV-2 exprimierte Protein Nsp1 hingegen bindet die ribosomale 40S-Untereinheit und blockiert damit die Proteinbiosynthese [41] Der auftretende Endothelzellschaden führt zu einem prothrombotischen Milieu, mikroangiopathischer Thrombosierung und der Aktivierung des Immunsystems [39]. Dabei treten pathophysiologische Parallelen zur disseminierten intravasalen Koagulopathie (DIC) während der Sepsis auf [42].

Ein wesentlicher Unterschied beider Erkrankungen besteht hinsichtlich ihres Organotropismus: Während es bei der durch SARS-CoV-2-verursachten COVID-19-Sepsis bevorzugt zu einer pulmonalen Pathologie kommt [43], interagiert das durch EHEC produzierte Shiga-Toxin überwiegend mit seinen in den Nieren und dem Endothel angereicherten Rezeptoren und führt zu einem akuten Nierenversagen [38]. Bei beiden Erkrankungen kommt es aber auch zu extrapulmonalen [44] bzw. extrarenalen [35] Organmanifestationen.

3.5 Fragestellungen und Ziele der Studie

Das vorliegende Projektvorhaben soll unter Verwendung klinischer Untersuchungsmethoden und modernster laborchemischer Analysetechniken das klinische und molekulare Verständnis schwerer COVID-19-Krankheitsverläufe einschließlich des mittel- und langfristigen Verlaufs der Erkrankung grundlegend und systematisch erweitern. Unter Berücksichtigung der aktuellen Gegebenheiten und Entwicklung der COVID-19-Pandemie, insbesondere der Infektionszahlen und Häufigkeit schwerer Verläufe, ist das Vorhaben als multizentrische prospektive klinische Studie angelegt.

Das primäre Studienziel ist die Überprüfung von Mortalitätsunterschieden bei Patienten mit COVID-19-Sepsis mit oder ohne Vorliegen einer septischen Kardiomyopathie 3 Monate nach Sepsisbeginn. Als Vergleichsgruppe werden Patienten mit Influenza- Sepsis prospektiv untersucht.

Des Weiteren soll der Krankheitsverlauf von Patienten mit COVID-19-Sepsis nachfolgend direkt und systematisch mit den bereits im Rahmen des Projektvorhabens der NWG Translational Septomics generierten Datensätzen von Sepsispatienten und gesunden Probanden verglichen werden (Kohortenstudie *„Identifikation kardiovaskulärer und molekularer Prognosefaktoren für die mittel- und langfristige Morbidität und Mortalität bei Sepsis“*, Akronym: ICROS, DRKS00013347, NCT03620409, [45]).

4 Studienziele und Endpunkte

Übergeordnetes Ziel dieser prospektiven multizentrischen Kohortenstudie auf deutschen Intensivstationen ist die Identifikation kardiovaskulärer und molekularer Prognosefaktoren für die mittelfristige Morbidität und Mortalität nach COVID-19-assoziierter Sepsis als Grundlage für die Entwicklung zielgerichteter personalisierter Strategien.

Um dieses Ziel zu erreichen, sind folgende Endpunkte definiert:

Primärer Endpunkte

- Mortalitätsunterschiede zwischen COVID-19-Sepsis-Patienten mit oder ohne Vorliegen einer septischen Kardiomyopathie zum Zeitpunkt 3 Monate nach Erstdiagnose COVID-19-Sepsis (T_5)

Sekundäre Endpunkte

- Mortalitätsunterschiede zwischen COVID-19-Sepsis-Patienten mit oder ohne Vorliegen einer septischen Kardiomyopathie zum Zeitpunkt 6 Monate nach Erstdiagnose COVID-19-Sepsis (T_5)
- Inzidenz kardiovaskulärer Ereignisse bei Patienten mit COVID-19-Sepsis im akuten (T_1, T_2), post-akuten (T_3-T_4) sowie Langzeitverlauf (T_5-T_6)
- Unterschiede in der Inzidenz der septischen Kardiomyopathie im akuten Krankheitsverlauf (T_1, T_2) bei Patienten mit COVID-19-Sepsis und Patienten mit Influenza-Sepsis
- Unterschiede in der Inzidenz kardiovaskulärer Ereignisse bei Patienten mit COVID-19-Sepsis und Patienten mit Influenza-Sepsis im akuten (T_1, T_2), post-akuten (T_3-T_4) sowie Langzeitverlauf (T_5-T_6)

Weitere klinische Fragestellungen *zu Beantwortung derer Patienten mit COVID-19-Sepsis und Patienten mit Influenza-Sepsis sowie vorbekannten Kohorten von Patienten mit nicht-COVID-19-assoziierter Sepsis und von gesunden Probanden verglichen werden. Hierfür werden u. a. folgende Zielgrößen analysiert: kardiovaskuläre Risikofaktoren und Funktion,* Steifigkeit der Leber/Nieren*, Organdysfunktionen, Immunstatus, Metabolom, Lipidom und allgemeines Funktionsniveau*:

- Häufigkeitsunterschiede bezüglich des Auftretens einer septischen Kardiomyopathie zwischen Patienten mit COVID-19-Sepsis und nicht-COVID-19-assoziierter Sepsis während des akuten Krankheitsverlaufs (kumulativ T_1 und T_2)
- Identifikation COVID-19-spezifischer klinischer und molekularer Veränderungen des akuten und postakuten Erkrankungsverlaufs
- Analyse potentieller Gruppenunterschiede des akuten und post-akuten Krankheitsverlaufs (Gesamtkollektiv und stratifiziert nach Vorliegen einer septischen Kardiomyopathie) zur Identifikation von Parametern mit potentiell diagnostischer Relevanz bei COVID-19-Sepsis
- Charakterisierung des akuten und post-akuten Krankheitsverlaufs bei Patienten mit COVID-19-Sepsis (Gesamtkollektiv und stratifiziert nach Vorliegen einer septischen Kardiomyopathie) zur Exploration potentieller Surrogatparameter für das Auftreten einer kardialen Dysfunktion
- Vergleich der Inzidenz des Rechtsherzversagens während des akuten Krankheitsverlaufs (T_1 und T_2) bei Patienten mit COVID-19-assoziierter Sepsis und Influenza-assoziierter Sepsis
- Einfluss des Rechtsherzversagens auf die Mortalität und Morbidität nach COVID-19-assoziierter bzw. Influenza-assoziierter Sepsis
- Analyse potentieller Gruppenunterschiede des mittel- (T_5) und langfristigen (T_6) Krankheitsverlaufs in Patienten mit COVID-19 Sepsis (Gesamtkollektiv und stratifiziert nach Vorliegen einer septischen Kardiomyopathie) und der Vergleichskohorten zur Identifikation potentieller Biomarker des akuten und postakuten Krankheitsverlauf und damit auch potentieller therapeutischen Zielstrukturen, die präklinisch weiter untersucht werden können.
- Ermittlung der mittel- und langfristigen Morbidität bei Patienten mit COVID-19-Sepsis hinsichtlich der Leistungsfähigkeit bzw. Belastungseinschränkung, Lebensqualität sowie Art und Häufigkeit kardiovaskulärer Ereignisse nach ITS-Einweisung (Gesamtkollektiv und stratifiziert nach Vorliegen einer septischen Kardiomyopathie)
- Identifizierung potentieller Prädiktoren für die mittel- (T_5) und langfristige (T_6) Mortalität und Morbidität nach COVID-19-Sepsis. Im Fokus der Analysen stehen die im akuten (T_1, T_2) und post-akuten (T_3) Behandlungsbereich gewonnenen Daten

Tabelle 2. Übersicht der Untersuchungen, Untersuchungsparameter und Operationalisierungen.

Domäne		Subdomäne	*Operationalisierung / konkrete Erhebungsmethode*
**1.**	**Screening**	Ein- und Ausschlusskriterien, Einwilligungserklärung	*Prüfung der Kriterien, Einholung der Einwilligung, Einschluss in die Studie, ggf. Einholung der nachträglichen Einwilligung bei nichteinwilligungsfähigen Patienten*
**2.**	**Demographie, anamnestische Informationen, Verlaufskrankengeschichte**	Demographie/ demographische Zusatzinformationen	*Alter, Geschlecht, Größe, Gewicht, Aufnahmedaten Krankenhaus/ITS, Zuweisungsart, Aufenthaltsort vor Aufnahme, vorausgegangene ITS-Behandlungen, vorbestehende Tracheostoma/Beatmungspflichtigkeit, Grad der Behinderung, Pflegestufe, Arbeitsunfähigkeit, Berentung.*
		Komorbiditäten	*Charlson Comorbidity Index*
		kardiovaskuläre Risikofaktoren	*u. a. arterielle Hypertonie, Diabetes (inkl. HbA1c), Dyslipoproteinämie (inkl. LDL-/HDL-/ Cholesterin und Triglyzeride),*
		kardiovaskuläre Vorerkrankungen und Ereignisse vor Diagnosestellung Sepsis	*Hausmedikation, kardiologische Vorbefunde (EKG, TTE/TEE), arterielle Hypertonie, koronare Herzerkrankung, Angina pectoris, Herzinfarkt, eingeschränkte Ejektionsfraktion, Herzinsuffizienz, Herzrhythmusstörungen, Herzklappenvitien, pAVK, cerebrovaskuläre Ereignisse*
		Operation	*im aktuellen Krankenhausaufenthalt*
**3.**	**Infektionsdaten**	Infektion	*Grad der Sicherung, Ursprung, Lokalisation*
		Kriterien der Sepsis / septischen Schock (alte und neue Kriterien)	*SIRS-Kriterien, Organdysfunktionen, septischer Schock*
		Mikrobiologie	*Erreger, Erreger der Sepsis, Resistenz*
		Sekundärinfektion	*Grad der Sicherung, Lokalisation*
**4.**	**klinische Scores**	SOFA	*Routinelabordiagnostik, mehrmalige Erhebung*
		APACHE II	*Routinelabordiagnostik, einmalige Erhebung*
		SAPS II	*Routinelabordiagnostik, einmalige Erhebung*
		CAM-ICU	*mehrmalige Erhebung*
		COVID-Hyperinflammations-Score [46]	*Labordiagnostik, Bildgebung, Fieber*
**5.**	**Routinelabor / Surrogatparameter Organdysfunktion**	kardiovaskuläres System	*proBNP, BNP, Troponin*
		renales System	*Kreatinin, Kreatinin-Clearances*
		hepatisches System	*ASAT, ALAT, AP, CHE, Gamma-GT, GLDH, Quick, Bilirubin, Albumin*
		respiratorisches System	*BGA, Laktat*
		Hämatologie	*kleines BB, großes BB*
		Inflammation	*CRP, PCT, Leukozyten, IL-6, Ferritin*
**6.**	**physiologische Parameter / Surrogatparameter Organdysfunktion**	physiologische Parameter	*Herzfrequenz, Blutdruck, Körpertemperatur, Atemfrequenz*
		respiratorisches System	*Beatmung, BGA*
		Neurologie	*Patientenstatus, GCS, Delir, Butyrylcholinesterase, Acetylcholinesterase POCT[1]*
		renales System	*Urinausscheidung, ANV, Nierenersatztherapie*
**7.**	**studienbedingte Laborparameter**	Metabolom und Lipidom	*massenspektrometrische Untersuchung von Steroiden, Acylcarnitinen, Aminosäuren und biogenen Aminen, Monosacchariden, Sphingolipiden, Glycerophospolipiden, Gallensäuren, Leukotrienen, Prostaglandinen*
		Transkriptom und Proteom	*u.a. Transkriptom-Analysen von peripheral blood mononuclear cells (PBMCs)*
		Störung der endothelialen Barriere	*Immunglobuline, Wachstumsfaktoren, Zelladhäsionsmoleküle, Trägerproteine der Blutgerinnung, Glycocalix-Marker, mitochondriale DNA*
		Immunstatus	*Zytokine, Chemokine, Immunphänotypisierung, Glykoproteomik, epigenetische Regulation von*

			Immunzellen
		Surrogatparameter Infektion	*PCT, CRP*
		Mikrobiom[1]	*Shotgun metagenomic sequencing*
**8.**	**klinische Untersuchungen**	kardiale Funktion	*TTE/TEE (physiologische Parameter, Dimension, Funktion, Hämodynamik)*
		Leber- und Nierensteifigkeit	*Fibroscan®[1]*
		Hämodynamisches Monitoring	*PiCCO/PAC (erfolgt nur im Rahmen der Routinediagnostik)*
**9.**	**Krankenhausbehandlungs-Entlassungsdaten / Begleitmedikation**	Sepsisbehandlung	*Dauer, Volumentherapie, Vasopressoren und Inotropika, Beatmung, Antibiose, sepsisspezifische Therapie*
		Krankenhausbehandlung	*Dauer, Zustand bei Entlassung, Überlebensstatus*
		Anamnese und Patientenhistorie	*Implantate, Nikotinverhalten, körperliche Aktivität vor KH-Aufnahme*
		Verlaufskrankengeschichte	*Aufenthaltsort*
		Patientenstatus	*Todesursache, Zeitpunkt KH-Entlassung, Entlassung nach, Gewicht, Beatmungstage, Tage mit Nierenersatzverfahren, Tage mit Vasopressoren, andere Organersatzverfahren, weitere Krankenhaus-/ITS-Behandlungen*
		Kardiovaskuläre Ereignisse nach Diagnosestellung Sepsis	*Kardiopulmonale Reanimationen, arterielle Hypertonie, koronare Herzerkrankung, Angina Pectoris, Herzinfarkt, Herzinsuffizienz, Herzrhythmusstörungen, Herzklappenvitien, pAVK, cerebrovaskuläre Ereignisse*
**10.**	**Langzeitfolgen und Lebensqualität**	Langzeitfolgen	*Telefoninterview/Fragebogen (Screeningfragen, ADL/IADL, Angst- und Depressionssymptomatik, Atemnot, Fatigue, PTBS, Schmerzgeschehen und Erhebung kognitiver Einschränkungen mithilfe des t-MoCa)*
		Lebensqualität	*EQ-5D-3L (retrospektiv und aktueller Status)*

[1] optionale Durchführung in einzelnen Studienzentren (u.a. Universitätsklinikum Jena)

5 Studienpopulation

Die primären Studienpopulationen bestehen aus intensivmedizinisch versorgten, volljährigen Patienten mit einer COVID-19-Sepsis (nach Sepsis-3-Kriterien) sowie einer Gruppe intensivmedizinisch versorgten, volljährigen Patienten mit Influenza-Sepsis (nach Sepsis-3-Kriterien). Im Rahmen des multizentrischen Studienvorhabens ist der Einschluss von je 160 Patienten pro Gruppe geplant.

5.1 Einschlusskriterien

- Alter ≥ 18 Jahre
- schriftliche Einwilligungserklärung des Patienten oder dessen gesetzlichen Vertreters vorliegend
- nachgewiesene SARS-CoV-2 Infektion oder nachgewiesene Influenzavirus-Infektion
- respiratorische Symptome
- Indikation zur ITS-Therapie
- Sepsis oder septischer Schock gemäß Sepsis-3 Kriterien
- erste Infektionsbezogene Organdysfunktion (= Diagnose Sepsis) nicht älter als 4 Tage (erste Blutentnahme innerhalb von 4 Tagen nach Sepsisbeginn)

5.2 Ausschlusskriterien

- kardiochirurgischer Eingriff ≤ 12 Monate
- signifikante kardiale Erkrankung
  - Endokarditis
  - höhergradige Herzklappenvitien (schweres/Grad-3-Klappenvitium, symptomatische Aortenklappenstenose, mittelgradige Mitralklappen-insuffizienz mit eingeschränkter Ejektionsfraktion oder klinischer Symptomatik)
  - komplexe strukturelle angeborene Herzerkrankung (z.B. TGA, Fallot-Tetralogie, Endokardkissendefekte etc.)
  - hämodynamisch relevantes Shuntvitium
  - vorbestehende, signifikante Einschränkungen der Herzleistung (Ejektionsfraktion < 45 % bzw. 10 % unter Normwert)
  - vorbestehender pulmonaler Hypertonus
  - Z. n. Myokardinfarkt (≤ 1 Jahr)
  - Z. n. Herztransplantation
- kardiopulmonale Reanimation innerhalb der letzten 4 Wochen vor Sepsisbeginn
- Z. n. Pneumektomie
- Leberzirrhose Child C
- Kontraindikation für TEE (z. B. Ösophagusresektion, höhergradige Ösophagusvarizen) und unzureichende Schallbedingungen für TTE
- vorbestehende chronische terminale Niereninsuffizienz mit Dialyse
- Sepsis innerhalb der letzten 8 Monate
- Schwangerschaft/Stillzeit
- Therapiebeschränkung oder –einstellung
- Lebenserwartung ≤ 6 Monate aufgrund von Nebenerkrankungen
- vorherige Teilnahme an dieser Studie

5.3 Definition der Kriterien Sepsis/septischer Schock (Sepsis-3-Kriterien)

Diagnosekriterien für Sepsis und septischen Schock [1]

I. Nachweis der Infektion

Diagnose einer Infektion über den mikrobiologischen Nachweis oder durch klinische Kriterien.

II. Organdysfunktion

Akute Veränderung im SOFA Score ≥ 2 Punkte infolge einer Infektion (siehe Anhang).

III. Septischer Schock

Persistierende Hypotonie mit Vasopressoreinsatz, um mittleren arteriellen Blutdruck ≥ 65 mmHg zu halten und Serum-Laktat > 2 mmol/l (18 mg/dl). Die Hypotonie besteht trotz adäquater Volumengabe.

5.4 Definition der Kriterien septische Kardiomyopathie

In dieser Studie wird die septische Kardiomyopathie definiert als eine systolische Dysfunktion bestimmt anhand einer reduzierten linksventrikulären Ejektionsfraktion gemäß der Empfehlungen der *American Society of Echocardiography* und der *European Association of Cardiovascular Imaging* (< 52 % bei Männern und < 54 % bei Frauen) zu T_1 und/oder T_2 bei einem Patienten mit Sepsis [47-51]) oder – bei vorbestehender leichtgradig eingeschränkter Ejektionsfraktion – einer Reduktion des Ausgangswertes zum Zeitpunkt T_1 und/oder T_2 um mindestens 10 %. Da keine verbindlichen Kriterien einer septischen Kardiomyopathie existieren, kann diese Definition entsprechend neuer Erkenntnisse modifiziert werden.

6 Studienablauf

Das Screening und der Einschluss von intensivpflichtigen Patienten mit COVID-19-Sepsis bzw. Influenza-assoziierter Sepsis erfolgen auf den Intensivstationen der beteiligten Studienzentren. Einige der studienbedingten Untersuchungen, insbesondere zu den postakuten Visiten T_2 und T_3, werden auf den Normalstationen der beteiligten Studienzentren stattfinden. Die Follow-Up-Visiten (T_4— T_6) erfolgen im Rahmen von Telefoninterviews bzw. Fragebogenuntersuchungen.

6.1 Sepsis-Patienten (COVID-19 und Influenza)

6.1.1 Screening und Patientenidentifikationsliste

Patienten, die auf einer der Intensivstationen der beteiligten Studienzentren behandelt werden, werden täglich hinsichtlich o.g. Kriterien für das Vorliegen der Diagnose COVID-19-Sepsis bzw. Influenza-assoziierter Sepsis untersucht. Patienten, die die Einschlusskriterien erfüllen, werden im Screening Log gelistet, Patienten, die in die Studie eingeschlossen wurden, werden in der Patientenidentifikationsliste.

6.1.2 Aufklärung und Einwilligung

Die Studienteilnahme ist freiwillig. Die Aufklärung der Patienten erfolgt mit der vorgegebenen Informationsschrift sowie durch ein Gespräch mit einem Arzt des Studienteams. Die Einwilligung erfolgt schriftlich auf dem vorgegebenen Formular. Die Einwilligungserklärung muss in zweifacher Ausfertigung vorliegen. Ein Exemplar verbleibt im Studienzentrum und ist für mind. 10 Jahre nach Studienende im Studienzentrumsordner (Investigator Site File – ISF) aufzubewahren. Das zweite Exemplar wird dem Einwilligenden zusammen mit der Patienteninformation ausgehändigt.

6.1.2.1 Einwilligungsfähige Patienten

Einwilligungsfähige Patienten müssen vor Beginn der Studie mündlich und schriftlich über die Ziele, die Dauer, den Ablauf, den Nutzen und sämtliche Risiken der Studie aufgeklärt werden. Der aufklärende Arzt überzeugt sich davon, dass die Aufklärung vom Patienten verstanden wurde. Nach der Aufklärung erhält jeder Patient ausreichend Zeit und Gelegenheit, offene Fragen zu klären und über seine Teilnahme zu entscheiden. Jeder Patient unterzeichnet und datiert seine Einwilligung in die Teilnahme an der Studie eigenhändig schriftlich auf der Einwilligungserklärung. Ist ein einwilligungsfähiger Patient nicht in der Lage, eigenhändig die Einwilligung zu unterzeichnen, muss ein Zeuge, welcher nicht Mitglied des Studienteams sein darf, während des Aufklärungsprozesses anwesend sein. Dieser Zeuge bestätigt die mündliche Aufklärung und Einwilligung des Patienten durch Datum und Unterschrift.

6.1.2.2 Nicht-einwilligungsfähige Patienten

Aufgrund der Schwere der Erkrankung ist davon auszugehen, dass es sich bei den einzuschließenden Studienpatienten größtenteils um nicht-einwilligungsfähige Patienten handelt. In diesem Fall ist das Einholen der Einwilligungserklärung des Patienten vor Beginn der Datenerhebung für die Studie nicht möglich. Aus diesem Grund muss die schriftliche Einwilligung eines gesetzlichen Betreuers des Patienten bzw. des Bevollmächtigten eingeholt werden, um eine Studienteilnahme zu ermöglichen. Primär nicht-einwilligungsfähige Patienten werden, sofern sie im Verlauf einwilligungsfähig sind, nachträglich mündlich und schriftlich über die Teilnahme an der Studie informiert und um Einwilligung gebeten. Bei nicht-einwilligungsfähigen Patienten ohne Bevollmächtigten oder gesetzlichen Betreuer erfolgt eine Reevaluation der Einwilligungsfähigkeit bzw. die Bestellung eines gesetzlichen Betreuers. Sollte die Einwilligung zur Studienteilnahme (persönlich oder durch einen Bevollmächtigter/gesetzlichen Betreuer) nicht möglich sein, kann ein unabhängiger Arzt im Sinne einer Konsiliararzt-Regelung über die Studienteilnahme entscheiden. Sobald ein Bevollmächtigter erreichbar ist oder ein gesetzlicher Vertreter bestimmt

wurde, so wird dessen Einwilligung zur Studienteilnahme schnellstmöglich eingeholt. Sollte der Patient seine Einwilligungsfähigkeit erlangen, so wird dessen Einwilligung zur Studienteilnahme eingeholt.

6.1.2.3 Keine oder Rücknahme der Einwilligung

Patienten ohne Einwilligung oder Zustimmung eines Konsiliararztes werden nicht in die Studie eingeschlossen. Aufgrund des längsschnittlichen Designs ist der Einschluss zu einem späteren Zeitpunkt nicht möglich. Der Patient bzw. sein Bevollmächtigter oder sein gesetzlicher Betreuer können jederzeit und ohne Angabe von Gründen die Einwilligung zurückziehen und die Studie abbrechen. Der Zeitpunkt der Rücknahme der Einwilligung wird dokumentiert. Außerdem wird der Patient gefragt, ob ggf. bereits erhobene Studiendaten im Rahmen der Studienauswertung weiter verwendet werden dürfen. Sollte der Patient der Weiterverwendung der Daten und Proben nicht zustimmen, werden die Proben zum Studienende vernichtet und alle bereits erhobene Patientendaten gehen nicht in die Auswertung ein.

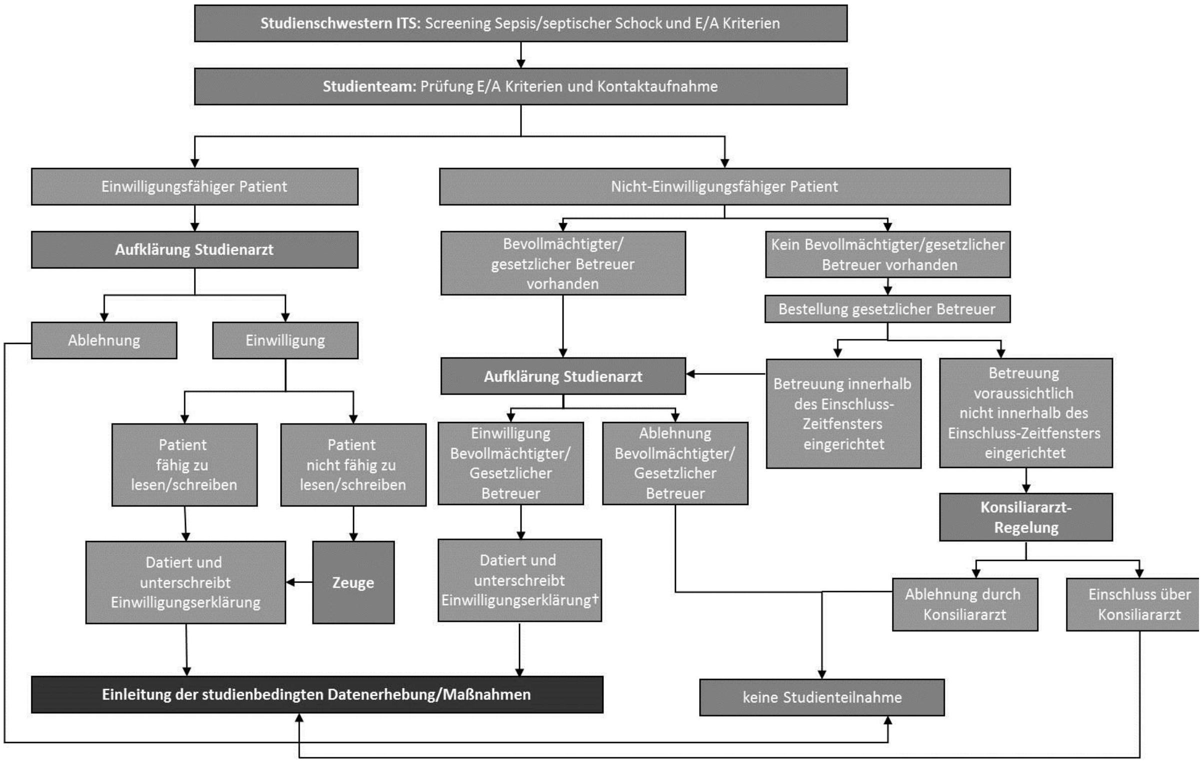

Abbildung 2. Flowchart der Aufklärung und Einwilligungserklärung bei COVID-19-Sepsis-Patienten bzw. Patienten mit Influenza-assoziierter Sepsis.

† primär nicht-einwilligungsfähige Patienten werden, sofern sie im Verlauf einwilligungsfähig sind, nachträglich mündlich und schriftlich über die Teilnahme an der Studie informiert und um Einwilligung gebeten.

6.1.3 Dokumentation im Studienverlauf

Es sind die folgenden Erhebungszeitpunkte geplant: 3 ± 1 d (T_1), 7 ± 1 d (T_2) sowie 14 ± 1 d (T_3) nach Erstdiagnose COVID-19-assoziieter Sepsis bzw. Influenza-assoziierter Sepsis. Sollte die Krankenhausentlassung des Patienten früher als 14 d geplant sein, wird der Zeitpunkt T_3 bis zu 3 Tagen vor der Entlassung erfolgen. Die Follow-Up-Visiten erfolgen im Rahmen von Telefoninterviews und Fragebögen. Die Visiteninhalte sind unter **Abschnitt 1.3** sowie **Tabelle 2** in **Abschnitt 4** aufgelistet. Datenpunkte aus Telefoninterviews und Fragebögen können als Einzelfallentscheidung auch retrospektiv zu einem späteren Zeitpunkt erfasst werden.

6.1.4 Studienbedingte Maßnahmen

Studienbedingte Laborabnahmen erfolgen zu den Visiten T_1, T_2 und T_3. Die echokardiographischen Untersuchungen sowie die optionale transiente Elastographie erfolgen zu den Visiten T_1 und T_2. Die Telefoninterviews bzw. Fragebogenerhebungen erfolgen zu den Visiten T_4 bis T_6.

6.1.5 Studienende / Ende der Nachbeobachtung

Das reguläre Studienende für jeden Patienten ist der Abschluss der letzten Follow-Up-Visite oder das Versterben auf der ITS/Krankenhaus bzw. nach Krankenhausentlassung. Durch die Rücknahme der Einwilligungserklärung kann die Studienteilnahme eines Patienten vorzeitig abgebrochen werden. Kann der Kontakt zu dem Patienten nach der Entlassung aus dem Krankenhaus nicht mehr hergestellt werden, ist die Studie für den Patienten ebenfalls vorzeitig beendet („lost to follow-up").

6.2 Auswertungsphase

Nach Abschluss der Datenerhebung erfolgt eine Analyse- und Publikationsphase. Nach Beantwortung aller offenen Queries und Abschluss des Datenmanagements wird die Datenbank geschlossen und zur Auswertung übergeben.

7 Beschreibung der Methoden

7.1 Klinische Untersuchungen

7.1.1 TEE/TTE

Die Echokardiographie ist gegenwärtig das bedeutendste Untersuchungsverfahren für die Beurteilung von Herzstruktur und Funktion, dass bettseitig durchgeführt werden kann. Die zu untersuchenden Parameter sind detailliert im CRF aufgeführt. Bei den Patienten in dieser Studie ist eine solche Beurteilung der Herzfunktion im Rahmen der Grunderkrankung regelhaft indiziert und stellt dann keine studienbedingte Maßnahme dar. Standardmäßig wird eine TTE durchgeführt, die mit keinen relevanten Risiken verbunden ist. Nur bei unzureichenden transthorakalen Schallbedingungen, z. B. durch Ödembildung im Rahmen der Erkrankung, wird eine TEE durchgeführt. Die TEE kann sehr selten zu Verletzungen der Speiseröhre, des Kehlkopfs oder der Zähne führen. Das zur Rachenbetäubung verwendete Lokalanästhetikum kann eine allergische Reaktion auslösen. Durch die Rachenbetäubung kann es zum Verschlucken von Flüssigkeit oder Speiseresten in die Luftröhre kommen. Bei Einsatz eines Beruhigungsmittels sind Unverträglichkeitsreaktionen oder eine Beeinträchtigung der Atmung möglich. Selten kommt es zu Herzrhythmusstörungen. Die verwendeten Sonden werden entsprechend der gesetzlichen Richtlinien und den Vorgaben unseres Instituts für Hygiene gereinigt. Eine Übertragung von Krankheitserregern ist daher sehr unwahrscheinlich. Bei den TTE und TEE Untersuchungen wird ein einfaches EKG mit drei Elektroden angelegt. In seltenen Fällen kann es zu Hautreaktion im Bereich der Klebeelektroden kommen.

7.1.2 Transiente Elastographie

Bei der „transienten Elastographie“ (syn. Fibroscan®) handelt es sich um ein ultraschallbasiertes, nicht-invasives Verfahren, um die Steifigkeit der Leber abzuschätzen, die bei Lebererkrankungen gut mit dem Grad der Fibrose korreliert. Die Untersuchung wird als etabliertes Verfahren bei Patienten mit chronischen Lebererkrankungen, wie zum Beispiel einer nicht-alkoholischen Fettleber, zur Verlaufsbeurteilung eingesetzt. Im Rahmen von septischen Erkrankungen treten häufig Schädigungen der Leber auf, diese werden aktuell vor allem mittels laborchemischer Parameter, wie dem Bilirubin und den Transaminasen erfasst. Im Rahmen der aktuellen Studie soll daher zusätzlich untersucht werden, ob eine Messung mittels transienter Elastographie bei Patienten mit Sepsis ein geeignetes Instrument ist, um eine Schädigung der Leber zu erkennen, oder ob es bei diesen Patienten nicht zuverlässig eingesetzt werden kann.

Die Untersuchung erfolgt bettseitig und dauert pro Messung in etwa fünf Minuten. Die Untersuchung ist, da es sich um ein ultraschallbasiertes Verfahren handelt, für die Patienten mit keinem erhöhten Risiko verbunden und stellt auch keine Belastung für die Patienten dar.

7.2 Laboruntersuchungen

7.2.1 Erhebung von Routineparametern

Im Rahmen der täglichen Laborabnahmen auf ITS und Normalstation werden regelmäßig Infektionsparameter, Laborwerte zur Beurteilung der Organfunktionen und ein Blutbild der letzten 24 Stunden dokumentiert. Dieses sind keine studienbedingten Maßnahmen. Dies gilt auch für die Visiten, die potentiell auf Normalstation stattfinden.

7.2.2 Studienbedingte Untersuchungen

Für die laborchemischen Analysen ist die Entnahme von ca. 60 ml Blut geplant. Die Blutabnahme erfolgt durch geschultes Fachpersonal und entsprechend dem festgelegten Probenabnahmeschema. Falls vorhanden werden bereits angelegte Katheter benutzt. Etwaige Nebenwirkungen bei Blutentnahmen sind Blutungen, Infektionen, Nervenläsionen oder Thrombosen.

7.3 Gesundheitsbezogene Lebensqualität und Langzeitfolgen

In **Tabelle 3** sind die Inhalte der Telefoninterviews bzw. die Fragebogenverfahren zusammengefasst.

Tabelle 3. Zusammenfassung der Untersuchungen am Patienten.

Untersuchung	Dauer
**klinische Untersuchungen**	
TEE/TTE	45 min
transiente Elastographie[1]	5 min
**Laboruntersuchungen**	
Blut- und Urin Entnahme	5 min
**Telefoninterviews**	
Anamnese/Verlaufskrankengeschichte	10 min
kardiovaskuläre Ereignisse seit Krankenhausentlassung	5 min
kognitive Leistungsfähigkeit: t-MoCa [52]	10 min
**Fragebogen**	
Screeningfragen (z. B. Riech- und Geschmacksstörungen)	5 min
Aktivitäten des alltäglichen Lebens (ADL): Barthel Index [53]	5 min
Angst- und Depressionssymptomatik: BSI-18 [54]	5 min
Fatigue: Fatigue Skala [55]	5 min
instrumentelle Aktivitäten des alltäglichen Lebens (IADL) [56]	5 min
Lebensqualität: EQ-5D-3L [57]	5 min
Symptome posttraumatische Belastungsstörung: PTSS-14 [58]	5 min
Schmerzgeschehen: u.a. Korff Graded Chronic Pain Scale [59]	5 min
Gesamtdauer Fragebogen:	*40 min*

[1] *optionale Durchführung in einzelnen Studienzentren (u.a. Universitätsklinikum Jena)*

8 Unerwünschte Ereignisse

Unerwünschte Ereignisse werden nicht erwartet.

9 Datenmanagement und Qualitätssicherung

9.1 Patientenidentifikationsliste

Alle patientenbezogenen Daten werden in pseudonymisierter Form erfasst. Dazu wird ein nicht-sprechendes Pseudonym verwendet, aus welchem allein nicht auf die Identität des Patienten geschlossen werden kann.
Die Studienzentren führen eine Patientenidentifikationsliste, in der die Patientenidentifikationsnummern mit den vollen Patientennamen der Teilnehmer und Geburtsdatum verbunden sind. Diese muss im Studienordner abgelegt werden. Die Liste dient der Möglichkeit der späteren Identifikation teilnehmender Personen. Sie ist absolut vertraulich zu behandeln und darf das Erhebungszentrum nicht verlassen. Sie ist nach Studienende **mindestens zehn Jahre** zu archivieren. Zusätzlich wird die Studienteilnahme oder der geplante Einschluss in die Studie in der Patientenakte vermerkt.

9.2 Liste der Verantwortlichkeiten

Es muss sichergestellt werden, dass jede Person, die für die Dokumentation im eCRF verantwortlich ist, identifiziert werden kann. Eine Liste mit Unterschrift und Kürzel der Personen, die Eintragungen im eCRF vornehmen dürfen (Signature/Delegation Log), wird im ISF und im TMF abgelegt. Mit dieser Übersicht werden auch weitere Personen, die in an der Studie beteiligt sind, mit ihren Namen, Unterschrift und Kürzel sowie ihren Verantwortlichkeiten und

Befugnissen benannt.

9.3 Datenerhebung/Dokumentationsbögen

Zur Erreichung des Studienzieles ist es erforderlich, medizinische Daten einzelner Patienten zu erheben und zu verarbeiten. Die für die klinische Studie relevanten Daten werden per RDE (Remote Data Entry – Elektronische Dateneingabe) erfasst. Dazu werden die Daten von einem autorisierten Mitglied des Studienzentrums an einem online geschalteten Arbeitsplatzrechner in spezielle Masken eingegeben, die ein elektronisches CRF darstellen. Über das elektronische CRF werden die Daten direkt in die Studiendatenbank im ZKS Jena übernommen. Es liegt in der Verantwortung des Leiters des jeweiligen Studienzentrums, dass alle im Rahmen der klinischen Prüfung erhobenen Daten korrekt und vollständig in die speziell für diese klinische Prüfung erstellte Datenbank eingetragen werden. Korrekturen im eCRF dürfen nur von autorisierten Personen vorgenommen werden und sind zu begründen.

Ein papierbasiertes CRF als „Ansichtsexemplar" wird den Studienzentren als Bestandteil des ISF ausgehändigt. Die entsprechenden Mitarbeiter erhalten eine Anleitung zum Ausfüllen der elektronischen Dokumente.

9.4 Datenverarbeitung

Die Datenerfassung dient wissenschaftlichen Zwecken. Die Daten werden im Studienzentrum erzeugt. Alle gesammelten medizinischen Daten werden von den entsprechenden Mitarbeitern in einem computer-basierten Online-Dateneingabe-System eingegeben und sofort auf die Server am Zentrum für Klinische Studien Jena übertragen. Die Datenerfassung erfolgt via Webapplikation auf den Servern des ZKS des Universitätsklinikums Jena in die Studienmanagement-Software „OpenClinica®". Die Software erfüllt die regulatorischen Anforderungen (GCP, 21 CFR Part 11). Die Daten werden über eine verschlüsselte Datenverbindung (HTTPS) in Eingabemasken per Webbrowser erfasst. Um eine pseudonymisierte Datenanalyse zu gewährleisten, wird eine eindeutige Patientenidentifikationsnummer jedem Patienten zugeordnet.

Für das Datenmanagement wird ebenfalls die Studienmanagementsoftware "OpenClinica®" verwendet. Die Überprüfung der Richtigkeit der Daten erfolgt durch Range-, Validitäts- und Konsistenzchecks. Nicht plausible oder fehlende Daten werden im Studienzentrum nachgefragt. Jede Änderung an den Daten, z. B. aufgrund der Einarbeitung von beantworteten Rückfragen, wird über eine automatische Änderungsverfolgung (Audittrail) in der Datenbank dokumentiert. Durch den Einsatz eines hierarchischen, auf Rollen basierenden Zugriffskonzeptes ist ein unberechtigter Zugriff auf die Studiendaten unmöglich.

9.5 Aufbewahrung der Studienunterlagen

Das Zentrum für Klinische Studien am Universitätsklinikum Jena ist als Dokumentationszentrum auch für die Datenspeicherung ernannt. Die Sicherung von elektronischen Daten geschieht regelmäßig. Die Datenspeichereinrichtungen sind in einem verschlossenen, zentralen Raum, zu dem nur Systemadministratoren Zutritt haben.

Der Studienleiter muss sicherstellen, dass die wichtigsten Unterlagen für mindestens 10 Jahre nach Beendigung der Studie aufbewahrt werden. Andere Vorschriften für die Aufbewahrung von medizinischen Unterlagen bleiben unberührt. Alle Unterlagen müssen an einem sicheren Ort aufbewahrt und vertraulich behandelt werden. Falls erforderlich (z. B. aufgrund gesetzlicher Bestimmungen oder nach Rücksprache mit dem Studienleiter) können die Unterlagen über den oben genannten Zeitraum hinaus aufgehoben werden. Aufzeichnungen und Dokumente im Zusammenhang mit der Studie, z. B. die Patientenidentifikationsliste, Einwilligungserklärungen, Korrespondenz mit der Ethik-Kommission, den zuständigen Behörden, der Studienleitung und andere relevante Dokumente, müssen mindestens 10 Jahre in den Studienzentren aufbewahrt werden (oder länger, falls gesetzlich vorgeschrieben). Das Studienzentrum muss Vorkehrungen treffen, um eine versehentliche oder vorzeitige Zerstörung dieser Dokumente zu verhindern.

9.6 Datenschutz

Im Rahmen der Studie ist es erforderlich, von den Studienteilnehmern personenbezogene Daten (z. B. vollständiger

Name, Initialen des Vor- und Zunamens, Geburtsdatum, Adresse) und Daten zur Behandlung und zum Krankheitsverlauf (z. B. medizinische Befunde, Behandlungsarten, verordnete Medikamente) zu erheben und zu verarbeiten. Diese Daten werden im Studienzentrum erhoben und in pseudonymisierter Form (d. h. ohne direkten Bezug zum Patientennamen) mit Hilfe einer Patientenidentifikationsnummer elektronisch gespeichert, an die verantwortliche datenverarbeitende Stelle übermittelt und ausgewertet.

Im Falle eines Widerrufs der Einwilligung zur Studie durch den Patienten einschließlich der weiteren Datenerhebung werden ab dem Widerrufszeitpunkt keine weiteren Daten erhoben. Die bisher erhobenen Daten werden innerhalb der Studie nur bei erteilter Erlaubnis durch den ehemaligen Studienteilnehmer weiter verwendet und ausgewertet.

10 Biometrie

10.1 Endpunkte

Der primäre Endpunkt sind Mortalitätsunterschiede zwischen COVID-19-Sepsis-Patienten mit oder ohne Vorliegen einer septischen Kardiomyopathie zum Zeitpunkt 3 Monate nach Erstdiagnose COVID-19-Sepsis (T_5). Die sekundären Endpunkte und weitere Fragestellungen sind **Abschnitt 4** zu entnehmen.

10.2 Definition von Auswertungskollektiven

Für Patienten mit COVID-19-Sepsis ergeben sich drei Auswertungskollektive in der Akutphase (T_1, T_2), Post-Akutphase (T_3, T_4) sowie im mittel- (T_5) und langfristigen (T_6) Verlauf:

- Gesamtkollektiv
- COVID-19-Sepsis-Patienten mit septischer Kardiomyopathie
- COVID-19-Sepsis-Patienten ohne septische Kardiomyopathie

Für Patienten mit Influenza-Sepsis ergeben sich drei Auswertungskollektive in der Akutphase (T_1, T_2), Post-Akutphase (T_3, T_4) sowie im mittel- (T_5) und langfristigen (T_6) Verlauf:

- Gesamtkollektiv
- Influenza-Sepsis-Patienten mit septischer Kardiomyopathie
- Influenza-Sepsis-Patienten ohne septische Kardiomyopathie

Für die Kontrollgruppen aus der ICROS Studie ergeben sich folgende Auswertungskollektive:

Patienten mit Sepsis bzw. septischen Schock nach Sepsis-3-Definition:

- Gesamtkollektiv
- Sepsis-Patienten mit septischer Kardiomyopathie
- Sepsis-Patienten ohne septische Kardiomyopathie

Gesunde Probanden mit Fokus auf den Einschlusszeitpunkt (T_1)

10.3 Planung des Studienumfanges (Fallzahlplanung)

Die Fallzahlplanung basiert auf ähnlichen Abwägungen wie bei der ICROS-Studie [45]. Der Fokus der Planung liegt auf Mortalitätsunterschieden nach 3 Monaten. Wird ein einfacher $\chi 2$-Test bei einem zweiseitigen Signifikanzniveau von $\alpha = 5$ % angewendet, so ist eine Kohortengröße von n = 80 Patienten pro Gruppe ausreichend, um Unterschiede (absolute Risikoreduktion) in der 3-Monats-Mortaliät von ≥ 22 % mit einer statistischen Power von ≥ 80 % zu detektieren, wenn eine 3 Monats-Mortalität von auf Intensivstation behandelten COVID-19-Patienten ohne Kardiomyopathie von 50 % angenommen wird (ähnlich der Mortalität bei septischem Schock). Für den Fall einer geringeren 3-Monats-Mortaliät in der Gruppe von intensivmedizinisch behandelten COVID-19-Patienten ohne Kardiomyopathie von z. B. 40 % lassen sich ähnlich große Unterschiede (absolute Risikoreduktion) ebenfalls mit einer Power ≥ 80 % nachweisen. Die Fallzahlüberlegungen erfolgten mittels der Funktion power.prop.test in R (Version 4.0.2).

10.4 Zwischen-/Auswertung

Es ist keine Zwischenauswertung des kompletten Datensatzes geplant. Die Endauswertung erfolgt nach Vorliegen der kompletten Datensätze 6 Monate nach Erstdiagnose COVID-19-Sepsis. Zur Beantwortung spezifischer Fragestellungen werden gegebenenfalls eingeschränkte Datensätze ausgelesen.

10.5 Weitere statistische Analysen

Zur Beantwortung der sekundären Endpunkte und weiteren Fragestellungen wird auf adäquate statistische Standardverfahren zurückgegriffen. In den deskriptiven Analysen werden alle Parameter entsprechend ihres Skalenniveaus berichtet (relative und absolute Häufigkeiten, Lage- und Streuungsmaße). Gruppenvergleiche werden in Abhängigkeit der Verteilungseigenschaften der Zielparameter mit adäquaten Verfahren analysiert. Die Identifikation der Prognosefaktoren bzw. Prädiktoren trägt primär explorativen Charakter. Hierbei wird auf entsprechende Methoden der multivariaten Statistik, insbesondere Korrelations- und Regressionsanalysen, zurückgegriffen.

10.6 Präsentation der Ergebnisse

Die Ergebnispräsentation erfolgt anhand der STROBE-Kriterien für die Darstellung von Beobachtungsstudien [60] sowie der TRIPOD Kriterien [61] für prognostische Fragestellungen.

11 Publikation / Nutzung der Ergebnisse / Registrierung der Datenerhebung

11.1 Abschlussbericht und Publikationen

11.1.1 Publikation des Studienprotokolls

Das finale, konsentierte Studienprotokoll wird gemäß den Gepflogenheiten internationaler Studiengruppen und den unten aufgeführten Regularien in einem Journal mit Peer Review publiziert.

11.1.2 Abschlussbericht

Zwischen- und Abschlussberichte werden entsprechend der Vorgaben des Bundesministeriums für Bildung und Forschung erstellt.

11.1.3 Analysen und Publikationen

Analysen und Publikationen zu primären und sekundären Fragestellungen werden von den Studienleitern, wissenschaftlichen Mitarbeitern und einem unabhängigen bioinformatischen bzw. statistischen Dienstleister vorgenommen. Örtliche Studienleiter behalten das Anrecht, auf die eigenen Daten zuzugreifen und diese uneingeschränkt zu nutzen und zu publizieren.

11.2 Zitierweise

Jede Veröffentlichung ist mit folgendem Zusatz zu versehen:
„Diese Studie wurde durch das Bundesministerium für Bildung und Forschung gefördert (Coldewey – ICROVID: Identifikation kardiovaskulärer und molekularer Prognosefaktoren für die Morbidität und Mortalität bei COVID-19-Sepsis, FKZ 03COV07)“

”This study was funded by the Federal Ministry of Education and Research (Coldewey – ICROVID: Identification of cardiovascular and molecular prognostic factors for the morbidity and mortality in COVID-19-sepsis, grant 03COV07)“

11.3 Autoren

Bezüglich der Rechte und Pflichten der beteiligten Autoren sind die Publikationsrichtlinien für Autoren in medizinischen Fachzeitschriften gemäß den Empfehlungen der ICMJE (http://www.icmje.org/ethical_1author.html) maßgeblich. Die letzte Entscheidung über Autorenschaft und Reihenfolge der Autoren obliegt dem Studienleiter. Mindestens die Studienleitung kann geltend machen, als Ko-Autor einer Publikation zu weitergehenden Fragestellungen genannt zu werden, vorausgesetzt, sie hat einen relevanten Beitrag zu dieser Publikation geleistet.

11.4 Registrierung

Die vorliegende Studie wird vor Einschluss des ersten Studienpatienten im Deutschen Register Klinischer Studien sowie im Register Clinical Trials.gov registriert.

12 Ethische Belange und administrative Regelungen

12.1 Deklaration von Helsinki und Gute Klinische Praxis

Die Studie wird gemäß den ethischen Grundsätzen durchgeführt, die ihren Ursprung in der Deklaration von Helsinki haben. Die jeweils aktuelle Version der Deklaration wird beachtet. Die Empfehlungen der Guten Klinischen Praxis, gültig seit dem 17.1.1997, sofern zutreffend, werden berücksichtigt.

12.2 Ethik-Kommissionen

Das Studienprotokoll wird mit den erforderlichen weiteren Unterlagen der Ethik-Kommission des Universitätsklinikum Jena mit der Bitte um Bewertung vorgelegt. Die Studie kann erst nach zustimmender Bewertung der Ethik-Kommission beginnen. Äquivalent wird in allen beteiligten Studienzentren verfahren.

12.3 Nachträgliche Änderungen

Das Studienprotokoll ist einzuhalten. Jede vom Studienleiter zu vertretende Abweichung von den vorgesehenen Untersuchungsmaßnahmen oder -zeitpunkten ist zu dokumentieren und zu begründen.
Änderungen oder Ergänzungen des Studienprotokolls können nur von der Studienleitung veranlasst und autorisiert werden. Über Änderungen des Studienprotokolls wird die erstvotierende Ethik-Kommission informiert. Ggf. wird erneut die zustimmende Bewertung eingeholt. Bewertungspflichtige Änderungen dürfen nicht vor der Entscheidung der Ethik-Kommission umgesetzt werden.
Änderungen der von der Ethik-Kommission zustimmend bewerteten Studie, die geeignet sind,

- sich auf die Sicherheit der betroffenen Personen auszuwirken,
- zusätzliche Datenerhebungen oder Auswertungen, die eine Änderung der Patienteninformation und/oder -einwilligung erfordern,
- die Auslegung der wissenschaftlichen Dokumente, auf die die Studie gestützt wird, oder die wissenschaftliche Aussagekraft der Studienergebnisse zu beeinflussen,
- die Art der Leitung oder Durchführung der Studie wesentlich zu verändern,
- dürfen nur vorgenommen werden, wenn diese Änderungen von der Ethik-Kommission zustimmend bewertet wurden.

12.4 Finanzierung

Die Finanzierung des Projekts erfolgt durch das Bundesministerium für Bildung und Forschung (Coldewey – ICROVID: Identifikation kardiovaskulärer und molekularer Prognosefaktoren für die Morbidität und Mortalität bei COVID-19-Sepsis, FKZ 03COV07) und wird durch die Klinik für Anästhesiologie und Intensivmedizin des UKJ unterstützt.

13 Literatur

1. Singer, M., et al., *The Third International Consensus Definitions for Sepsis and Septic Shock (Sepsis-3).* JAMA, 2016. **315**(8): p. 801-10.
2. Zahar, J.R., et al., *Outcomes in severe sepsis and patients with septic shock: pathogen species and infection sites are not associated with mortality.* Crit Care Med, 2011. **39**(8): p. 1886-95.
3. Rudd, K.E., et al., *Global, regional, and national sepsis incidence and mortality, 1990-2017: analysis for the Global Burden of Disease Study.* Lancet, 2020. **395**(10219): p. 200-211.
4. Cohen, J., et al., *Sepsis: a roadmap for future research.* Lancet Infectious Diseases, 2015. **15**(5): p. 581-614.
5. WHO, *Report of the WHO-China Joint Mission on Coronavirus Disease 2019 (COVID-19).* 2020.
6. Cohen, J., et al., *Sepsis: a roadmap for future research.* Lancet Infect Dis, 2015. **15**(5): p. 581-614.
7. Wang, R.F. and H.Y. Wang, *Immune targets and neoantigens for cancer immunotherapy and precision medicine.* Cell Res, 2017. **27**(1): p. 11-37.
8. Lewis, A.J., J.S. Lee, and M.R. Rosengart, *Translational Sepsis Research: Spanning the Divide.* Crit Care Med, 2018. **46**(9): p. 1497-1505.
9. Seymour, C.W., et al., *Derivation, Validation, and Potential Treatment Implications of Novel Clinical Phenotypes for Sepsis.* JAMA, 2019. **321**(20): p. 2003-2017.
10. Huang, C., et al., *Clinical features of patients infected with 2019 novel coronavirus in Wuhan, China.* Lancet, 2020. **395**(10223): p. 497-506.
11. Hornuss, D., et al., *Anosmia in COVID-19 patients.* Clin Microbiol Infect, 2020.
12. Guzik, T.J., et al., *COVID-19 and the cardiovascular system: implications for risk assessment, diagnosis, and treatment options.* Cardiovasc Res, 2020.
13. Ackermann, M., et al., *Pulmonary Vascular Endothelialitis, Thrombosis, and Angiogenesis in Covid-19.* N Engl J Med, 2020. **383**(2): p. 120-128.
14. Michael Henry, B., et al., *Hyperinflammation and Derangement of Renin-Angiotensin-Aldosterone System in COVID-19: a novel hypothesis for clinically suspected hypercoagulopathy and microvascular immunothrombosis.* Clin Chim Acta, 2020.
15. Connors, J.M. and J.H. Levy, *COVID-19 and its implications for thrombosis and anticoagulation.* Blood, 2020.
16. Warren-Gash, C., L. Smeeth, and A.C. Hayward, *Influenza as a trigger for acute myocardial infarction or death from cardiovascular disease: a systematic review.* Lancet Infect Dis, 2009. **9**(10): p. 601-10.
17. Kindermann, I., et al., *Predictors of outcome in patients with suspected myocarditis.* Circulation, 2008. **118**(6): p. 639-48.
18. Morens, D.M., J.K. Taubenberger, and A.S. Fauci, *Predominant role of bacterial pneumonia as a cause of death in pandemic influenza: implications for pandemic influenza preparedness.* J Infect Dis, 2008. **198**(7): p. 962-70.
19. Sellers, S.A., et al., *The hidden burden of influenza: A review of the extra-pulmonary complications of influenza infection.* Influenza Other Respir Viruses, 2017. **11**(5): p. 372-393.
20. Hendren, N.S., et al., *Description and Proposed Management of the Acute COVID-19 Cardiovascular Syndrome.* Circulation, 2020. **141**(23): p. 1903-1914.
21. Yang, X., et al., *Clinical course and outcomes of critically ill patients with SARS-CoV-2 pneumonia in Wuhan, China: a single-centered, retrospective, observational study.* Lancet Respir Med, 2020. **8**(5): p. 475-481.
22. Shi, S., et al., *Association of Cardiac Injury With Mortality in Hospitalized Patients With COVID-19 in Wuhan, China.* JAMA Cardiol, 2020.
23. Lippi, G., C.J. Lavie, and F. Sanchis-Gomar, *Cardiac troponin I in patients with coronavirus disease 2019 (COVID-19): Evidence from a meta-analysis.* Prog Cardiovasc Dis, 2020.
24. Bilaloglu, S., et al., *Thrombosis in Hospitalized Patients With COVID-19 in a New York City Health System.* JAMA, 2020.
25. Klok, F.A., et al., *Incidence of thrombotic complications in critically ill ICU patients with COVID-19.* Thromb Res, 2020. **191**: p. 145-147.
26. Middeldorp, S., et al., *Incidence of venous thromboembolism in hospitalized patients with COVID-19.* J

Thromb Haemost, 2020.
27. Tang, N., et al., *Anticoagulant treatment is associated with decreased mortality in severe coronavirus disease 2019 patients with coagulopathy.* J Thromb Haemost, 2020. **18**(5): p. 1094-1099.
28. Lindner, D., et al., *Association of Cardiac Infection With SARS-CoV-2 in Confirmed COVID-19 Autopsy Cases.* JAMA Cardiol, 2020.
29. Sato, R. and M. Nasu, *A review of sepsis-induced cardiomyopathy.* J Intensive Care, 2015. **3**: p. 48.
30. Fakhouri, F., et al., *Haemolytic uraemic syndrome.* Lancet, 2017. **390**(10095): p. 681-696.
31. Matthies, J., et al., *Extrarenal Manifestations in Shigatoxin-associated Haemolytic Uremic Syndrome.* Klin Padiatr, 2016. **228**(4): p. 181-8.
32. Askiti, V., et al., *Troponin I levels in a hemolytic uremic syndrome patient with severe cardiac failure.* Pediatr Nephrol, 2004. **19**(3): p. 345-8.
33. Birk, P.E., et al., *Cardiac tamponade as a terminal event in the hemolytic uremic syndrome in childhood.* Pediatr Nephrol, 1994. **8**(6): p. 754-5.
34. Palanca Arias, D., M. Lopez Ramon, and L. Jimenez Montanes, *Biomarkers detect involvement of acute myocardial injury in a paediatric haemolytic-uraemic syndrome patient.* Cardiol Young, 2016. **26**(5): p. 983-6.
35. Khalid, M. and S. Andreoli, *Extrarenal manifestations of the hemolytic uremic syndrome associated with Shiga toxin-producing Escherichia coli (STEC HUS).* Pediatr Nephrol, 2019. **34**(12): p. 2495-2507.
36. Gallo, E.G. and C.A. Gianantonio, *Extrarenal involvement in diarrhoea-associated haemolytic-uraemic syndrome.* Pediatr Nephrol, 1995. **9**(1): p. 117-9.
37. Karmali, M.A., et al., *The association between idiopathic hemolytic uremic syndrome and infection by verotoxin-producing Escherichia coli.* J Infect Dis, 1985. **151**(5): p. 775-82.
38. Boyd, B. and C. Lingwood, *Verotoxin receptor glycolipid in human renal tissue.* Nephron, 1989. **51**(2): p. 207-10.
39. Zoja, C., S. Buelli, and M. Morigi, *Shiga toxin-associated hemolytic uremic syndrome: pathophysiology of endothelial dysfunction.* Pediatr Nephrol, 2010. **25**(11): p. 2231-40.
40. Endo, Y., et al., *The mechanism of action of ricin and related toxic lectins on eukaryotic ribosomes. The site and the characteristics of the modification in 28 S ribosomal RNA caused by the toxins.* J Biol Chem, 1987. **262**(12): p. 5908-12.
41. Thoms, M., et al., *Structural basis for translational shutdown and immune evasion by the Nsp1 protein of SARS-CoV-2.* Science, 2020.
42. Iba, T., et al., *Sepsis-associated disseminated intravascular coagulation and its differential diagnoses.* J Intensive Care, 2019. **7**: p. 32.
43. Gavriatopoulou, M., et al., *Organ-specific manifestations of COVID-19 infection.* Clin Exp Med, 2020.
44. Gupta, A., et al., *Extrapulmonary manifestations of COVID-19.* Nat Med, 2020. **26**(7): p. 1017-1032.
45. Coldewey, S.M., et al., *Identification of cardiovascular and molecular prognostic factors for the medium-term and long-term outcomes of sepsis (ICROS): protocol for a prospective monocentric cohort study.* BMJ Open, 2020. **10**(6): p. e036527.
46. La Rosee, F., et al., *The Janus kinase 1/2 inhibitor ruxolitinib in COVID-19 with severe systemic hyperinflammation.* Leukemia, 2020. **34**(7): p. 1805-1815.
47. Berrios, R.A.S., et al., *Correlation of left ventricular systolic dysfunction determined by low ejection fraction and 30-day mortality in patients with severe sepsis and septic shock: A systematic review and meta-analysis.* Journal of Critical Care, 2014. **29**(4): p. 495-499.
48. Landesberg, G., et al., *Diastolic dysfunction and mortality in severe sepsis and septic shock.* European Heart Journal, 2012. **33**(7): p. 895-903.
49. Vieillard-Baron, A., *Septic cardiomyopathy.* Ann Intensive Care, 2011. **1**(1): p. 6.
50. Müller-Werdan, U., et al., *Mikrozirkulationsstörung, zytopathische Hypoxie und septische Kardiomyopathie*, in *Sepsis und MODS*, K. Werdan, et al., Editors. 2016, Springer Science and Business Media. p. 137-139.
51. Lang, R.M., et al., *Recommendations for cardiac chamber quantification by echocardiography in adults: an update from the American Society of Echocardiography and the European Association of Cardiovascular Imaging.* J Am Soc Echocardiogr, 2015. **28**(1): p. 1-39 e14.
52. Pendlebury, S.T., et al., *Telephone assessment of cognition after transient ischemic attack and stroke: modified telephone interview of cognitive status and telephone Montreal Cognitive Assessment versus face-to-face Montreal Cognitive Assessment and neuropsychological battery.* Stroke, 2013. **44**(1): p. 227-

9.
53. Heuschmann, P.U., et al., *[The reliability of the german version of the barthel-index and the development of a postal and telephone version for the application on stroke patients].* Fortschr Neurol Psychiatr, 2005. **73**(2): p. 74-82.
54. Spitzer, C., et al., *[The short version of the Brief Symptom Inventory (BSI -18): preliminary psychometric properties of the German translation].* Fortschr Neurol Psychiatr, 2011. **79**(9): p. 517-23.
55. Martin, A., et al., *Messung chronischer Erschöpfung - Teststatistische Prüfung der Fatigue Skala (FS).* Zeitschrift für Klinische Psychologie und Psychotherapie, 2010. **39**(1): p. 33-44.
56. Lawton, M.P. and E.M. Brody, *Assessment of older people: self-maintaining and instrumental activities of daily living.* Gerontologist, 1969. **9**(3): p. 179-86.
57. Rabin, R., et al., *From translation to version management: a history and review of methods for the cultural adaptation of the EuroQol five-dimensional questionnaire.* Value Health, 2014. **17**(1): p. 70-6.
58. Radtke, F.M., et al., *[The Post-Traumatic Stress Syndrome 14-Questions Inventory (PTSS-14) - Translation of the UK-PTSS-14 and validation of the German version].* Anasthesiol Intensivmed Notfallmed Schmerzther, 2010. **45**(11-12): p. 688-95.
59. Klasen, B.W., et al., *Validation and reliability of the German version of the Chronic Pain Grade questionnaire in primary care back pain patients.* Psychosoc Med, 2004. **1**: p. Doc07.
60. von Elm, E., et al., *The Strengthening the Reporting of Observational Studies in Epidemiology (STROBE) Statement Guidelines for Reporting Observational Studies.* Epidemiology, 2007. **18**(6): p. 800-804.
61. Collins, G.S., et al., *Transparent reporting of a multivariable prediction model for individual prognosis or diagnosis (TRIPOD): the TRIPOD Statement.* Bmc Medicine, 2015. **13**.

14 Anhang

14.1 SOFA-Score

14.2 APACHE-Score

14.3 SAPS II Score

14.4 Charlson Comorbidity Index

14.5 CAM-ICU

14.6 COVID Hyperinflammations-Score
