# Supplementary material for: Identification of cardiovascular and molecular prognostic factors for the morbidity and mortality in COVID-19-sepsis (ICROVID): Protocol for a prospective multi-centre cohort study
Source: PLoS One. 2022 Jun 3;17(6):e0269247. doi: 10.1371/journal.pone.0269247 (PMC9165863; doi:10.1371/journal.pone.0269247)
Supplement: S2 File — (PDF) [file pone.0269247.s003.pdf]

Study protocol

Identifikation kardiovaskulärer und molekularer Prognosefaktoren für die Morbidität und Mortalität bei COVID-19-Sepsis

Identification of cardiovascular and molecular prognostic factors for the morbidity and mortality in COVID-19-sepsis

Acronym: ICROVID

Protocol version: 1.2 dated 03.06.2021

Principal Investigator:

Univ.-Prof. Dr. Dr. med. Sina M. Coldewey

Translational Septomics, Center for Innovation Competence Septomics

Department of Anaesthesiology and Intensive Care Medicine, Jena University Hospital

E-mail: sina.coldewey@med.uni-jena.de, Phone: 03641 9323190

The following persons agree to the contents of the study protocol and indicate this with their signature.

**Univ-Prof. Dr. Dr. med. Sina M. Coldewey**<br>**Principal Investigator(PI)**	**Date**	**Signature**
**Charles Neu, MD**<br>**Deputy PI**	**Date**	**Signature**
**Philipp Baumbauch**<br>**Study Coordinator**	**Date**	**Signature**
**Prof. Dr. med. Michael Bauer**<br>**Head of the department**	**Date**	**Signature**

Table of Contents

1 General information 7

1.1 Participating persons, institutions, committees 7

1.2 Synopsis 11

1.3 Procedure and time plan 14

2 List of abbreviations 16

3 Background 18

3.1 Sepsis 18

3.2 COVID-19 sepsis 18

3.3 Cardiovascular complications with COVID-19 19

3.4 Microangiopathic complications of COVID-19 19

3.5 Aims and objectives of the study 21

4 Study endpoints 21

5 Study population 25

5.1 Inclusion criteria 25

5.2 Exclusion criteria 25

5.3 Definition of sepsis/septic shock criteria (Sepsis 3 criteria) 25

5.4 Definition of the criteria septic cardiomyopathy 26

6 Study Outline 27

6.1 Sepsis patients (COVID-19 and influenza) 27

6.1.1 Screening and patient identification list 27

6.1.2 Informed consent 27

6.1.2.1 Consenting patients 27

6.1.2.2 Non-consenting patients 27

6.1.2.3 No or withdrawal of consent 28

6.1.3 Documentation in the course of the study 29

6.1.4 Study-related measures 29

6.1.5 End of study / end of follow-up 29

6.2 Evaluation phase 29

7 Description of the methods 29

7.1 Clinical examinations 29

7.1.1 TEE/TTE 29

7.1.2 Transient elastography 29

7.2 Laboratory tests 30

7.2.1 survey of routine parameters 30

7.2.2 Study-related examinations 30

7.3 Health-related quality of life and long-term outcomes 31

8 Adverse events 31

9 Data management and quality assurance 31
9.1 Patient Identification List 31
9.2 List of responsibilities 31
9.3 Data collection/documentation forms 32
9.4 Data processing 32
9.5 Retention of study documents 32
9.6 Data protection 32
10 Biometrics 33
10.1 Endpoints 33
10.2 Definition of evaluation cohorts 33
10.3 Case number planning 34
10.4 Interim/evaluation 34
10.5 Further statistical analysis 34
10.6 Presentation of the results 34
11 Publication / Use of results / Registration of data collection 35
11.1 Final report and publications 35
11.1.1 Publication of the study protocol 35
11.1.2 Final report 35
11.1.3 Analyses and publications 35
11.2 Citation 35
11.3 Authors 35
11.4 Registration 35
12 Ethical concerns and administrative arrangements 36
12.1 Declaration of Helsinki and Good Clinical Practice 36
12.2 Ethics Committees 36
12.3 Subsequent changes 36
12.4 Funding 36
13 Literature 37
14 Appendix 40
14.1 SOFA score 40
14.2 APACHE score 41
14.3 SAPS II Score 42
14.4 Charlson Comorbidity Index 43
14.5 CAM-ICU 44
14.6 COVID Hyperinflammation Score 45

1 General information

1.1 Participating persons, institutions, committees

**Principal Investigator (PI)**	**Univ.-Prof. Dr. Dr. med. Sina Coldewey**<br>Department of Anesthesiology and Intensive Care Medicine<br>Centre for Innovation Competence (ZIK) Septomics<br>Jena University Hospital<br>Am Klinikum 1, 07747 Jena<br>Tel: 03641-9323190<br>Email: sina.coldewey@med.uni-jena.de
**Deputy PI**	**Charles Neu, MD**<br>Department of Anesthesiology and Intensive Care Medicine<br>ZIK Septomics<br>Jena University Hospital<br>Am Klinikum 1, 07747 Jena<br>Tel: 03641-9323166<br>Email: charles.neu@med.uni-jena
**Study Coordinator**	**Dipl.-Psych. Philipp Baumbach**<br>Department of Anesthesiology and Intensive Care Medicine<br>ZIK Septomics<br>Jena University Hospital<br>Am Klinikum 1, 07747 Jena<br>Tel: 03641-9325798<br>Email: philipp.baumbach@med.uni-jena.de
**Biometrician (consulting)**	**Prof. Dr. André Scherag**<br>Center for Sepsis Control and Care (CSCC)<br>Clinical epidemiology<br>Jena University Hospital<br>Salvador-Allende-Platz 27, 07747 Jena<br>Tel: 03641-______<br>Email: andre.scherag@med.uni-jena

Study physicians

**Univ.-Prof. Dr. Dr. med. Sina Coldewey**<br>Department of Anesthesiology and Intensive Care Medicine (DAI)<br>ZIK Septomics<br>Jena University Hospital<br>Am Klinikum 1, 07747 Jena<br>Tel: 03641-9323190<br>Email: sina.coldewey@med.uni-jena.de	**Dr. med. Heike Dorow**<br>DAI<br>ZIK Septomics<br>Jena University Hospital<br>Am Klinikum 1, 07747 Jena<br>Tel: 03641-9323168<br>Email: heike.dorow@med.uni-jena
**Ricardo Esper Treml**<br>DAI<br>ZIK Septomics<br>Jena University Hospital<br>Am Klinikum 1, 07747 Jena<br>Tel: 03641-9323261<br>Email: ricardo.espertreml@med.uni-jena.de	**Juliane Goetze**<br>DAI<br>ZIK Septomics<br>Jena University Hospital<br>Am Klinikum 1, 07747 Jena<br>Tel: 03641-9323250<br>Email: juliane.goetze@med.uni-jena

Study physicians (continued)

Jan Höfer
DAI
Jena University Hospital
Am Klinikum 1, 07747 Jena
Tel: 03641-9______
Email: jan.hoefer@med.uni-jena.de

Prof. Dr. med. Andreas Kortgen
DAI
Jena University Hospital
Am Klinikum 1, 07747 Jena
Tel: 03641-9 [redacted]
Email: andreas.kortgen@med.uni-jena.de

Charles Neu, MD
ZIK Septomics
DAI
Jena University Hospital
Am Klinikum 1, 07747 Jena
Tel: 03641-9323166
Email: charles.neu@med.uni-jena

PD Dr. med. Philipp Reuken
Clinic for Internal Medicine IV
Jena University Hospital
At the clinic 1
07747 Jena
Tel: 03641 9 [redacted]
Email: philipp.reuken@med.uni-jena.de

Dr. med. Christiane Schmidt-Winter
DAI
ZIK Septomics
Jena University Hospital
Am Klinikum 1, 07747 Jena
Tel: 03641-______
Email: christiane.schmidt-winter@med.uni-jena.de

Kornel Skitek
DAI
ZIK Septomics
Jena University Hospital
Am Klinikum 1, 07747 Jena
Tel: 03641-______
Email: kornel.skitek@med.uni-jena.de

other study physicians (senior physicians of the intensive care unit)

PD Dr. Dr. med. Frank Bloos
DAI
Jena University Hospital
Am Klinikum 1, 07747 Jena
Tel: [redacted]
Email: [redacted]

Martin Brauer, MD
DAI
Jena University Hospital
Am Klinikum 1, 07747 Jena
Tel: [redacted]
Email: [redacted]

Carsten Herzog, MD
DAI
Jena University Hospital
Am Klinikum 1, 07747 Jena
Tel: [redacted]
Email: [redacted]

Michael Hofmann, MD
DAI
Jena University Hospital
Am Klinikum 1, 07747 Jena
Tel: [redacted]
Email: [redacted]

Dr. med. Hendrik Rüddel
DAI
Jena University Hospital
Am Klinikum 1, 07747 Jena
Tel: [redacted]
Email: [redacted]

Dr. med. Ingo Salzmann
DAI
Jena University Hospital
Am Klinikum 1, 07747 Jena
Tel: [redacted]
Email: [redacted]

Mark Simon, MD
DAI
Jena University Hospital
Am Klinikum 1, 07747 Jena
Tel: [redacted]
Email: [redacted]

Helga Skupin, MD
DAI
Jena University Hospital
Am Klinikum 1, 07747 Jena
Tel: [redacted]
Email: [redacted]

other study physicians (senior physicians of the intensive care unit, continued)

Daniel Thomas-Rüddel, MD
DAI
Jena University Hospital
Am Klinikum 1, 07747 Jena
Tel: [redacted]
Email: [redacted]

PD Dr. med. Christian von Löffelholz
DAI
Jena University Hospital
Am Klinikum 1, 07747 Jena
Tel: 03641-9______
Email: [redacted]

Dr. med. Isabella Westermann
DAI
Jena University Hospital
Am Klinikum 1, 07747 Jena
Tel: [redacted]
Email: [redacted]

Prof. Dr. med. Johannes Winning
DAI
Jena University Hospital
Am Klinikum 1, 07747 Jena
Tel: [redacted]
Email: [redacted]

Data Manager

Cornelia Eichhorn
Center for Clinical Studies
Jena University Hospital
Salvador-Allende-Platz 27, 07747 Jena
Tel: 03641-9______
E-mail: [redacted]. uni-jena.de

First-voting Ethics Committee

Ethics Committee of the Friedrich Schiller University Jena
P.O. Box
Bachstraße
18, 07740 Jena
Tel: 03641-933______
Email: ethikkommission@med.uni-jena.de

Cooperation partner

Prof. Dr. Michael Bauer
Center for Sepsis Control and Care (CSCC)
DAI
Jena University Hospital
Am Klinikum 1, 07747 Jena
Tel: 03641-9323100
Email: michael.bauer@med.uni-jena.de

PD Dr. Christian Kosan
Center for Molecular Biomedicine
Friedrich Schiller University Jena
Hans-Knöll-Str.
207745 Jena
Tel: 03641-9_____
Email: christian.kosan@uni-jena.de

Prof. Dr. Andreas Stallmach
Clinic for Internal Medicine IV
- Gastroenterology, hepatology,
Infectious diseases, interdisciplinary endoscopy -
Jena University Hospital
At the clinic 1
07747 Jena
Tel: 03641-9324400
Email: Andreas.Stallmach@med.uni-jena.de

PD Dr. Dr. Michael Kiehntopf
Institute for Clinical Chemistry and Laboratory Diagnostics
Jena University Hospital
Am Klinikum 1, 07747 Jena
Tel: 03641-9325 000
Email: michael.kiehntopf@med.uni-jena

Prof. Dr. André Scherag
Center for Sepsis Control and Care (CSCC)
Clinical epidemiology
Jena University Hospital
Salvador-Allende-Platz 27, 07747 Jena
Tel: 03641-9______
Email: andre.scherag@med.uni-jena

1.2 Synopsis

**Title (German)**	Identifikation kardiovaskulärer und molekularer Prognosefaktoren für die Morbidität und Mortalität bei COVID-19-Sepsis
**Title (Engl.)**	Identification of cardiovascular and molecular prognostic factors for the morbidity and mortality in COVID-19-sepsis
**Short name (acronym)**	ICROVID
**Population / Indication**	▪ adult patients with COVID-19-associated sepsis and indication for intensive care therapy<br>o *with septic cardiomyopathy*<br>o *without septic cardiomyopathy*<br><br>▪ adult patients with influenza-associated sepsis and indication for intensive therapy<br><br>Patients from the ICROS study will serve as control collectives:<br>▪ adult patients with sepsis treated in an intensive care unit<br>▪ adult, healthy subjects (G)
**Design**	Prospective, multicenter cohort study
**Targets**	▪ Comprehensive characterization of acute, post-acute, mid- and long-term patient status with focus on cardiovascular and metabolic changes in COVID-19 sepsis.<br>▪ Identification of potential biomarkers and theragnostic targets/molecules as well as cardiovascular, clinical-epidemiological and laboratory prognostic factors for short/medium/long-term morbidity and mortality in COVID-19 sepsis.
**Visits**	T_0 : Screening/Inclusion<br>T_1 : 3 ± 1 d<br>T_2 : 7 ± 1 d<br>T_3 : 14 ± 1 d or (up to 3 d before) hospital discharge.<br><br>Follow-up phase:<br>(telephone interview and questionnaire)<br>T_4 : after 28 d<br>T_5 : after 90 d<br>T_6 : after 180 d<br>after initial diagnosis of sepsis
**Target figures**	**Primary endpoints**<br>▪ Mortality differences between COVID-19 sepsis patients with vs. without the presence of septic cardiomyopathy at the time point 3 months after initial diagnosis of COVID-19 sepsis (T_5)<br><br>**Secondary endpoints**<br>▪ Mortality differences between COVID-19 sepsis patients with vs. without the presence of septic cardiomyopathy at the 6-month time point after initial diagnosis of COVID-19 sepsis (T_5)<br>▪ Incidence of cardiovascular events in patients with COVID-19 sepsis in the acute (T_1 , T_2), post-acute (T_3 -T_4) and long-term (T_5 -T_6) course of disease.<br>▪ Differences in the incidence of septic cardiomyopathy during the acute course of disease (T_1 , T_2) in patients with COVID-19-associated sepsis and patients with

<table>
<tr><td></td><td>influenza-associated sepsis
▪ Differences in the incidence of cardiovascular events in patients with COVID19-associated sepsis and patients with influenza-associated sepsis in the acute (T_1 , T_2), post-acute (T_3 -T_4) and long-term (T_5 -T_6) course.
For further clinical questions see section 4</td></tr>
<tr><td>Number of study participants</td><td>Patients with COVID-19-associated sepsis: up to 160
Patients with influenza-associated sepsis: up to 160</td></tr>
<tr><td>Inclusion criteria</td><td>Patients with COVID-19-associated and influenza-associated sepsis
▪ Age ≥ 18 years
▪ written declaration of consent of the patient or his legal representative
▪ confirmed SARS-CoV-2 <u>or</u> confirmed influenza virus infection
▪ respiratory signs
▪ Indication for intensive therapy
▪ Sepsis or septic shock according to sepsis-3 criteria
▪ Sepsis onset (infection-related SOFA score change ≥ 2) not older than 4 d (first blood draw within 4 d of sepsis onset).</td></tr>
<tr><td>Exclusion criteria</td><td>Patients with COVID-19-associated and influenza-associated sepsis
▪ cardiac surgery ≤ 12 months
▪ significant cardiac disease
o Endocarditis
o higher-grade valvular disease (severe/grade 3 valvular disease, symptomatic aortic stenosis, moderate mitral regurgitation with impaired ejection fraction or clinical symptoms)
o complex structural congenital heart disease (e.g. TGA, tetralogy of Fallot, endocardial cushion defects, etc.)
o hemodynamically relevant shunt vitium
o pre-existing, significant limitations of cardiac output (ejection fraction < 45 % or 10 % below normal)
o pre-existing pulmonary hypertension
o Z. n. myocardial infarction (≤ 1 year)
o Post-heart transplantation
▪ cardiopulmonary resuscitation within the last 4 weeks before sepsis onset
▪ Z. n. pneumectomy
▪ Liver cirrhosis Child C
▪ Contraindication for TEE (e.g. esophageal resection, higher grade esophageal varices) and insufficient sound conditions for TTE
▪ pre-existing chronic terminal renal failure with dialysis
▪ Sepsis within the last 8 months
▪ Pregnancy/breastfeeding
▪ Therapy restriction or cessation
▪ Life expectancy ≤ 6 months due to secondary diseases
▪ previous participation in this study</td></tr>
<tr><td>Schedule</td><td>Exam-related study duration
<table>
<tr><td>Duration Recruitment</td><td>18 months</td></tr>
<tr><td>Inclusion of first patient</td><td>first quarter 2021</td></tr>
<tr><td>Inclusion of last patient</td><td>second quarter 2022</td></tr>
<tr><td>Last Follow-Up</td><td>fourth quarter 2022</td></tr>
<tr><td>Interim evaluation</td><td>none planned</td></tr>
<tr><td>Evaluation</td><td>after complete data sets are available 6 months after initial diagnosis of COVID-19 sepsis or influenza sepsis,</td></tr>
</table></td></tr>
</table>

		respectively.
	**Total duration of study**	36 months
**Statistical methods**	The primary endpoint will be analyzed using Cox regression, with primary interest in the group comparison of the presence of septic cardiomyopathy on survival time. Adequate standard statistical procedures will be used to answer the secondary endpoints and other questions. In the descriptive analyses, all parameters will be reported according to their scale level (relative and absolute frequencies, location and dispersion measures). Group comparisons are analyzed using adequate procedures depending on the distributional properties of the target parameters. The identification of prognostic factors or predictors is primarily of an exploratory nature. Appropriate methods of multivariate statistics, in particular correlation and regression analyses, are used.	
**Funding**	Federal Ministry of Education and Research, project funding: Coldewey - ICROVID: Identification of cardiovascular and molecular prognostic factors for morbidity and mortality in COVID-19 sepsis (FKZ 03COV07) and Clinic for Anaesthesiology and Intensive Care Medicine of UKJ	

1.3 Procedure and time plan

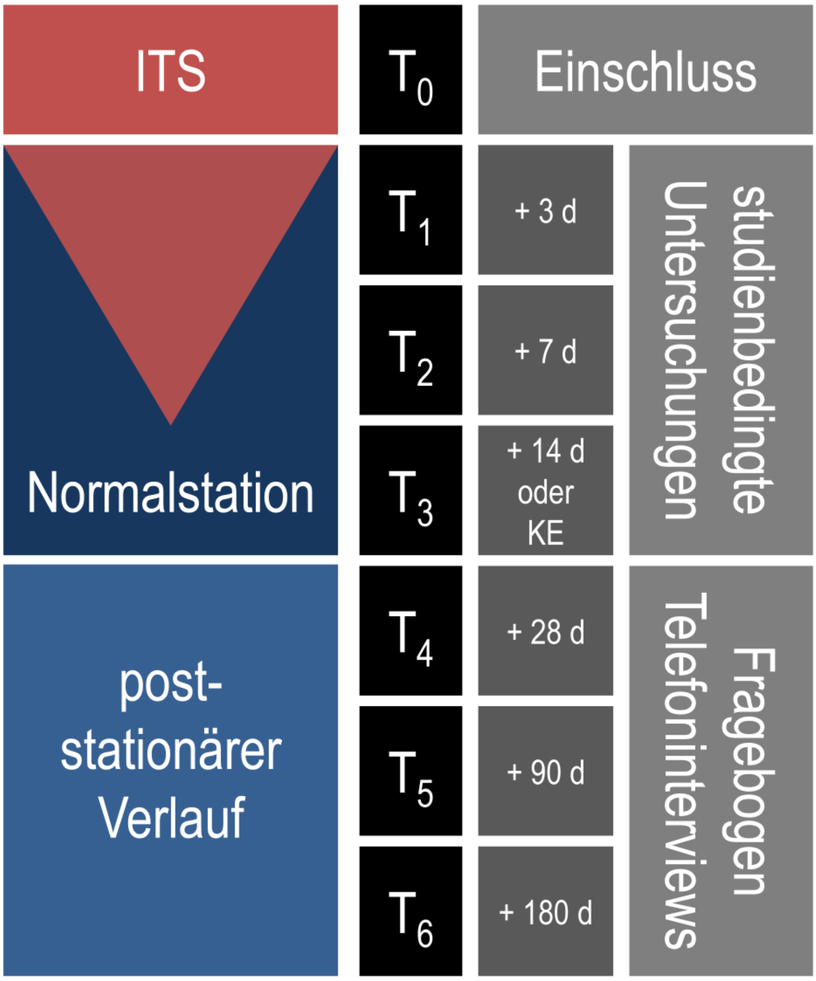

Figure 1. Summary of rounds over the course of treatment. Study-related examinations (e.g., echocardiography and blood sampling) take place during the acute or post-acute illness phase and end with T_3 (14 days after initial diagnosis of COVID-19/influenza-associated sepsis or hospital discharge, KE). Further surveys will be conducted by telephone interviews or questionnaires.

Table 1. Rounds schedule.

Date of survey	Screening	T_0	T_1	T_2	T_3	T_4	T_5	T_6
**Inclusion**								
Declaration of consent	X							
Inclusion/exclusion criteria	X							
**COVID-19 sepsis associated variables**								
SIRS criteria		X	X	X	X			
SOFA score		X	X	X	X			
APACHE II and SAPS II		X						
COVID-19 specific variables		X	X	X	X			
CAM-ICU		X	X	X	X			
**Previous story**								
Demographics/ Additional demographic information		X						
Comorbidities: Charlson Comorbidity Index		X						
Comorbidities: Cardiovascular risk factors		X						
Comorbidities: previous cardiovascular diseases		X						
previous cardiological findings and home medication		X						
Medical history and patient history		X				X		
**instrument-based examinations**								
Echocardiography (*TEE/TTE*)			X	X				
transient elastography[1]			X	X				
advanced hemodynamic monitoring[2]			X	X	X			
Microbiome[1]				X				
**Blood tests**								
Routine lab		X	X	X	X			
academic laboratory[3]			X	X	X			
**Course of treatment/data**								
Infection		X	X	X	X		X	X
Microbiology		X	X	X	X			
cardiovascular events during hospital treatment		X	X	X	X	X		
Hospital treatment/discharge data						X		
physiological parameters		X	X	X	X			
Concomitant medication		X	X	X	X		X	X
**Course of the disease**								
Survival status			X	X	X	X	X	X
Progressive medical history							X	X
cardiovascular events after hospital discharge							X	X
Quality of life (EQ-5D-3L)							X	X
Assessment of long-term effects (questionnaire/telephone interview)							X	X

T_0 : Screening/Inclusion
T_1 : 3 ± 1 d after sepsis onset
T_2 : 7 ± 1 d after sepsis onset
T_3 : 14 ± 1 d after sepsis onset or (up to 3 d before) hospital discharge.
T_4 : after 28 d after sepsis onset
T_5 : after 90 d after sepsis onset
T_6 : after 180 d after sepsis onset

[1] optional implementation in individual study centers (e.g. Jena University Hospital)
[2] extended haemodynamic monitoring (e.g. PiCCO/PAK) is only carried out in a proportion of patients as Routine diagnostics and not as a study-related measure.
[3] in blood and urine for the analysis of surrogate parameters for cardiac dysfunction and other organ dysfunctions, Surrogate parameters for endothelial barrier/glycocalyx disruption and analysis of immune status, metabolome, lipidome

2 List of abbreviations

**28S rRNA**	ribosomal ribonucleic acid
**Fig.**	figure
**ADL**	acitivities of daily living
**ANV**	acute renal failure
**APACHE II**	Acute Physiology and Chronic Health Evaluation II
**ARDS**	acute respiratory distress syndrome
**BB**	blood count
**BGA**	blood gas analysis
**BMBF**	Federal Ministry of Education and Research
**BNP**	B-type natriuretic peptide
**COVID-19**	coronavirus disease 2019
**CRF**	case report form
**CSCC**	Center for Sepsis Control and Care
**d**	day/s
**DIC**	disseminated intravascular coagulation
**dL**	decilitre
**e-CRF**	electronic case report form
**EHEC**	enterohaemorrhagic Escherichia coli
**ECG**	electrocardiography
**et al.**	et alia
**GCP**	Good Clinical Practice
**GCS**	Glasgow Coma Scale
**h**	hour/s
**https**	HyperText Transfer Protocol Secure
**IADL**	instrumental acitivities of daily living
**ICMJE**	International Committee of Medical Journal Editors
**ICROS**	Acronym of the study "Identification of cardiovascular and molecular prognostic factors for mid- and long-term morbidity and mortality in sepsis".
**IL**	Interleukin
**INTERMACS**	Interagency Registry for Mechanically Assisted Circulatory Support
**ISF**	investigator site file (Study Center Folder)
**ITS**	ICU
**M**	month/s
**max.**	maximum
**mg**	milligram
**min**	minute
**mmHg**	millimeter mercury column
**NSP1**	non-structural protein 1
**NWG**	Junior Research Group
**PAH**	pulmonary artery catheter
**PI**	principal investigator
**PiCCO**	Pulse Contour Cardiac Output
**PTSD**	post-traumatic stress disorder
**s**	seconds

**SAPS II**	Simplified Acute Physiology Score
**SARS-CoV-2**	severe acute respiratory syndrome coronavirus 2
**SIRS**	systemic inflammatory response syndrome
**SOFA**	sequential organ failure assessment
**STROBE**	Strengthening the reporting of observational studies in epidemiology
**Stx**	shiga toxin
**T**	time - point in time
**TEE**	transesophageal echocardiography
**Tel.**	phone
**t-MoCa**	Montreal Cognitive Assessment (telephone)
**TRIPOD**	Transparent reporting of a multivariable prediction model for individual prognosis or diagnosis
**TTE**	transthoracic echocardiography
**UKJ**	Jena University Hospital
**W**	week/s
**WHO**	World Health Organization
**ZIK**	Center for Innovation Competence
**ZKS**	Center for clinical studies

3 Background

3.1 Sepsis

In sepsis, a dysregulated host response resulting from infection leads to functional impairment or failure of one or more organ systems [1]]. The underlying infection can be caused by a variety of pathogens: mainly bacteria and to a lesser extent viruses, fungi and parasites. [2]]. The highest incidence and mortality of sepsis is recorded in the areas with the lowest socio-demographic index [3]. But also in countries with a high standard of living sepsis is a frequently underestimated disease. Of causal importance here is also the increasing possibility of using invasive procedures, such as major surgery or immunomodulatory chemotherapy, which often lead to impaired immune function in patients. [4]]. A very recent example of the great social, economic and political importance of sepsis is the current pandemic. It was triggered by an outbreak of infections with the new beta-coronavirus SARS-CoV2 -(*severe acute respiratory syndrome coronavirus* 2) in the Chinese city of Wuhan in December 2019 [5]]. The disease caused by this virus has been named COVID-19 (*coronavirus disease 2019*) by the World Health Organization (WHO). Within a very short time, the COVID-19 pandemic has pushed healthcare systems worldwide to their limits.

Despite extensive research over the past decades, no clinical trial has yet led to the implementation of causal therapy for sepsis in clinical practice. Scientific findings suggest that patients with sepsis should be stratified according to their clinical and molecular phenotype and risk profile, and should receive personalized therapy [6]]. Such a personalized approach has been used in the field of cancer therapy for a long time. [7]]. Patients with sepsis form a very heterogeneous patient population. [6]]. Therefore, it seems particularly important that clinical trials take into account the diversity of patients in terms of individual medical history, clinical presentation, individual molecular and immunological host response and disease-causing pathogen. Innovative translational research approaches are needed to identify new targets that can be used to develop diagnostic and therapeutic strategies in this severe systemic disease. [8]]. Good initial approaches are provided by a study by Seymour *et al. in* which sepsis patients could be classified into 4 clinical phenotypes with different risk profiles on the basis of clinical criteria. [9]]. Patients with COVID19 sepsis also -seem to develop a specific phenotype, which needs to be further characterized by clinical and molecular investigations.

3.2 COVID-19 sepsis

It is now known that the severity of disease progression after SARS-CoV2 infection -varies widely between individuals, without knowing the -causative molecular mechanisms. The typical COVID-19 symptoms include fever and dry cough, loss of taste and smell [10]] a loss of taste and smell [11]]. In addition to asymptomatic infections and mild courses of COVID-19, severe courses with the development of pneumonia with pronounced hypoxia, acute respiratory distress syndrome (ARDS) or multiorgan failure with or without fatality have been observed worldwide. In conclusion, WHO reported that during the initial outbreak in China, about 80% of laboratory-confirmed SARS-CoV-2-positive patients had mild to moderate disease, while 13.8% had severe disease and 6.1% had critical disease. [5]]. In Germany, too, about 5% of patients develop life-threatening organ dysfunctions as a result of SARS-CoV-2 infection, requiring intensive care (www.divi.de/register/tagesreport). By definition, these very severe courses of COVID-19 disease can be subsumed under the generic term sepsis. [1]]. Together with a pronounced reaction of the immune system (so-called cytokine storm), these patients suffer from the above-mentioned pronounced lung damage with severe oxygenation disturbance. They often require invasive ventilation, but other organ dysfunctions, such as renal and liver failure, must also be treated. Thus, the group of sepsis patients with COVID-19 can be considered a special phenotype of sepsis. Several authors also report a high incidence of cardiac complications [12]]the occurrence of a pronounced endothelial damage [13]] and coagulation disorders with an accumulation of life-threatening thromboembolic events. [12, 14, 15]. According to current figures from the DIVI Intensive Care Registry and the Robert Koch Institute, approximately 25% of COVID-19 patients receiving intensive care die (www.divi.de/register/tagesreport). The extent to which mortality and long-term morbidity are influenced

by the occurrence of a so-called septic cardiomyopathy is unknown. Due to the novelty of the disease, a conclusive description of all symptoms and their frequencies is not yet available. The state of knowledge regarding this clinical picture is currently changing daily.

3.3 Cardiovascular complications with COVID-19

Many authors describe that patients with viral influenza have a higher risk of suffering cardiovascular complications [16, 17]]. Influenza with primary pulmonary organ involvement is often associated with bacterial superinfection and not infrequently results in sepsis [18]]. Extrapulmonary organ dysfunction, such as cardiac involvement due to the development of myocarditis or cardiomyopathy, has been described. [19]]. Cardiovascular complications are also common in COVID-19 sepsis. However, unlike influenza viruses, at-risk groups cannot protect themselves against SARS-CoV-2 in the absence of a suitable and available vaccine. The molecular mechanisms and the significance of cardiovascular events for the medium- and long-term course of disease in patients with COVID-19 sepsis have not yet been elucidated.

Cardiovascular complications described in patients with COVID-19 include myocardial infarction, cardiac arrhythmias, arrhythmias, pericardial tamponade, and thromboembolic events such as pulmonary embolism (reviewed in [20]]). Myocardial injury, as evidenced by an increase in the cardiac enzyme troponin, occurs in approximately 20% of critically ill COVID-19 patients [21-23]]. In a monocentric study, laboratory-detected cardiac injury was associated with significantly increased mortality [22]]. However, in this prospective study, the end point was variable (follow-up day 1 to 37), making it difficult to accurately estimate mortality. In addition, cardiac imaging was not performed, so any association between laboratory changes and cardiac function was not investigated. Thromboembolic events in COVID-19 patients requiring intensive care have been observed in studies to date with a frequency of approximately 17-30% [24-26] and have highlighted the need for adequate inhibition of blood clotting [27]]. Functional limitations of cardiac pumping function have also been described at 12% . [10]]. The causes of this are not clear. A cardiac decompensation of a pre-existing heart disease or a myocarditis caused by the virus is conceivable. In autopsies of 39 deceased patients with COVID-19, significant amounts of SARS-CoV-2 virus were detected in 41 % of the heart tissue. [28]]. Because no cellular inflammatory response was detected, the presence of myocarditis in these patients remains unclear. Finally, the sepsis that patients develop may also cause septic cardiomyopathy, which is also associated with reduced pump function. [29]].

3.4 Microangiopathic complications of COVID-19

Thrombotic microangiopathies may require intensive medical therapy. Pulmonary autopsies from 7 COVID-19 patients revealed thrombotic microangiopathy of the pulmonary vessels [13]]. Furthermore, these lungs showed specific features compared to lungs of patients with influenza, such as a 9 times more frequent thrombosis of the alveolar capillaries. Another disease, usually requiring intensive care, which interestingly shows both clinical and pathophysiological similarities to COVID-19, is the hemolytic uremic syndrome. This syndrome develops in 5-10% of cases as a result of infection with enterohaemorrhagic *Escherichia coli* (EHEC). [30]]. Pathognomonic is the clinical triad of microangiopathic hemolytic anemia, thrombocytopenia and acute renal failure. As with SARS-CoV-2 infection, it is not yet known why, after infection with EHEC, some patients develop only mild symptoms but others become life-threateningly ill. In both COVID-19 and hemolytic uremic syndrome, severe courses can be considered a defined sepsis entity. As another common feature, thromboembolic complications and, in case reports, cardiac complications also occur in the hemolytic uremic syndrome [31-34]. These complications range from left ventricular hypertrophy and impaired cardiac function to pericardial effusions and chronic heart failure. [35]]. Autopsies of 64 patients with hemolytic uremic syndrome found cardiac complications in 19 cases, the majority of which were caused by thrombotic microangiopathy in the myocardium [36]]. The exact extent of cardiovascular complications in the hemolytic uremic syndrome and their influence on the long-term course has not yet been prospectively studied, which is also due to the sporadic occurrence of the disease.

Interestingly, the two diseases - COVID-19 sepsis and hemolytic uremic syndrome - have molecularly similar pathomechanisms. The disease-causing pathogens - SARS-CoV-2 and EHEC - possess pathogenicity factors that inhibit protein biosynthesis at the translational level. A major virulence factor of EHEC, Shiga toxin (Stx) [37]]binds the globotriaosylceramide-3 receptor, which is particularly strongly expressed by renal microvascular endothelial

cells, thus mainly affecting the kidney. [38]]. Stx is taken up into the cell via endocytosis and leads to an inhibition of protein biosynthesis by deactivating the catalytic [39]] by deactivation of the catalytically active 28S rRNA. [40]]. In contrast, the protein Nsp1 expressed by SARS-CoV-2 binds the ribosomal 40S subunit and thus blocks protein biosynthesis. [41]] The endothelial cell damage that occurs leads to a prothrombotic milieu, microangiopathic thrombosis, and activation of the immune system [39]]. Thereby, pathophysiological parallels to disseminated intravascular coagulopathy (DIC) during sepsis occur [42].

A major difference between the two diseases is in terms of their organotropism: whereas COVID-19 sepsis caused by SARS-CoV-2 preferentially results in pulmonary pathology [43]], the Shiga toxin produced by EHEC interacts predominantly with its receptors enriched in the kidneys and endothelium and leads to acute renal failure. [38]]. In both diseases, however, there are also extrapulmonary [44]] or extrarenal [35]] organ manifestations.

3.5 Aims and objectives of the study

The present project aims to fundamentally and systematically expand the clinical and molecular understanding of severe COVID-19 disease courses, including the medium- and long-term course of the disease, using clinical investigation methods and state-of-the-art laboratory chemical analysis techniques. Taking into account the current situation and development of the COVID-19 pandemic, in particular the number of infections and the frequency of severe courses, the project is designed as a multicentre prospective clinical study.

The primary study objective is to examine mortality differences in patients with COVID-19 sepsis with or without the presence of septic cardiomyopathy 3 months after sepsis onset. Patients with influenza sepsis will be prospectively studied as a comparison group.

Furthermore, the disease course of patients with COVID-19 sepsis will subsequently be directly and systematically compared with the datasets of sepsis patients and healthy volunteers already generated within the NWG Translational Septomics project (cohort study *"Identification of cardiovascular and molecular prognostic factors for mid- and long-term morbidity and mortality in sepsis"*, acronym: ICROS, DRKS00013347, NCT03620409, [45]).

4 Study endpoints

The overall aim of this prospective multicenter cohort study in German intensive care units is to identify cardiovascular and molecular prognostic factors for mid-term morbidity and mortality after COVID-19-associated sepsis as a basis for the development of targeted personalized strategies.

To achieve this goal, the following endpoints are defined:

Primary endpoints

- Mortality differences between COVID-19 sepsis patients with vs. without the presence of septic cardiomyopathy at the time point 3 months after initial diagnosis of COVID-19 sepsis (T_5)

Secondary endpoints

- Mortality differences between COVID-19 sepsis patients with vs. without the presence of septic cardiomyopathy at the 6-month time point after initial diagnosis of COVID-19 sepsis (T_5)
- Incidence of cardiovascular events in patients with COVID-19 sepsis in the acute (T_1 , T_2), post-acute (T_3 -T_4) and long-term (T_5 -T_6) course.
- Differences in the incidence of septic cardiomyopathy during the acute course of illness (T_1 , T_2) in patients with COVID-19 sepsis and patients with influenza sepsis
- Differences in the incidence of cardiovascular events in patients with COVID-19 sepsis and patients with influenza sepsis in the acute (T_1 , T_2), post-acute (T_3 -T_4) and long-term (T_5 -T_6) course.

Further clinical questions to be *answered will compare patients with COVID-19 sepsis and patients with influenza sepsis as well as pre-existing cohorts of patients with non-COVID-19 associated sepsis and healthy volunteers. For this purpose, the following outcome measures will be analyzed: cardiovascular risk factors and function,* liver/kidney stiffness, *organ dysfunction, immune status, metabolome, lipidome and general functional level*:

- Frequency differences in the occurrence of septic cardiomyopathy between patients with COVID-19 sepsis and non-COVID-19-associated sepsis during the acute course of illness (cumulative T_1 and T_2).
- Identification of COVID-19-specific clinical and molecular alterations of the acute and post-acute disease course
- Analysis of potential group differences of the acute and post-acute course of disease (total collective and stratified by presence of septic cardiomyopathy) to identify parameters with potential diagnostic relevance in COVID-19 sepsis.
- Characterization of the acute and post-acute course of disease in patients with COVID-19 sepsis (total collective and stratified by presence of septic cardiomyopathy) to explore potential surrogate parameters for the occurrence of cardiac dysfunction.
- Comparison of the incidence of right heart failure during the acute course of illness (T_1 and T_2) in patients with COVID-19-associated sepsis and influenza-associated sepsis.
- Impact of right heart failure on mortality and morbidity after COVID-19-associated and influenza-associated sepsis, respectively.
- Analysis of potential group differences in mid- (T_5) and long-term (T_6) disease progression in patients with COVID-19 sepsis (overall collective and stratified by presence of septic cardiomyopathy) and comparison cohorts to identify potential biomarkers of acute and post-acute disease progression and thus potential therapeutic targets for further preclinical investigation.
- To determine the mid- and long-term morbidity in patients with COVID-19 sepsis in terms of exercise capacity or exercise limitation, quality of life, and type and frequency of cardiovascular events after ITS admission (total collective and stratified by presence of septic cardiomyopathy).
- To identify potential predictors of medium- (T_5) and long-term (T_6) mortality and morbidity after COVID-19 sepsis. The analyses focus on data obtained in the acute (T_1 , T_2) and post-acute (T_3) treatment settings.

Table 2. Overview of the studies, study parameters and operationalizations.

Domain		Subdomain	*Operationalisation / concrete survey method*
**1.**	**Screening**	Inclusion and exclusion criteria, consent form	*Examination of the criteria, obtaining consent, inclusion in the study, obtaining subsequent consent from incapacitated patients if necessary.*
**2.**	**Demographics, anamnestic information, medical history**	Demographics/ additional demographic information	*Age, sex, height, weight, hospital/ITS admission data, type of referral, place of stay prior to admission, previous ITS treatments, pre-existing tracheostoma/ventilation requirement, degree of disability, nursing level, incapacity to work, retirement.*
		Comorbidities	*Charlson Comorbidity Index*
		cardiovascular risk factors	*including arterial hypertension, diabetes (incl. HbA1c), dyslipoproteinaemia (incl. LDL-/HDL-/ cholesterol and triglycerides),*
		previous cardiovascular diseases and events prior to the diagnosis of sepsis	*Home medication, previous cardiological findings (ECG, TTE/TEE), arterial hypertension, coronary heart disease, angina pectoris, myocardial infarction, reduced ejection fraction, cardiac insufficiency, cardiac arrhythmias, valvular heart disease, pAVK, cerebrovascular events*
		Operation	*in the current hospitalization*
**3.**	**Infection data**	Infection	*Degree of protection, origin, localization*
		Criteria of sepsis / septic shock (old and new criteria)	*SIRS criteria, organ dysfunction, septic shock*
		Microbiology	*Pathogen, pathogen of sepsis, resistance*
		Secondary infection	*Degree of protection, localization*
**4.**	**clinical scores**	SOFA	*Routine laboratory diagnostics, multiple survey*
		APACHE II	*Routine laboratory diagnostics, one-time survey*
		SAPS II	*Routine laboratory diagnostics, one-time survey*
		CAM-ICU	*multiple survey*
		COVID hyperinflammation score [46]]	*Laboratory diagnostics, imaging, fever*
**5.**	**Routine laboratory / Surrogate parameter organ dysfunction**	cardiovascular system	*proBNP, BNP, Troponin*
		renal system	*Creatinine, creatinine clearances*
		hepatic system	*ASAT, ALAT, AP, CHE, Gamma-GT, GLDH, Quick, Bilirubin, Albumin*
		respiratory system	*ABG, lactate*
		Hematology	*small BB, large BB*
		Inflammation	*CRP, PCT, leukocytes, IL-6, ferritin...*
**6.**	**physiological parameters / Surrogate parameter organ dysfunction**	physiological parameters	*Heart rate, blood pressure, body temperature, respiratory rate*
		respiratory system	*Ventilation, ABG*
		Neurology	*Patient status, GCS, delirium, butyrylcholinesterase, acetylcholinesterase POCT[1]*
		renal system	*Urinary excretion, ANV, renal replacement therapy*
**7.**	**study-related laboratory parameters**	Metabolome and lipidome	*mass spectrometric analysis of steroids, acylcarnitines, amino acids and biogenic amines, monosaccharides, sphingolipids, glycerophospolipids, bile acids, leukotrienes, prostaglandins*
		Transcriptome and proteome	*including transcriptome analyses of peripheral blood mononuclear cells (PBMCs)*
		endothelial barrier disruption	*Immunoglobulins, growth factors, cell adhesion molecules, carrier proteins of blood coagulation, glycocalix markers, mitochondrial DNA*
		Immune status	*Cytokines, chemokines, immunophenotyping, glycoproteomics, epigenetic regulation of immune cells*
		Surrogate parameter Infection	*PCT, CRP*
		Microbiome[1]	*Shotgun metagenomic sequencing*

**8.**	**clinical investigations**	cardiac function	*TTE/TEE (physiological parameters, dimension, function, hemodynamics)*
		Liver and kidney stiffness	*Fibroscan®[1]*
		Hemodynamic monitoring	*PiCCO/PAC (only performed as part of routine diagnostics)*
**9.**	**Hospital treatment discharge data / Concomitant medication**	Sepsis treatment	*Duration, volume therapy, vasopressors and inotropics, ventilation, antibiosis, sepsis-specific therapy*
		Hospital treatment	*Duration, condition at discharge, survival status*
		Medical history and patient history	*Implants, nicotine behaviour, physical activity prior to CHI intake*
		Progressive medical history	*Whereabouts*
		Patient status	*Cause of death, time of hospital discharge, discharge to, weight, days on ventilator, days on renal replacement procedures, days on vasopressors, other organ replacement procedures, other hospital/ITS treatments.*
		Cardiovascular events after diagnosis of sepsis	*Cardiopulmonary resuscitations, arterial hypertension, coronary heart disease, angina pectoris, myocardial infarction, heart failure, cardiac arrhythmias, valvular heart disease, pAVK, cerebrovascular events*
**10.**	**Long-term effects and quality of life**	Long-term effects	*Telephone interview/questionnaire (screening questions, ADL/IADL, anxiety and depression symptoms, dyspnea, fatigue, PTSD, pain history and assessment of cognitive impairment using the t-MoCa)*
		Quality of life	*EQ-5D-3L (retrospective and current status)*

[1] optional implementation in individual study centers (e.g. Jena University Hospital)

5 Study population

The primary study populations consist of patients with COVID-19 sepsis (according to sepsis 3 criteria) who are receiving intensive care and are of legal age, as well as a group of patients with influenza sepsis (according to sepsis 3 criteria) who are receiving intensive care and are of legal age. Within the scope of the multicenter study project, the inclusion of 160 patients per group is planned.

5.1 Inclusion criteria

- Age ≥ 18 years
- written informed consent of the patient or his legal representative available
- proven SARS-CoV-2 infection or proven influenza virus infection
- respiratory signs
- Indication for ITS therapy
- Sepsis or septic shock according to sepsis-3 criteria
- first infection-related organ dysfunction (= diagnosis of sepsis) not older than 4 days (first blood sample taken within 4 days of sepsis onset)

5.2 Exclusion criteria

- cardiac surgery ≤ 12 months
- significant cardiac disease
    - Endocarditis
    - higher-grade valvular disease (severe/grade 3 valvular disease, symptomatic aortic stenosis, moderate mitral regurgitation with impaired ejection fraction or clinical symptoms)
    - complex structural congenital heart disease (e.g. TGA, tetralogy of Fallot, endocardial cushion defects, etc.)
    - hemodynamically relevant shunt
    - pre-existing, significant limitations of cardiac output (ejection fraction < 45 % or 10 % below normal)
    - pre-existing pulmonary hypertension
    - Z. n. myocardial infarction (≤ 1 year)
    - Post-heart transplantation
- cardiopulmonary resuscitation within the last 4 weeks before sepsis onset
- Z. n. pneumectomy
- Liver cirrhosis Child C
- Contraindication for TEE (e.g. esophageal resection, higher grade esophageal varices) and insufficient sound conditions for TTE
- pre-existing chronic terminal renal failure with dialysis
- Sepsis within the last 8 months
- Pregnancy/breastfeeding
- Therapy restriction or cessation
- Life expectancy ≤ 6 months due to secondary diseases
- previous participation in this study

5.3 Definition of sepsis/septic shock criteria (Sepsis 3 criteria)

Diagnostic criteria for sepsis and septic shock [1]]

<u>I. Detection of infection</u>

Diagnosis of infection by microbiological evidence or by clinical criteria.

II. organ dysfunction

Acute change in SOFA score ≥ 2 points due to infection (see Appendix).

III Septic shock

Persistent hypotension with vasopressor use to maintain mean arterial blood pressure ≥ 65 mmHg and serum lactate > 2 mmol/l (18 mg/dl). Hypotension persists despite adequate volume administration.

5.4 Definition of the criteria septic cardiomyopathy

In this study, septic cardiomyopathy is defined as systolic dysfunction determined by reduced left ventricular ejection fraction according to the recommendations of the *American Society of Echocardiography* and the *European Association of Cardiovascular Imaging* (< 52% in men and < 54% in women) at T_1 and/or T_2 in a patient with sepsis [47-51]) or, in the presence of mildly impaired ejection fraction, a reduction of at least 10% from baseline at T_1 and/or T_2 . Since no binding criteria for septic cardiomyopathy exist, this definition may be modified according to new findings.

6 Study Outline

Screening and inclusion of intensive care patients with COVID-19 sepsis or influenza-associated sepsis will take place in the intensive care units of the participating study centers. Some of the study-related examinations, especially for the post-acute visits T_2 and T_3 , will take place in the normal wards of the participating study centers. The follow-up visits (T_4 - T_6) will be conducted by telephone interviews or questionnaire surveys.

6.1 Sepsis patients (COVID-19 and influenza)

6.1.1 Screening and patient identification list

Patients treated in one of the intensive care units of the participating study centers will be screened daily for the presence of the above criteria for the diagnosis of COVID-19 sepsis or influenza-associated sepsis. Patients meeting the inclusion criteria will be listed in the Screening Log, patients included in the study will be listed in the Patient Identification List.

6.1.2 Informed consent

Participation in the study is voluntary. The patients are informed by means of the information leaflet provided and by a discussion with a physician of the study team. Consent is given in writing on the form provided. The consent form must be submitted in duplicate. One copy remains at the study center and must be kept in the study center file (Investigator Site File - ISF) for at least 10 years after the end of the study. The second copy will be given to the consenting party together with the patient information.

6.1.2.1 Consenting patients

Patients who are capable of giving consent must be informed verbally and in writing about the objectives, duration, procedure, benefits and all risks of the study before the study begins. The physician providing the information must ensure that the patient has understood the information. After the information has been provided, each patient is given sufficient time and opportunity to clarify any unanswered questions and to decide whether or not to participate. Each patient signs and dates his or her consent to participate in the study in writing on the informed consent form. If a patient who is capable of giving consent is unable to sign the consent form personally, a witness, who must not be a member of the study team, must be present during the informed consent process. This witness confirms the oral information and consent of the patient by date and signature.

6.1.2.2 Non-consenting patients

Due to the severity of the disease, it can be assumed that the majority of the study patients to be included are non-consenting patients. In this case, it is not possible to obtain the patient's informed consent prior to the start of data collection for the study. For this reason, written consent must be obtained from a patient's legal guardian or proxy to allow study participation. Primarily non-consenting patients, if they are able to consent during the course, will be informed verbally and in writing subsequently about participation in the study and asked for consent. In the case of non-consenting patients without a proxy or legal guardian, a re-evaluation of the capacity to consent or the appointment of a legal guardian will take place. If consent to study participation (in person or through an authorized representative/legal guardian) is not possible, an independent physician can decide on study participation as a counselling physician arrangement. As soon as an authorized representative is available or a legal representative has been appointed, the patient's consent to study participation will be obtained as soon as possible. If the patient becomes capable of giving consent, his or her consent to participate in the study will be obtained.

6.1.2.3 No or withdrawal of consent

Patients without informed consent or consent of a consultant will not be included in the study. Due to the longitudinal design, inclusion at a later time point is not possible. The patient or his authorized representative or legal guardian may withdraw consent and discontinue the study at any time and without giving reasons. The time of withdrawal of consent will be documented. In addition, the patient will be asked whether any study data already collected may be further used in the study evaluation. If the patient does not agree to the further use of the data and samples, the samples will be destroyed at the end of the study and all patient data already collected will not be included in the evaluation.

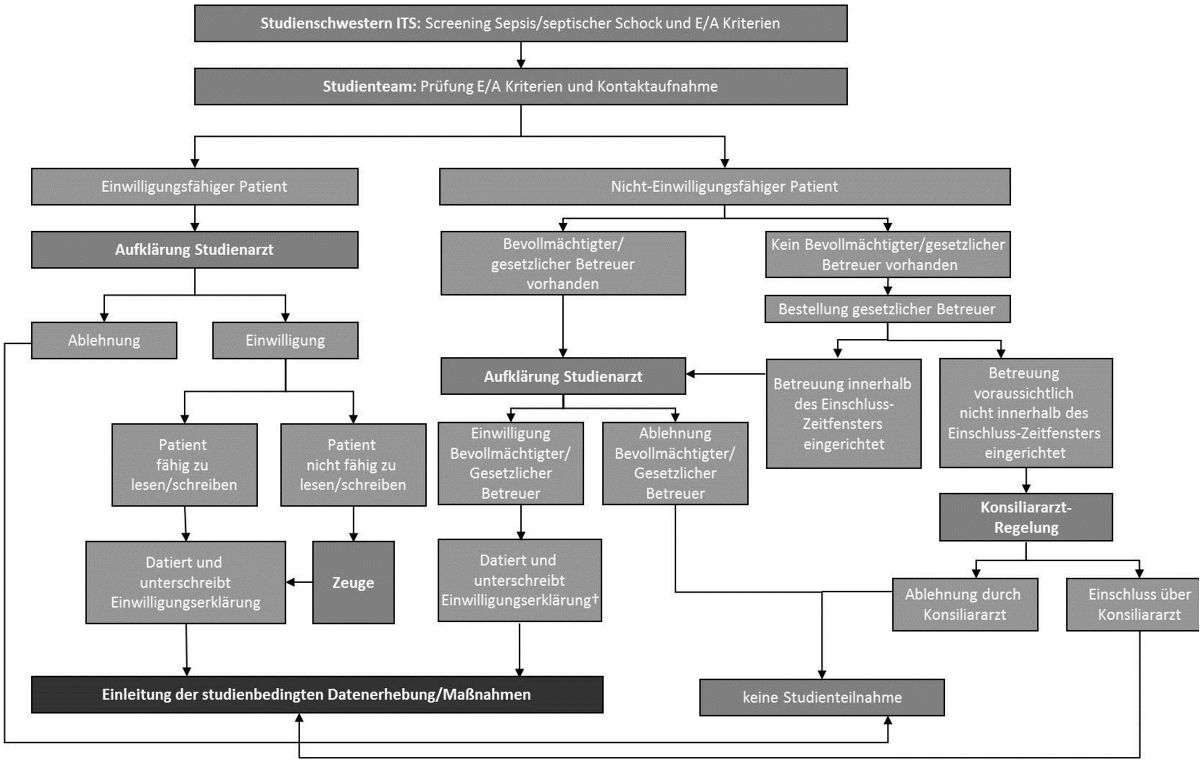

Figure 2. Flowchart of informed consent in COVID-19 sepsis patients or patients with influenza-associated sepsis.

† Primarily non-consenting patients, if capable of giving consent during the course, will be subsequently informed verbally and in writing about participation in the study and asked for consent.

6.1.3 Documentation in the course of the study

The following survey time points are planned: 3 ± 1 d (T_1), 7 ± 1 d (T_2), and 14 ± 1 d (T_3) after initial diagnosis of COVID-19-associated sepsis or influenza-associated sepsis. If the patient is scheduled for hospital discharge earlier than 14 d, the time point T_3 will be up to 3 days before discharge. Follow-up visits will be conducted via telephone interviews and questionnaires. The contents of the visits are described in **section 1.3** as well as **Table 2** in **section 4** listed. Data points from telephone interviews and questionnaires can also be collected retrospectively at a later time as a case-by-case decision.

6.1.4 Study-related measures

Study-related laboratory tests will be performed at visits T_1 , T_2 and T_3 . Echocardiographic examinations and optional transient elastography will be performed at visits T_1 and T_2 . Telephone interviews and questionnaire surveys will be performed at visits T_4 to T .$_6$

6.1.5 End of study / end of follow-up

The regular end of the study for each patient is the completion of the last follow-up visit or death in the ICU/hospital or after hospital discharge. Withdrawal of informed consent may terminate a patient's study participation prematurely. If contact with the patient cannot be established after discharge from the hospital, the study is also terminated prematurely for the patient ("lost to follow-up").

6.2 Evaluation phase

After completion of the data collection, an analysis and publication phase takes place. After all open queries have been answered and data management has been completed, the database is closed and handed over for evaluation.

7 Description of the methods

7.1 Clinical examinations

7.1.1 TEE/TTE

Echocardiography is currently the most important examination procedure for the assessment of cardiac structure and function that can be performed at the bedside. The parameters to be examined are listed in detail in the CRF. In the patients in this study, such an assessment of cardiac function is regularly indicated in the context of the underlying disease and does not then represent a study-related measure. By default, TTE is performed, which is not associated with any relevant risks. Only in case of insufficient transthoracic ultrasound conditions, e.g. due to edema formation in the course of the disease, a TEE is performed. TEE can very rarely cause injury to the oesophagus, larynx or teeth. The local anesthetic used to numb the throat can cause an allergic reaction. Pharyngeal anesthesia may cause swallowing of fluid or food debris into the trachea. If a sedative is used, intolerance reactions or impaired breathing are possible. Rarely, cardiac arrhythmias occur. The probes used are cleaned according to the legal guidelines and the specifications of our Institute for Hygiene. A transmission of pathogens is therefore very unlikely. During the TTE and TEE examinations a simple ECG with three electrodes is applied. In rare cases, skin reactions may occur in the area of the adhesive electrodes.

7.1.2 Transient elastography

Transient elastography" (syn. Fibroscan®) is an ultrasound-based, non-invasive procedure to estimate the stiffness of the liver, which correlates well with the degree of fibrosis in liver diseases. The examination is used as an

established procedure in patients with chronic liver disease, such as nonalcoholic fatty liver, to assess progression. In the context of septic diseases, damage to the liver often occurs. Currently, this damage is primarily assessed by means of laboratory parameters such as bilirubin and transaminases. The current study will therefore additionally investigate whether measurement by means of transient elastography is a suitable instrument for detecting liver damage in patients with sepsis, or whether it cannot be used reliably in these patients.
The examination is performed at the bedside and takes about five minutes per measurement. As it is an ultrasound-based procedure, the examination is not associated with any increased risk for the patients and also does not represent any stress for the patients.

7.2 Laboratory tests

7.2.1 survey of routine parameters

Within the scope of the daily laboratory tests on the ICU and normal ward, infection parameters, laboratory values for the assessment of organ functions and a blood count of the last 24 hours are regularly documented. These are not study-related measures. This also applies to the rounds that potentially take place on the normal ward.

7.2.2 Study-related examinations

For the laboratory chemical analyses, the collection of approx. 60 ml of blood is planned. The blood collection is carried out by trained specialists and according to the established sample collection scheme. If available, already applied catheters will be used. Possible side effects of blood sampling are bleeding, infections, nerve lesions or thromboses.

7.3 Health-related quality of life and long-term outcomes

Table 3 summarizes the contents of the telephone interviews and the other procedures, respectively.

Table 3. Summary of patient examinations.

Investigation	Duration
**clinical investigations**	
TEE/TTE	45 min
transient elastography[1]	5 min
**Laboratory tests**	
Blood and urine collection	5 min
**Telephone interviews**	
Anamnesis/procedural medical history	10 min
cardiovascular events since hospital discharge	5 min
cognitive performance: t-MoCa [52]]	10 min
**Questionnaire**	
Screening questions (e.g. olfactory and gustatory disorders)	5 min
Activities of daily living (ADL): Barthel Index [53]]	5 min
Anxiety and depression symptomatology: BSI-18 [54]]	5 min
Fatigue: Fatigue Scale [55]]	5 min
instrumental activities of daily living (IADL) [56]]	5 min
Quality of life: EQ-5D-3L [57]]	5 min
Symptoms post-traumatic stress disorder: PTSS-14 [58]]	5 min
Pain history: e.g. Korff Graded Chronic Pain Scale [59]]	5 min
Total duration questionnaire:	*40 min*

[1] *optional implementation in individual study centers (e.g. Jena University Hospital)*

8 Adverse events

Adverse events are not expected.

9 Data management and quality assurance

9.1 Patient Identification List

All patient-related data are recorded in pseudonymised form. For this purpose, a non-speaking pseudonym is used, from which alone the identity of the patient cannot be inferred.
The study centers maintain a patient identification list in which the patient identification numbers are linked to the full patient names of the participants and date of birth. This must be filed in the study folder. The list serves the possibility of later identification of participating persons. It must be kept absolutely confidential and must not leave the data collection centre. It must be archived for **at least ten years** after the end of the study. In addition, the study participation or the planned inclusion in the study is noted in the patient file.

9.2 List of responsibilities

It must be ensured that each person responsible for the documentation in the eCRF can be identified. A list with signature and abbreviation of the persons who are allowed to make entries in the eCRF (Signature/Delegation Log) is stored in the ISF and in the TMF. This overview is also used to identify other persons involved in the study with their names, signatures and abbreviations as well as their responsibilities and authorities.

9.3 Data collection/documentation forms

To achieve the study objective, it is necessary to collect and process medical data of individual patients. The data relevant for the clinical study are collected via RDE (Remote Data Entry). For this purpose, the data are entered by an authorized member of the study center at an online connected workstation computer into special masks, which represent an electronic CRF. Via the electronic CRF, the data are directly transferred to the study database in the ZKS Jena. It is the responsibility of the head of the respective study center to ensure that all data collected during the clinical trial are entered correctly and completely into the database created specifically for this clinical trial. Corrections in the eCRF may only be made by authorized persons and must be justified.

A paper-based CRF as a "viewing copy" will be given to the study centres as part of the ISF. The relevant staff will be given instructions on how to complete the electronic documents.

9.4 Data processing

The data collection serves scientific purposes. The data are generated at the study center. All collected medical data are entered by the corresponding staff members in a computer-based online data entry system and immediately transferred to the servers at the Center for Clinical Studies Jena. The data entry is done via web application on the servers of the ZKS of the Jena University Hospital into the study management software "OpenClinica®". The software fulfils the regulatory requirements (GCP, 21 CFR Part 11). The data are collected via an encrypted data connection (HTTPS) in input masks using a web browser. To ensure pseudonymous data analysis, a unique patient identification number is assigned to each patient.

The study management software "OpenClinica®" is also used for data management. The accuracy of the data is checked by range, validity and consistency checks. Non-plausible or missing data are queried at the study centre. Every change to the data, e.g. due to the incorporation of answered queries, is documented in the database via an automatic change tracking (audit trail). The use of a hierarchical, role-based access concept makes unauthorized access to the study data impossible.

9.5 Retention of study documents

The Center for Clinical Studies at the Jena University Hospital is appointed as a documentation center also for data storage. The backup of electronic data happens regularly. The data storage facilities are in a locked, central room to which only system administrators have access.

The study director must ensure that key records are retained for at least 10 years after the study has ended. Other regulations for the retention of medical records remain unaffected. All records must be kept in a secure location and treated confidentially. If necessary (e.g., due to legal requirements or after consultation with the study director), records may be retained beyond the above period. Records and documents related to the study, e.g. the patient identification list, informed consent forms, correspondence with the Ethics Committee, the competent authorities, the study director and other relevant documents, must be kept at the study centres for at least 10 years (or longer if required by law). The study centre must take precautions to prevent accidental or premature destruction of these documents.

9.6 Data protection

As part of the study, it is necessary to collect and process personal data from the study participants (e.g. full name, initials of first and last name, date of birth, address) and data on treatment and the course of the disease (e.g. medical findings, types of treatment, prescribed medication). These data are collected at the study center and stored electronically in pseudonymized form (i.e. without direct reference to the patient's name) with the help of a patient identification number, transmitted to the responsible data processing agency and evaluated.

In the event of a revocation of consent to the study by the patient, including further data collection, no further data will be collected from the time of revocation. The previously collected data will only be further used and evaluated within the study if permission has been granted by the former study participant.

10 Biometrics

10.1 Endpoints

The primary endpoint is mortality differences between COVID-19 sepsis patients with or without the presence of septic cardiomyopathy at 3 months after initial diagnosis of COVID-19 sepsis (T_5). See **section 4** for secondary endpoints and additional questions.

10.2 Definition of evaluation cohorts

For patients with COVID-19 sepsis, there are three evaluation cohorts in the acute phase (T_1 , T_2), post-acute phase (T_3 , T_4) as well as in the medium (T_5) and long-term (T_6) course:

- Overall collective
- COVID-19 sepsis patients with septic cardiomyopathy
- COVID-19 sepsis patients without septic cardiomyopathy

For patients with influenza sepsis, there are three evaluation cohorts in the acute phase (T_1 , T_2), post-acute phase (T_3 , T_4) and in the medium (T_5) and long-term (T_6) course:

- Overall collective
- Influenza sepsis patients with septic cardiomyopathy
- Influenza sepsis patients without septic cardiomyopathy

For the control groups from the ICROS study, the evaluation cohorts are as follows:

Patients with sepsis or septic shock as defined by sepsis 3:

- Overall collective
- Sepsis patients with septic cardiomyopathy
- Sepsis patients without septic cardiomyopathy

Healthy subjects with focus on inclusion time point (T_1)

10.3 Case number planning

Case number estimation is based on similar trade-offs to those in the ICROS study [45]]. The focus of planning is on mortality differences at 3 months. If a simple $\chi 2$-test is applied at a two-sided significance level of $\alpha = 5$ %, a cohort size of n = 80 patients per group is sufficient to detect differences (absolute risk reduction) in 3-month mortality of ≥ 22 % with a statistical power of ≥ 80 %, assuming a 3-month mortality of ICU-treated COVID-19 patients without cardiomyopathy of 50 % (similar to mortality in septic shock). For the case of a lower 3-month mortality in the group of ICU-treated COVID-19 patients without cardiomyopathy of, for example, 40%, similarly large differences (absolute risk reduction) can also be demonstrated with a power ≥ 80%. Case number considerations were performed using the power.prop.test function in R (version 4.0.2).

10.4 Interim/evaluation

No interim evaluation of the complete data set is planned. The final evaluation will take place after the complete data sets are available 6 months after the initial diagnosis of COVID-19 sepsis. To answer specific questions, restricted data sets will be read out if necessary.

10.5 Further statistical analysis

Adequate statistical standard procedures are used to answer the secondary endpoints and other questions. In the descriptive analyses, all parameters are reported according to their scale level (relative and absolute frequencies, location and dispersion measures). Group comparisons are analyzed using adequate procedures depending on the distributional properties of the target parameters. The identification of prognostic factors or predictors is primarily of an exploratory nature. Appropriate methods of multivariate statistics, in particular correlation and regression analyses, are used.

10.6 Presentation of the results

The presentation of results is based on the STROBE criteria for the presentation of observational studies [60]] and the TRIPOD criteria [61]] for prognostic questions.

11 Publication / Use of results / Registration of data collection

11.1 Final report and publications

11.1.1 Publication of the study protocol

The final, consented study protocol will be published in a peer-reviewed journal in accordance with the practices of international study groups and the regulations listed below.

11.1.2 Final report

Interim and final reports are prepared in accordance with the requirements of the Federal Ministry of Education and Research.

11.1.3 Analyses and publications

Analyses and publications on primary and secondary questions are carried out by the study directors, scientific staff and an independent bioinformatics or statistical service provider. Local study directors retain the right to access their own data and to use and publish them without restriction.

11.2 Citation

Each publication shall be accompanied by the following:
"This study was funded by the German Federal Ministry of Education and Research (Coldewey - ICROVID: Identification of cardiovascular and molecular prognostic factors for morbidity and mortality in COVID-19 sepsis, FKZ 03COV07)."

11.3 Authors

Regarding the rights and obligations of the authors involved, the publication guidelines for authors in medical journals according to the recommendations of the ICMJE (http://www.icmje.org/ethical_1author.html) are authoritative. The final decision on authorship and order of authors is the responsibility of the study director. At a minimum, the study director may claim to be named as a co-author of a publication on broader issues, provided that he or she has made a relevant contribution to that publication.

11.4 Registration

The present study will be registered in the German Registry of Clinical Trials and in the Clinical Trials.gov registry before inclusion of the first study patient.

12 Ethical concerns and administrative arrangements

12.1 Declaration of Helsinki and Good Clinical Practice

The study will be conducted in accordance with the ethical principles that have their origin in the Declaration of Helsinki. The current version of the Declaration will be observed. The recommendations of Good Clinical Practice, valid since 17.1.1997, if applicable, will be taken into account.

12.2 Ethics Committees

The study protocol is submitted to the Ethics Committee of the Jena University Hospital together with the required further documents with the request for evaluation. The study can only begin after the Ethics Committee has given its approval. The same procedure is followed in all participating study centers.

12.3 Subsequent changes

The study protocol must be followed. Any deviation from the planned study measures or times for which the study director is responsible must be documented and justified.
Changes or additions to the study protocol can only be initiated and authorized by the study management. The first ethics committee is informed of any changes to the study protocol. If necessary, the approval of the Ethics Committee will be obtained again. Changes requiring evaluation may not be implemented before the decision of the ethics committee.
Amendments to the study approved by the Ethics Committee that are appropriate,

- have an impact on the safety of the persons concerned,
- additional data collection or analysis that requires a change in patient information and/or consent,
- influence the interpretation of the scientific documents on which the study is based or the scientific validity of the study results,
- substantially change the way the study is managed or conducted,
- may only be made if these changes have been approved by the Ethics Committee.

12.4 Funding

The project is funded by the German Federal Ministry of Education and Research (Coldewey - ICROVID: Identification of cardiovascular and molecular prognostic factors for morbidity and mortality in COVID-19 sepsis, FKZ 03COV07) and supported by the Department of Anesthesiology and Intensive Care Medicine of the UKJ.

13 Literature

1. Singer, M., et al, *The Third International Consensus Definitions for Sepsis and Septic Shock (Sepsis-3).* JAMA, 2016. **315**(8): p. 801-10.
2. Zahar, J.R., et al, *Outcomes in severe sepsis and patients with septic shock: pathogen species and infection sites are not associated with mortality.* Crit Care Med, 2011. **39**(8): p. 1886-95.
3. Rudd, K.E., et al, *Global, regional, and national sepsis incidence and mortality, 1990-2017: analysis for the Global Burden of Disease Study.* Lancet, 2020. **395**(10219): p. 200-211.
4. Cohen, J., et al, *Sepsis: a roadmap for future research.* Lancet Infectious Diseases, 2015. **15**(5): p. 581-614.
5. WHO, *Report of the WHO-China Joint Mission on Coronavirus Disease 2019 (COVID-19)*. 2020.
6. Cohen, J., et al, *Sepsis: a roadmap for future research.* Lancet Infect Dis, 2015. **15**(5): p. 581-614.
7. Wang, R.F. and H.Y.. Wang, *Immune targets and neoantigens for cancer immunotherapy and precision medicine.* Cell Res, 2017. **27**(1): p. 11-37.
8. Lewis, A.J., J.S. Lee, and M.R. Rosengart, *Translational sepsis research: spanning the divide.* Crit Care Med, 2018. **46**(9): p. 1497-1505.
9. Seymour, C.W., et al, *Derivation, Validation, and Potential Treatment Implications of Novel Clinical Phenotypes for Sepsis.* JAMA, 2019. **321**(20): p. 2003-2017.
10. Huang, C., et al, *Clinical features of patients infected with 2019 novel coronavirus in Wuhan, China.* Lancet, 2020. **395**(10223): p. 497-506.
11. Hornuss, D., et al, *Anosmia in COVID-19 patients.* Clin Microbiol Infect, 2020.
12. Guzik, T.J., et al, *COVID-19 and the cardiovascular system: implications for risk assessment, diagnosis, and treatment options.* Cardiovasc Res, 2020.
13. Ackermann, M., et al, *Pulmonary Vascular Endothelialitis, Thrombosis, and Angiogenesis in Covid-19*. N Engl J Med, 2020. **383**(2): p. 120-128.
14. Michael Henry, B., et al, *Hyperinflammation and Derangement of Renin-Angiotensin-Aldosterone System in COVID-19: a novel hypothesis for clinically suspected hypercoagulopathy and microvascular immunothrombosis.* Clin Chim Acta, 2020.
15. Connors, J.M. and J.H. Levy, *COVID-19 and its implications for thrombosis and anticoagulation.* Blood, 2020.
16. Warren-Gash, C., L. Smeeth, and A.C. Hayward, *Influenza as a trigger for acute myocardial infarction or death from cardiovascular disease: a systematic review.* Lancet Infect Dis, 2009. **9**(10): p. 601-10.
17. Kindermann, I., et al, *Predictors of outcome in patients with suspected myocarditis.* Circulation, 2008. **118**(6): p. 639-48.
18. Morens, D.M., J.K. Taubenberger, and A.S. Fauci, *Predominant role of bacterial pneumonia as a cause of death in pandemic influenza: implications for pandemic influenza preparedness.* J Infect Dis, 2008. **198**(7): p. 962-70.
19. Sellers, S.A., et al, *The hidden burden of influenza: A review of the extra-pulmonary complications of influenza infection.* Influenza Other Respir Viruses, 2017. **11**(5): p. 372-393.
20. Hendren, N.S., et al, *Description and Proposed Management of the Acute COVID-19 Cardiovascular Syndrome.* Circulation, 2020. **141**(23): p. 1903-1914.
21. Yang, X., et al, *Clinical course and outcomes of critically ill patients with SARS-CoV-2 pneumonia in Wuhan, China: a single-centre, retrospective, observational study.* Lancet Respir Med, 2020. **8**(5): p. 475-481.
22. Shi, S., et al, *Association of Cardiac Injury With Mortality in Hospitalized Patients With COVID-19 in Wuhan, China.* JAMA Cardiol, 2020.
23. Lippi, G., C.J. Lavie, and F. Sanchis-Gomar, *Cardiac troponin I in patients with coronavirus disease 2019 (COVID-19): Evidence from a meta-analysis.* Prog Cardiovasc Dis, 2020.
24. Bilaloglu, S., et al, *Thrombosis in Hospitalized Patients With COVID-19 in a New York City Health System.* JAMA, 2020.
25. Klok, F.A., et al, *Incidence of thrombotic complications in critically ill ICU patients with COVID-19.* Thromb Res, 2020. **191**: p. 145-147.
26. Middeldorp, S., et al, *Incidence of venous thromboembolism in hospitalized patients with COVID-19.* J

Thromb Haemost, 2020.
27. Tang, N., et al, *Anticoagulant treatment is associated with decreased mortality in severe coronavirus disease 2019 patients with coagulopathy.* J Thromb Haemost, 2020. **18**(5): p. 1094-1099.
28. Lindner, D., et al, *Association of Cardiac Infection With SARS-CoV-2 in Confirmed COVID-19 Autopsy Cases.* JAMA Cardiol, 2020.
29. Sato, R. and M. Nasu, *A review of sepsis-induced cardiomyopathy.* J Intensive Care, 2015. **3**: p. 48.
30. Fakhouri, F., et al, *Haemolytic uraemic syndrome.* Lancet, 2017. **390**(10095): p. 681-696.
31. Matthies, J., et al, *Extrarenal manifestations in shigatoxin-associated hemolytic uremic syndrome.* Clin Padiatr, 2016. **228**(4): p. 181-8.
32. Askiti, V., et al, *Troponin I levels in a hemolytic uremic syndrome patient with severe cardiac failure.* Pediatr Nephrol, 2004. **19**(3): p. 345-8.
33. Birk, P.E., et al, *Cardiac tamponade as a terminal event in the hemolytic uremic syndrome in childhood.* Pediatr Nephrol, 1994. **8**(6): p. 754-5.
34. Palanca Arias, D., M. Lopez Ramon, and L. Jimenez Montanes, *Biomarkers detect involvement of acute myocardial injury in a paediatric haemolytic-uraemic syndrome patient.* Cardiol Young, 2016. **26**(5): p. 983-6.
35. Khalid, M. and S. Andreoli, *Extrarenal manifestations of the hemolytic uremic syndrome associated with Shiga toxin-producing Escherichia coli (STEC HUS).* Pediatr Nephrol, 2019. **34**(12): p. 2495-2507.
36. Gallo, E.G. and C.A. Gianantonio, *Extrarenal involvement in diarrhea-associated haemolytic-uraemic syndrome.* Pediatr Nephrol, 1995. **9**(1): p. 117-9.
37. Karmali, M.A., et al, *The association between idiopathic hemolytic uremic syndrome and infection by verotoxin-producing Escherichia coli.* J Infect Dis, 1985. **151**(5): p. 775-82.
38. Boyd, B. and C. Lingwood, *Verotoxin receptor glycolipid in human renal tissue.* Nephron, 1989. **51**(2): p. 207-10.
39. Zoja, C., S. Buelli, and M. Morigi, *Shiga toxin-associated hemolytic uremic syndrome: pathophysiology of endothelial dysfunction.* Pediatr Nephrol, 2010. **25**(11): p. 2231-40.
40. Endo, Y., et al., *The mechanism of action of ricin and related toxic lectins on eukaryotic ribosomes. The site and the characteristics of the modification in 28 S ribosomal RNA caused by the toxins.* J Biol Chem, 1987. **262**(12): p. 5908-12.
41. Thoms, M., et al, *Structural basis for translational shutdown and immune evasion by the Nsp1 protein of SARS-CoV-2.* Science, 2020.
42. Iba, T., et al, *Sepsis-associated disseminated intravascular coagulation and its differential diagnoses.* J Intensive Care, 2019. **7**: p. 32.
43. Gavriatopoulou, M., et al, *Organ-specific manifestations of COVID-19 infection.* Clin Exp Med, 2020.
44. Gupta, A., et al, *Extrapulmonary manifestations of COVID-19.* Nat Med, 2020. **26**(7): p. 1017-1032.
45. Coldewey, S.M., et al, *Identification of cardiovascular and molecular prognostic factors for the medium-term and long-term outcomes of sepsis (ICROS): protocol for a prospective monocentric cohort study.* BMJ Open, 2020. **10**(6): p. e036527.
46. La Rosee, F., et al, *The Janus kinase 1/2 inhibitor ruxolitinib in COVID-19 with severe systemic hyperinflammation.* Leukemia, 2020. **34**(7): p. 1805-1815.
47. Berrios, R.A.S., et al, *Correlation of left ventricular systolic dysfunction determined by low ejection fraction and 30-day mortality in patients with severe sepsis and septic shock: A systematic review and meta-analysis.* Journal of Critical Care, 2014. **29**(4): p. 495-499.
48 Landesberg, G., et al, *Diastolic dysfunction and mortality in severe sepsis and septic shock.* European Heart Journal, 2012. **33**(7): p. 895-903.
49 Vieillard-Baron, A., *Septic cardiomyopathy.* Ann Intensive Care, 2011. **1**(1): p. 6.
50. Müller-Werdan, U., et al, *Microcirculatory dysfunction, cytopathic hypoxia and septic cardiomyopathy,* in *Sepsis and MODS,* K. Werdan, et al, Editors. 2016, Springer Science and Business Media. p. 137-139.
51. Lang, R.M., et al, *Recommendations for cardiac chamber quantification by echocardiography in adults: an update from the American Society of Echocardiography and the European Association of Cardiovascular Imaging.* J Am Soc Echocardiogr, 2015. **28**(1): p. 1-39 e14.
Pendlebury, S.T., et al, *Telephone assessment of cognition after transient ischemic attack and stroke: modified telephone interview of cognitive status and telephone Montreal Cognitive Assessment versus face-to-face Montreal Cognitive Assessment and neuropsychological battery.* Stroke, 2013. **44**(1): p. 227-9.

53. Heuschmann, P.U., et al., *[The reliability of the german version of the barthel-index and the development of a postal and telephone version for the application on stroke patients].* Fortschr Neurol Psychiatr, 2005. **73**(2): p. 74-82.
54. Spitzer, C., et al., *[The short version of the Brief Symptom Inventory (BSI -18): preliminary psychometric properties of the German translation].* Fortschr Neurol Psychiatr, 2011. **79**(9): p. 517-23.
55. Martin, A., et al., *Measurement of chronic fatigue - Teststatistical examination of the Fatigue Scale (FS).* Journal of Clinical Psychology and Psychotherapy, 2010. **39**(1): p. 33-44.
56. Lawton, M.P. and E.M. Brody, *Assessment of older people: self-maintaining and instrumental activities of daily living.* Gerontologist, 1969. **9**(3): p. 179-86.
57. Rabin, R., et al., *From translation to version management: a history and review of methods for the cultural adaptation of the EuroQol five-dimensional questionnaire.* Value Health, 2014. **17**(1): p. 70-6.
58. Radtke, F.M., et al., *[The Post-Traumatic Stress Syndrome 14-Questions Inventory (PTSS-14) - Translation of the UK-PTSS-14 and validation of the German version].* Anasthesiol Intensivmed Notfallmed Schmerzther, 2010. **45**(11-12): p. 688-95.
59. Klasen, B.W., et al., *Validation and reliability of the German version of the Chronic Pain Grade questionnaire in primary care back pain patients.* Psychosoc Med, 2004. **1**: p. Doc07.
60. von Elm, E., et al, *The Strengthening the Reporting of Observational Studies in Epidemiology (STROBE) Statement Guidelines for Reporting Observational Studies.* Epidemiology, 2007. **18**(6): p. 800-804.
61. Collins, G.S., et al, *Transparent reporting of a multivariable prediction model for individual prognosis or diagnosis (TRIPOD): the TRIPOD Statement.* Bmc Medicine, 2015. **13**.

14 Appendix

14.1 SOFA score

14.2 APACHE score

14.3 SAPS II Score

14.4 Charlson Comorbidity Index

14.5 CAM-ICU

14.6 COVID Hyperinflammation Score
